# The androgen receptor interacts with GATA3 to transcriptionally regulate a luminal epithelial cell phenotype in breast cancer

**DOI:** 10.1186/s13059-023-03161-y

**Published:** 2024-02-05

**Authors:** Leila Hosseinzadeh, Zoya Kikhtyak, Geraldine Laven-Law, Stephen M. Pederson, Caroline G. Puiu, Clive S. D’Santos, Elgene Lim, Jason S. Carroll, Wayne D. Tilley, Amy R. Dwyer, Theresa E. Hickey

**Affiliations:** 1 Dame Roma Mitchell Cancer Research Laboratories, Adelaide Medical School, University of Adelaide, Adelaide, Australia; 2 Cancer Research UK Cambridge Institute, University of Cambridge, Cambridge, UK; 3 Garvan Institute of Medical Research, University of New South Wales, Sydney, Australia

**Keywords:** Androgen receptor, GATA3, Breast, Luminal lineage

## Abstract

**Background:**

The androgen receptor (AR) is a tumor suppressor in estrogen receptor (ER) positive breast cancer, a role sustained in some ER negative breast cancers. Key factors dictating AR genomic activity in a breast context are largely unknown. Herein, we employ an unbiased chromatin immunoprecipitation-based proteomic technique to identify endogenous AR interacting co-regulatory proteins in ER positive and negative models of breast cancer to gain new insight into mechanisms of AR signaling in this disease.

**Results:**

The DNA-binding factor GATA3 is identified and validated as a novel AR interacting protein in breast cancer cells irrespective of ER status. AR activation by the natural ligand 5α-dihydrotestosterone (DHT) increases nuclear AR-GATA3 interactions, resulting in AR-dependent enrichment of GATA3 chromatin binding at a sub-set of genomic loci. Silencing GATA3 reduces but does not prevent AR DNA binding and transactivation of genes associated with AR/GATA3 co-occupied loci, indicating a co-regulatory role for GATA3 in AR signaling. DHT-induced AR/GATA3 binding coincides with upregulation of luminal differentiation genes, including *EHF* and *KDM4B*, established master regulators of a breast epithelial cell lineage. These findings are validated in a patient-derived xenograft model of breast cancer. Interaction between AR and GATA3 is also associated with AR-mediated growth inhibition in ER positive and ER negative breast cancer.

**Conclusions:**

AR and GATA3 interact to transcriptionally regulate luminal epithelial cell differentiation in breast cancer regardless of ER status. This interaction facilitates the tumor suppressor function of AR and mechanistically explains why AR expression is associated with less proliferative, more differentiated breast tumors and better overall survival in breast cancer.

**Supplementary Information:**

The online version contains supplementary material available at 10.1186/s13059-023-03161-y.

## Background

Breast cancer is now the most common malignancy worldwide, having overtaken lung cancer in prevalence in 2020 [1]. Up to 80% of cases are driven by oncogenic signaling of the estrogen receptor alpha (ER) transcription factor, clinically called ER positive (ER+) breast cancer [2]. The remaining cases lack expression of ER (ER-) and are a heterogeneous mix of clinical and molecular subtypes [3, 4]. Another hormone-activated transcription factor, the androgen receptor (AR), is more frequently expressed than ER in breast cancer, occurring in up to 95% of ER+ and ~ 20–30% of ER- primary tumors depending on detection method and criteria used to determine AR and ER positivity [5]. While the role and mechanistic pathways associated with ER signaling in breast cancer have been comprehensively described [6], understanding of AR signaling in this disease is much more limited. We recently demonstrated that AR inhibits growth and acts as a tumor suppressor in normal breast tissues and ER+ breast cancers and acts on multiple cell types to induce striking changes in the female breast that are consistent with reduced cancer risk [7, 8]. However, key factors dictating AR genomic activity in ER+ breast cancers are largely unknown. The role and mechanistic pathways associated with AR signaling in ER- disease are even more obscure and may differ depending on the ER- molecular subtype [9]. Whether AR serves a conserved function across all breast cancer subtypes is also currently unknown. Hence, a better understanding of AR signaling across disease subtypes is essential to expand basic, fundamental knowledge of its role in breast biology as well as carcinogenesis. This knowledge is critical for development of rational therapeutic strategies to target AR in breast cancer.

Sex hormone receptors, including AR and ER, are ligand-activated transcription factors that recruit a host of co-regulatory proteins that act to facilitate DNA binding or recruitment of chromatin modifiers and the transcriptional machinery in order to regulate target genes [10, 11]. Advances in technology over the past two decades has allowed genome-wide mapping of transcription factor interactions with chromatin via chromatin immunoprecipitation with massively parallel DNA sequencing (ChIP-seq) [12] and unbiased interrogation of transcription factor interactions with nuclear co-factors while bound to chromatin via rapid immunoprecipitation mass spectrometry of endogenous protein (RIME) [13]. A more profound understanding of ER signaling in breast cancer has been gleaned via use of these technologies and their derivatives [14–19]. Likewise, ChIP-seq has been used to expand understanding of AR signaling in this disease [8, 20, 21], but to date, no one has interrogated multi-subunit protein complexes involving AR in an unbiased manner in breast cancer. This has fundamentally limited the ability to define and differentiate AR action across breast cancer subtypes.

Herein, we performed AR RIME experiments to characterize and compare the AR interactomes in cell line models representing ER+ and ER- breast cancer to identify candidate co-regulators of AR signaling and forge new insight into the role and mechanistic basis of AR signaling in breast carcinogenesis.

## Results

### GATA3 is a novel AR interacting protein in breast cancer cells

**Fig. 1:**
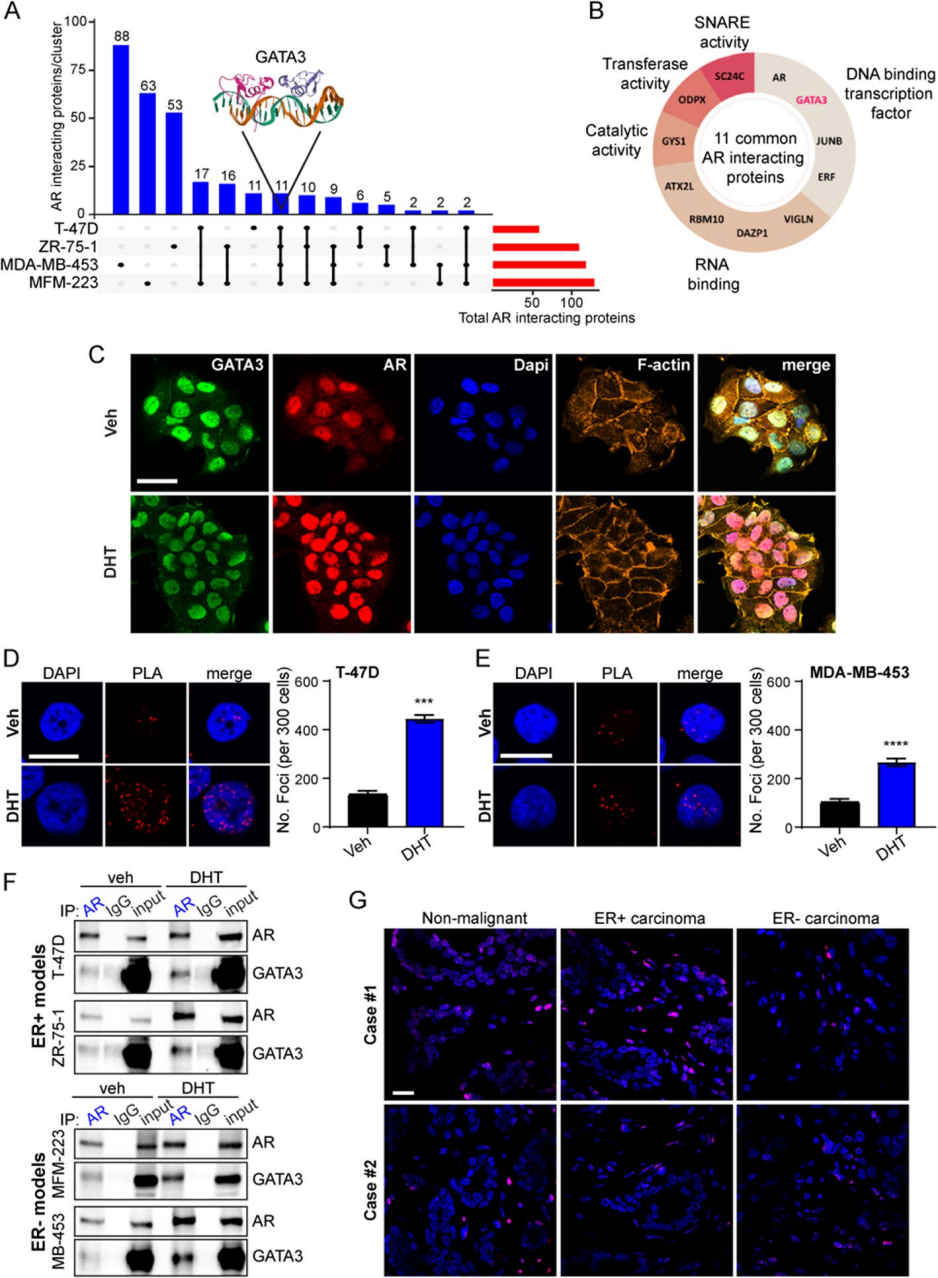
GATA3 is a novel AR interacting protein in breast cancer. **A** Up-set plot representing common and unique AR interacting proteins identified in ER+ and ER- breast cancer cell lines. **B** Classification of the 11 AR interacting proteins common to ER+ and ER- in vitro breast cancer models based on their molecular functions. **C** Multi-labeled immunofluorescence for AR (red) and GATA3 (green) in T-47D cells with nuclear (Dapi; blue) and cytoskeletal (F-actin; orange) labels. Scale bar = 30 μm. **D**, **E** Proximity ligation assays (PLA) showing AR-GATA3 interactions in **D** T-47D (ER+) and **E** MDA-MB-453 (ER-) breast cancer cell lines treated with Vehicle (Veh; EtOH) or the AR agonist 5-α-dihydrotestosterone (DHT; 10 nM). Scale bar = 15 μm. Quantification represented as mean foci per 300 nuclei ± SEM (*n* = 5 technical replicates) analyzed by one-sided unpaired *t*-test (***P < 0.001; ****P < 0.0001). **F** Western blot showing co-immunoprecipitation of AR and GATA3 in ER+ (top panel) and ER- (bottom panel) breast cancer cell lines treated with Veh (EtOH) or DHT (1 nM). **G** PLA for AR and GATA3 interaction in non-malignant and malignant (ER+ and ER-) clinical breast tissues. Scale bar = 30 μm

To agnostically profile AR interacting proteins across ER+ and ER- breast cancer models, four cell lines that encompass three molecular subtypes of breast cancer and have established AR expression were interrogated: ER+ luminal (ZR-75-1, T-47D) and two subtypes of ER- disease including molecular apocrine/HER2 + (MDA-MB-453) and triple negative (i.e., lacking ER, PR and HER2 expression; MFM-223) [22–24]. For each cell line, AR RIME experiments (endogenous IP-Mass Spec) were performed as three independent replicates representing consecutive passages of cells to identify reproducible AR protein–protein interactions at the chromatin level. A factor was selected as a high confidence candidate AR interacting protein if detected in all three AR immunoprecipitation (IP) replicates and not in the associated IgG IP negative controls (Additional file 1). In all models, AR (which homodimerizes upon ligand activation) was detected with high confidence, indicative of the specificity and reproducibility of our RIME datasets (Additional file 1). We identified a total of 110 (ZR-75-1), 59 (T-47D), 119 (MDA-MB-453), and 130 (MFM-223) candidate AR interacting proteins and compared them across models (Fig. 1A). Although model-specific interacting proteins predominated, eleven interacted with AR in all four breast cancer cell lines investigated, classified by molecular function into DNA-binding transcription factors, RNA-binding proteins, and proteins with either catalytic, transferase, or SNARE (soluble N-ethylmale-imide-sensitive factor-attachment protein receptors) activities (Fig. 1B). Of particular interest were three DNA-binding factors associated with AR on chromatin in all breast cancer contexts: GATA3, JUNB, and ERF (Fig. 1B). Interaction between AR and any one of these factors has not been previously reported in the context of breast cancer, but JUNB and ERF have been identified as AR interacting proteins in two LNCaP-derived cell line models of prostate cancer [25]. The pioneer factor GATA3 is a well-established ER interacting protein in breast cancer cells that modulates but is not required for ER to bind chromatin [17, 26, 27], but an interaction between AR and GATA3 has not been previously reported in any tissue or cellular context. Our RIME data identifies GATA3 as a novel AR interacting protein in breast cancer cells irrespective of ER status, indicating that GATA3 can function as a co-regulator of steroid receptors other than ER. Four unique GATA3 peptides were consistently detected across all cell lines in the AR RIME datasets, exclusively belonging to the *Homo sapiens* GATA3 protein (Additional file 1). Due to the critical role of GATA3 in breast development [28–31] and as a regulator of ER signaling in breast cancer [26, 29, 32], we chose to further investigate its functional interaction with AR.

Some nuclear co-localization of AR and GATA3 was observed via dual-label immunofluorescence (IF) in untreated, hormone-deprived T-47D cells, but as expected, co-localization markedly increased upon treatment with the natural AR agonist, DHT, due to ligand-induced nuclear translocation of AR (Fig. 1C). Following AR activation, nearly all cell nuclei were positive for AR and GATA3. Proximity ligation assays (PLA) confirmed nuclear interaction between the two proteins in all four cell line models investigated (Fig. 1D, E; Additional file 2: Fig. S1A-C). Consistent with the IF data (Fig. 1C), some AR-GATA3 interactions were detected by PLA under hormone-deprived conditions, but DHT treatment significantly increased the number of nuclear interactions (Fig. 1D, E; Additional file 2: Fig. S1A-C) in a time-dependent manner in all the models tested (Additional file 2: Fig. S1D). The AR-GATA3 interaction was further validated by co-immunoprecipitation (co-IP) assays in which pull down of AR was associated with detection of GATA3 in all four breast cancer cell lines upon treatment with DHT (Fig. 1F). Reciprocal pull down of GATA3 was associated with detection of AR in a DHT-dependent manner in the ER- breast cancer cell lines but not the ER+ lines (Additional file 2: Fig. S1E). We posit the latter observation is due to co-IP being markedly less sensitive than both the RIME and PLA methodologies and the ER+ breast cancer lines having substantially lower AR protein levels compared to the ER- lines (Additional file 2: Fig. S1F). PLA-detected interactions between AR and GATA3 were also evident in clinical primary ER+ /AR + (*n* = 2 cases) and ER- /AR + (*n* = 2 cases) breast cancers as well as non-malignant breast tissues from reduction mammoplasties (*n* = 2 cases) (Fig. 1G; Additional file 2: Fig. S1G, H). Collectively, these data confirm GATA3 as an AR interacting protein in breast cancer cells independent of ER status and suggest a conserved role for this interaction in non-malignant and malignant breast epithelial cells.

### AR activation induces a distinct subset of GATA3 cis-regulatory DNA binding events

**Fig. 2:**
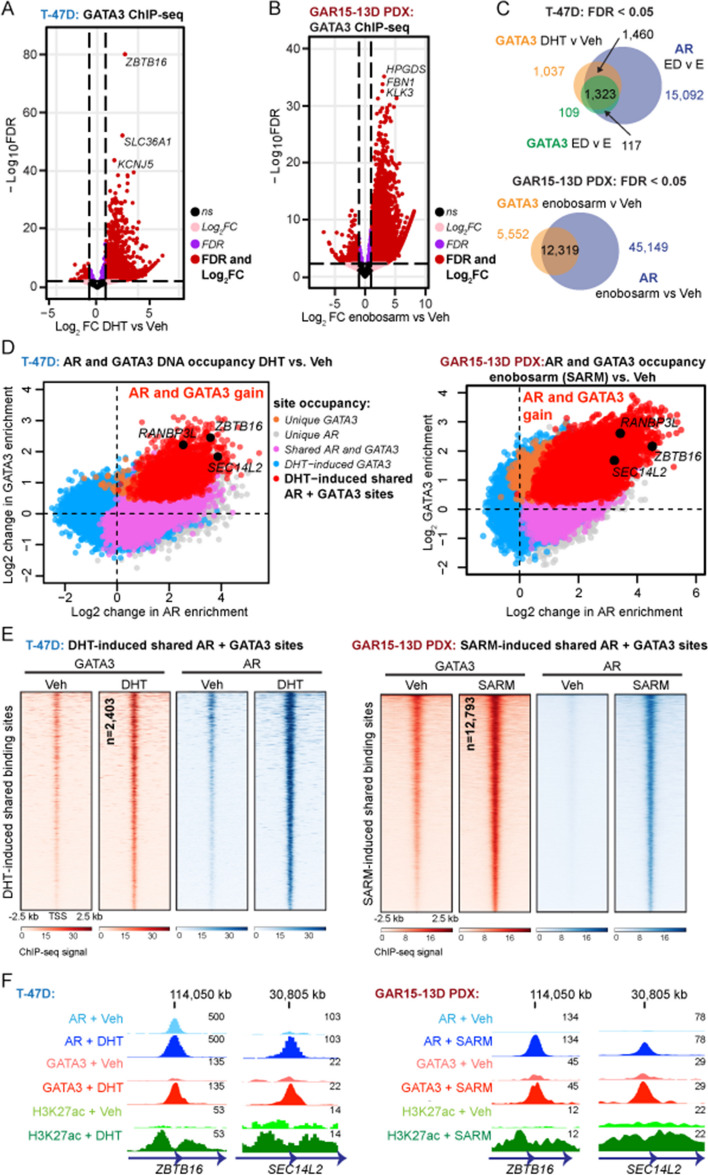
Activation of AR induces GATA3 chromatin binding at AR/GATA3 co-occupied loci in ER-positive breast cancer cells. Volcano plots reporting the FDR adjusted *p*-value and the log_2_ fold change (Log_2_ FC) of GATA3 chromatin binding events in (**A**) T-47D breast cancer cells treated with DHT vs Veh or (**B**) GAR15-13D PDX tumors from [8] collected 5 days after treatment with the selective androgen receptor modulator (SARM), enobosarm vs Veh. For visualization of differential binding patterns, the threshold for gain or loss in volcano plots is shown as FC > 1 (vertical lines) with an FDR cut-off of 5 × 10^−3^ (horizontal line). **C** Venn diagram showing overlap of enriched (FDR < 0.05, no fold-change threshold) AR and GATA3 binding sites after stimulation with DHT. **D** Two-factor log-ratio (M) plot displaying DHT (T-47D, left)- or SARM (GAR15-13D PDX, right)-induced changes in GATA3 and AR enrichment at consensus chromatin binding sites. Point color denotes treatment-induced changes in transcription factor occupancy (called peaks); GATA3 unique (orange, plotted in the rear), AR unique (grey) and Veh + DHT (or Veh + enobosarm; SARM) shared (pink), DHT- or SARM-induced GATA3 peaks that are not shared with AR (blue), and shared AR/GATA3 peaks that are significantly gained with DHT or SARM stimulation (red). Point co-ordinates are derived from the average enrichment score of three independent ChIP-seq replicates for each consensus binding site. Example binding sites near known AR target genes are highlighted. **E** Consensus GATA3, AR, and H3K27ac ChIP-seq data showing heatmaps demonstrative of a DHT- (T-47D, left) or enobosarm-induced (GAR15-13D, right) gain in GATA3 chromatin binding sites that are shared with AR. **F** Example genome browser images showing GATA3, AR, and H3K27ac ChIP-seq signals at loci associated with AR target genes *ZBTB16* (left panel) and *SEC14L2* (right panel) in T-47D cells. Data represents the average signal of three replicates

To determine whether sex hormone treatment alters the genome-wide GATA3 chromatin binding profile (cistrome), we first performed GATA3 ChIP-seq in the T-47D and ZR-75-1 ER+ cell lines treated 4 h with vehicle (Veh), androgen (DHT), estradiol (E2), or E2 + DHT after a period of hormone deprivation. ChIP-seq experiments were performed using three independent biological replicates representing consecutive passages of cells to generate consensus GATA3 cistromes that represent reproducible chromatin binding events under different treatment conditions (Additional file 2: Fig. S2A, B). While the majority of consensus GATA3 chromatin binding events were unaltered by hormone treatments, significant enrichment of a sub-set of GATA3 binding events was reproducibly observed following AR activation with DHT (T-47D: 3,928 gained; ZR-75-1: 5,144 gained; Fig. 2A; Additional file 2: Fig. S2C; Additional file 3). To investigate whether AR activation altered the GATA3 cistrome in a more clinically relevant context, we performed GATA3 ChIP-seq in bio-banked tumors from an AR+/GATA3+ patient-derived xenograft (PDX) model of ER+ breast cancer (GAR15-13D) treated for 5 days in vivo with a vehicle control or a selective AR modulator (SARM; enobosarm) that has AR agonist activity in ER+ breast cancer cells (Additional file 2: Fig. S2D-E) [8]. In support of our cell line data, AR activation with SARM induced significant enrichment of a subset of GATA3 binding sites in the PDX tumors (Fig. 2B). DHT- or SARM-induced GATA3 binding events significantly overlapped with treatment-induced AR binding events, representing 71% (T-47D; Fig. 2C), 72% (ZR-75-1; Additional file 2: Fig. S2F), and 80% (GAR15-13D PDX; Fig. 2C) of differentially bound GATA3 loci. Tracking genome-wide changes following treatment with an AR agonist revealed a gain of both factors at genomic loci associated with established AR target genes (Fig. 2D–E; Additional file 2: Fig. S2G-H; Additional file 4). Average read density analysis indicated that AR/GATA3 binding at shared loci was stronger than at genomic loci occupied by one factor alone (Additional file 2: Fig. S2I). Discriminative DNA motif analysis of DHT-induced AR/GATA3 binding sites identified common (AR/PR/GR) steroid receptor response elements and more specific AR response elements as the predominant motifs, while GATA3 binding sites unaffected by hormone treatment predominantly contained GATA motifs (Additional file 2: Fig. S2J-K). While binding of a small amount of both factors was evident in the absence of androgen hormone, DHT-induced enrichment of AR and GATA3 was concomitant with an increase in the signal for H3K27ac, a mark of active chromatin, at loci associated with known AR target genes, (Fig. 2F; Additional file 2: Fig. S2L). Therefore, an important functional consequence of androgen-induced interaction between AR and GATA3 is modulation of the GATA3 cistrome to facilitate regulation of AR target genes in ER+ breast cancer cells.

**Fig. 3:**
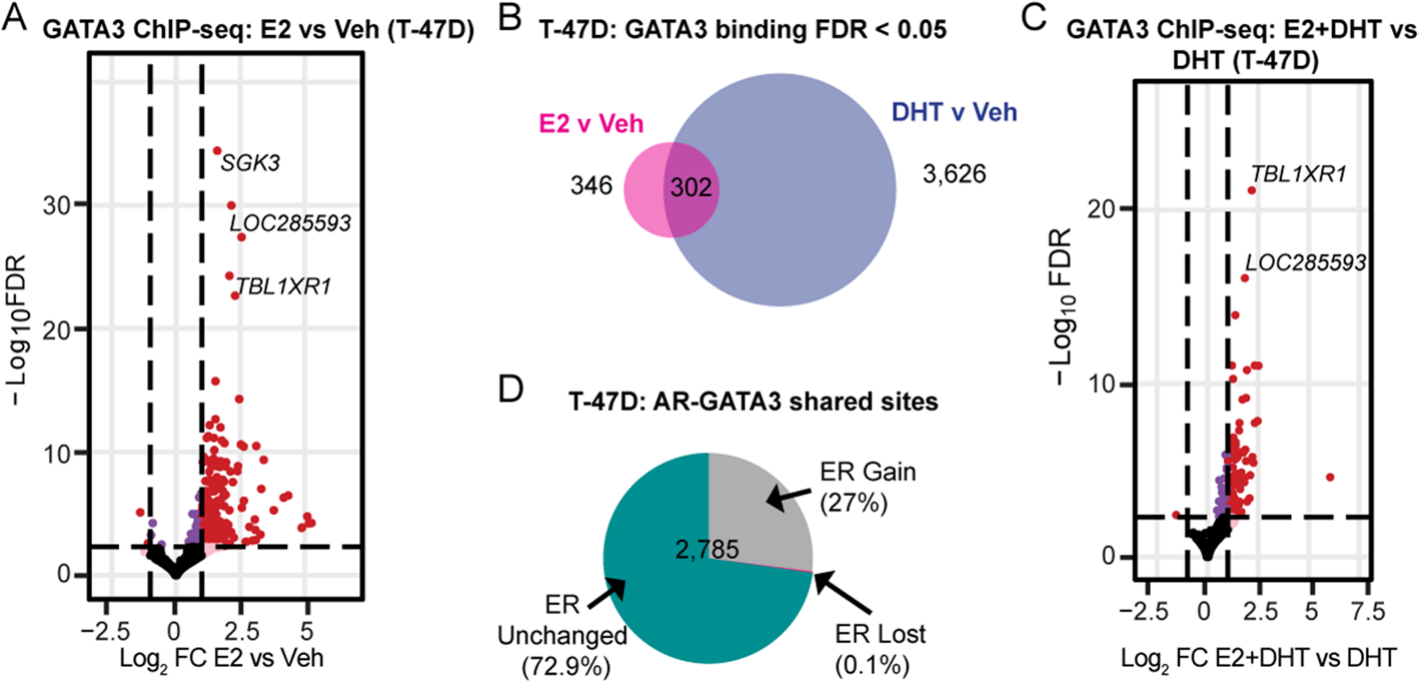
Hormone mediated changes in GATA3 chromatin binding in ER-positive breast cancer cells. **A** Volcano plot reporting the FDR adjusted *p*-value and the log2FC of GATA3 chromatin binding events in T-47D breast cancer cells treated with E2 vs Veh. Thresholds are set as in Fig. 2. **B** Venn diagram showing the overlap of significantly enriched (FDR < 0.05) GATA3 binding sites with E2 or DHT. **C** Volcano plot reporting the FDR adjusted *p*-value and the log_2_FC of GATA3 chromatin binding events with simultaneous hormone treatment (E2 + DHT) vs DHT alone. For visualization of differential binding patterns, the threshold for gain or loss in volcano plots is shown as in Fig. 2. **D** Differential ER binding (from [8], FDR < 0.05, no fold-change threshold) at AR and GATA3 shared sites

In the cell line models, treatment with E2 also enriched GATA3 chromatin binding, but at a much smaller number of genomic loci compared to DHT treatment (T-47D: 648 gained; ZR-75-1: 546 gained; Fig. 3A; Additional file 2: Fig. S3A; Additional file 3). Approximately half of these E2-stimulated GATA3 chromatin binding events overlapped with DHT-stimulated GATA3 binding events (Fig. 3B; Additional file 2: Fig. S3B). Hence, activation of AR was a markedly stronger driver of hormone-induced changes in the GATA3 cistrome than ER activation. In support of this, we used the GIGGLE platform to interrogate published ChIP-seq datasets for cistromes matching the DHT- or E2-induced GATA3 peaks identified herein. Top-ranking genomic loci significantly associated with DHT-induced GATA3 chromatin occupancy were dominated by AR, whereas the two factors with the highest GIGGLE score at E2-induced GATA3 sites were ER and AR (Additional file 2: Fig. S3C-D). GATA3 recruitment after simultaneous activation of ER and AR (E2 + DHT treatment) was similar to activation of AR alone, with no evidence of sex hormone receptor antagonism (Fig. 3C; Additional file 2: Fig. S3E; Additional file 3). Integration of the newly generated GATA3 cistromes with publicly available AR and ER cistromes previously generated by our group [8] revealed that the majority of E2 + DHT-stimulated AR/GATA3 binding events were not enriched for ER (Fig. 3D; Additional file 2: Fig. S3F), implying that the cooperative binding of AR and GATA3 at shared loci is mainly directed by AR.

**Fig. 4:**
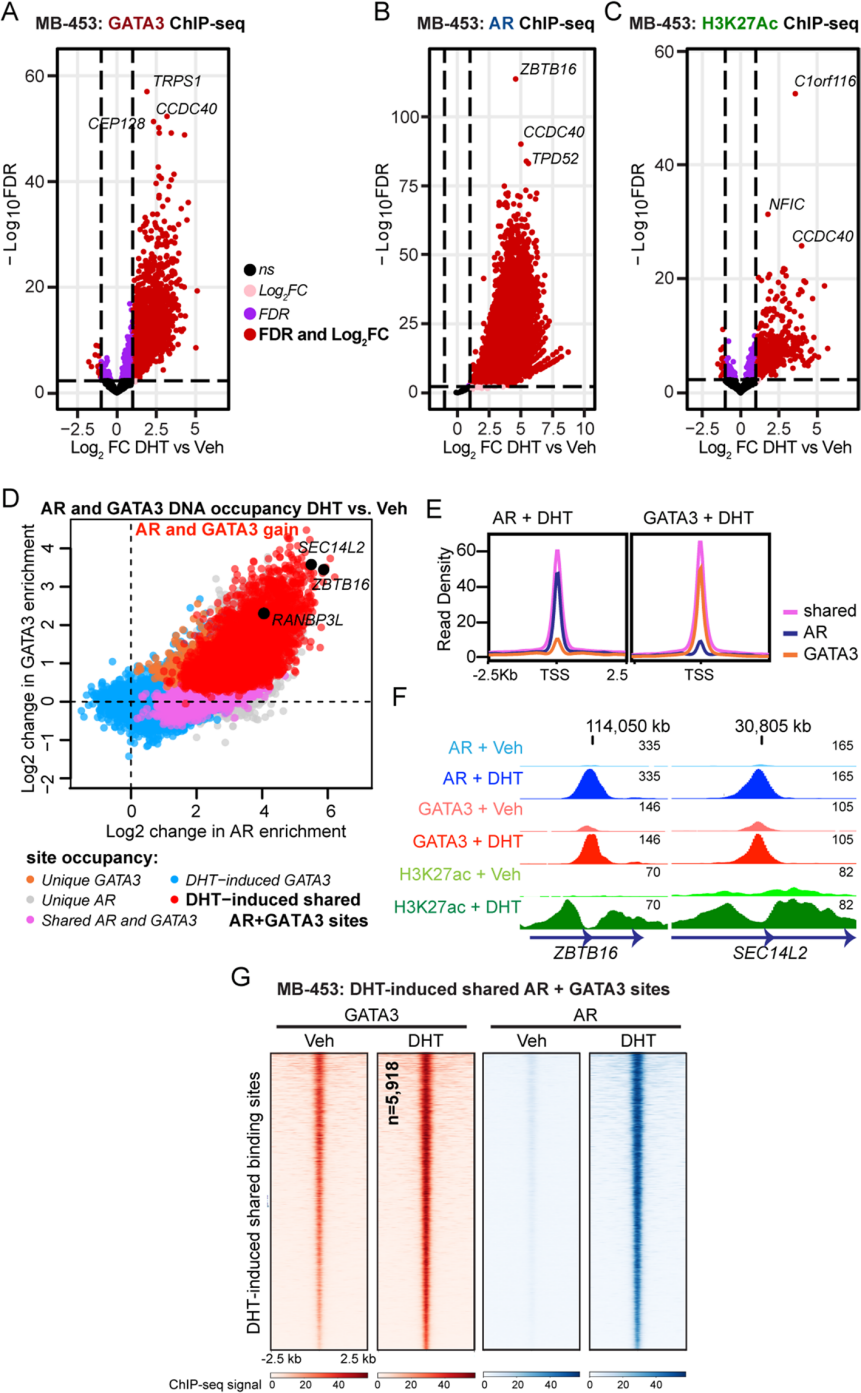
Activation of AR induces GATA3 chromatin binding at AR/GATA3 co-occupied loci in ER-negative breast cancer cells. Volcano plot reporting the FDR adjusted *p*-value and the log_2_FC of GATA3 (**A**), AR (**B**), and H3K27ac (**C**) chromatin binding events in MDA-MB-453 (MB-453) breast cancer cells treated with DHT vs Veh. For visualization of differential binding patterns, the threshold for gain or loss in volcano plots is shown as in Fig. 2. **D** Two-factor log-ratio (M) plot displaying DHT-induced changes in GATA3 and AR enrichment at chromatin binding sites in MDA-MB-453 cells. Point color denoting treatment-induced transcription factor occupancy as described in Fig. 3, where red dots indicate loci where occupancy of AR and GATA3 is enriched following treatment with DHT. **E** Average read density plots for AR (left) and GATA3 (right) chromatin occupancy proximal (< 100 kb) to unique or shared genes in MDA-MB-453 cells treated in vitro with DHT. **F** Example genome browser images showing GATA3, AR, and H3K27ac ChIP-seq signals at binding sites associated with AR target genes *ZBTB16* (left panel) and *SEC14L2* (right panel) in MDA-MB-453 cells. Data represents the average signal of three replicates. **G** Consensus GATA3 and AR ChIP-seq data from **D** showing heatmaps demonstrative of a DHT-induced gain in GATA3 chromatin binding sites that are shared with AR

To determine if a similar phenomenon occurs in the context of ER- breast cancer, we performed AR, GATA3, and H3K27ac ChIP-seq in MDA-MB-453 and MFM-223 breast cancer cell lines treated for 4 h with DHT or a vehicle control after a period of hormone deprivation. Three independent biological replicates representing consecutive passages of cells were used to generate consensus AR, GATA3, and H3K27ac cistromes (Additional file 2: Fig. S4A, B). As observed in ER+ models (Fig. 2; Additional file 2: Fig. S2), treatment with DHT significantly induced a sub-set of GATA3 binding sites in MDA-MB-453 (7,426; 25% of consensus peaks) (Fig. 4A; Additional file 3) and MFM-223 (8,734; 15% of consensus peaks) cells (Additional file 2: Fig. S4C; Additional file 3), concomitant with a striking global increase in AR binding (Fig. 4B; Additional file 2: Fig. S4D; Additional file 3). Changes in H3K27ac enrichment were also observed (Fig. 4C; Additional file 2: Fig. S4E; Additional file 3). The degree of AR and GATA3 co-occupancy following treatment with DHT was also high in the ER- breast cancer cell lines, representing 80% of GATA3 binding sites in MDA-MB-453 and 60% in MFM-223 cells (Fig. 4D; Additional file 2: Fig. S5 A-C; Additional file 4). These co-occupied loci demonstrated stronger read density signals than loci occupied by either factor alone (Fig. 4E; Additional file 2: Fig. S5D) and were highly enriched for AR DNA binding motifs (Additional file 2: Fig. S5E). Likewise, genomic loci corresponding to androgen-induced AR recruitment and GATA3 enrichment were located near AR target genes that were transcriptionally activated (Fig. 4F, G; Additional file 2: Fig. S5F, G). While there is evidence of some model-specific binding patterns, the AR/GATA3 binding sites shared among all models were stronger than model-specific AR or GATA3 binding events (Additional file 2: Fig. S5H, orange line). Similarly, the majority of DHT-regulated genes associated with AR/GATA3 regulatory loci were not model specific (Additional file 2: Fig. S5I, indicated in purple; Additional file 5), suggesting these sites have biological significance. This data indicates that the androgen-induced interaction between AR and GATA3 has similar functional consequences in ER+ and ER- breast cancer models.

### GATA3 is an AR co-regulator in breast cancer cells

**Fig. 5:**
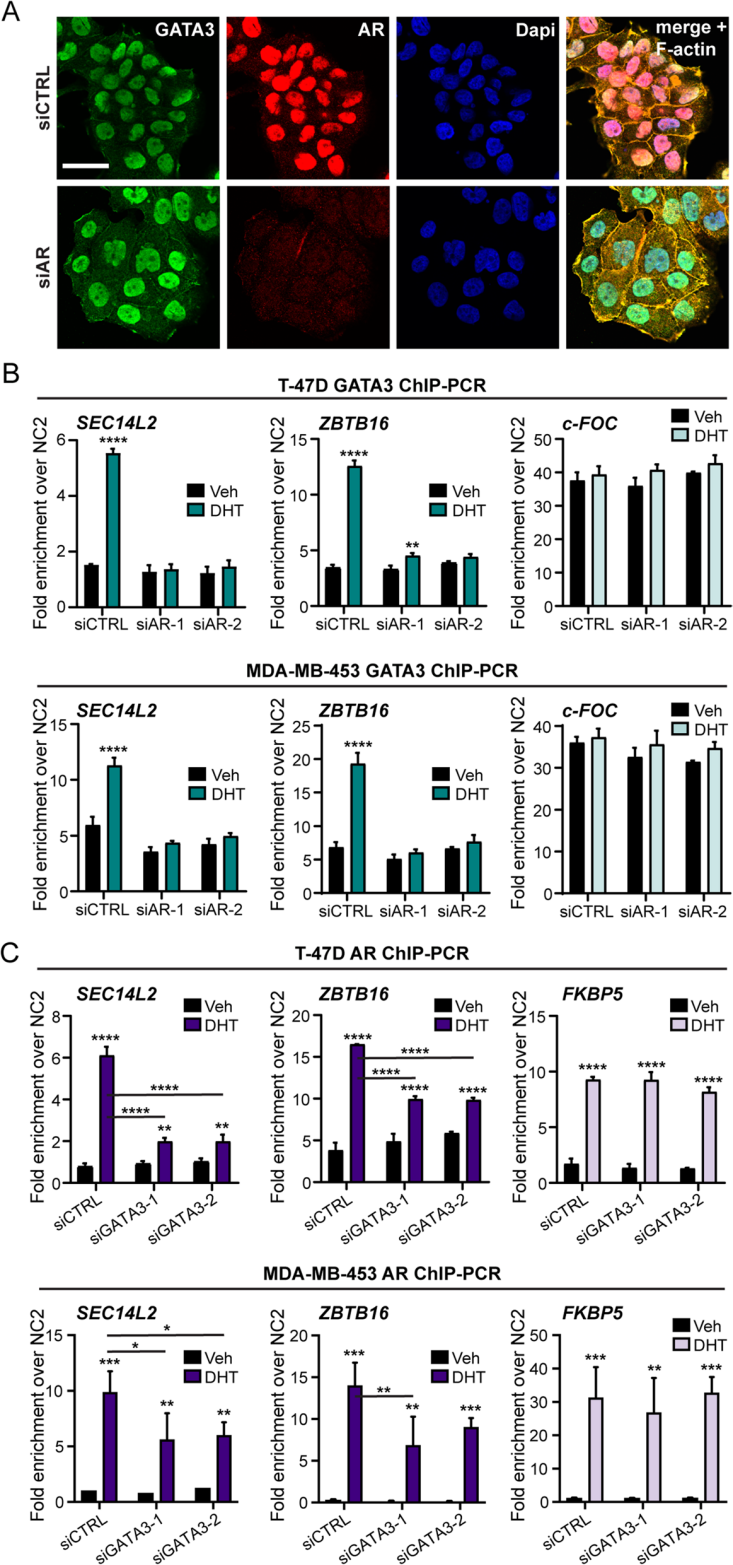
GATA3 promotes, but is not essential for, expression of shared AR and GATA3 target genes. **A** Representative immunofluorescence images showing AR silencing in T-47D cells. Scale bar = 30 μm. **B** GATA3 ChIP-PCR at AR/GATA3 co-occupied loci associated with DHT-regulated AR target genes following AR silencing by two different siRNAs in T-47D cells (upper panel) and MDA-MB-453 cells (lower panel), treated in vitro with Veh (EtOH) or DHT (10 nM). Data was analyzed by a two-way ANOVA. Post hoc analyses were performed using Tukey’s multiple comparisons test, where *SEC14L2*, *P* < 0.0001 for Veh versus DHT in siControl only; *ZBTB16*, *P* < 0.0001 and *P* < 0.01 for Veh versus DHT in siControl and siAR-1, respectively. Analysis of GATA3 chromatin binding at a locus associated with the *C-FOC* gene was included as a negative control for androgen-unresponsive GATA3 binding. **C** AR ChIP-PCR at AR/GATA3 co-occupied loci associated with DHT-regulated genes following GATA3 knockdown in T-47D cells (upper panel) and MDA-MB-453 cells (lower panel), treated in vitro with Veh (EtOH) or DHT (10 nM). Data was analyzed by a two-way ANOVA. Post hoc analyses were performed using Tukey’s multiple comparisons test, where *SEC14L2*, *P* < 0.0001, and *P* < 0.01 for Veh versus DHT in siControl and siGATA3, respectively, and *P* < 0.0001 for siControl + DHT versus siAR + DHT; *ZBTB16*, *P* < 0.0001 for Veh versus DHT in siControl and siGATA3, and *P* < 0.0001 for siControl + DHT versus siGATA3 + DHT. Analysis of AR chromatin binding at a locus associated with the *FKBP5* gene was included as a control for GATA3-independent AR chromatin binding. Data is shown as mean ± SEM of 3 biological replicates from consecutive passages of cells

To investigate whether DHT-induced enrichment of GATA3 at loci associated with AR target genes (Figs. 2, 3, and 4) is dependent on recruitment of AR, we performed GATA3 ChIP-PCR in the presence or absence of siRNA-mediated AR knock-down in T-47D (as an ER+ model) and MDA-MB-453 (as an ER- model) cells. The efficacy of AR knock-down was confirmed via dual label IF (Fig. 5A; Additional file 2: Fig. S6A) and Western blotting (Additional file 2: Fig. S6B). AR knockdown did not significantly alter levels of GATA3 protein (Fig. 5A; Additional file 2: Fig. S6A, B). However, silencing AR abolished DHT-induced enrichment of GATA3 at loci associated with *SEC14L2* and *ZBTB16* genes in both cell line models (Fig. 5B), verifying that GATA3 enrichment required AR. Reduced GATA3 binding following AR knockdown resulted in significantly reduced transcript expression of *SEC14L2* in both models and reduced *ZBTB16* only in the ER+ model (Additional file 2: Fig. S6C). Conversely, GATA3 enrichment was not affected by AR knockdown at a locus associated with the *c-FOC* gene that was not influenced by AR activation in any of the cell line models (Fig. 5B). Next, we performed the reciprocal silencing experiment to show that knockdown of GATA3 did not significantly change AR protein levels in T-47D and MDA-MB-453 cells (Additional file 2: Fig. S6D) but did significantly reduce DHT-mediated recruitment of AR to representative AR target gene loci (Fig. 5C). As expected, reduced AR binding following GATA3 knockdown reduced but did not abolish transcriptional activity (Additional file 2: Fig. S6E), but GATA3 knock-down had no effect on DHT-induced AR recruitment at a locus associated with the *FKBP5* AR target gene that is not co-occupied by GATA3 (Fig. 5C). Taken together, these results indicate that GATA3 facilitates but is not essential for AR chromatin binding and implicate GATA3 as an AR co-activator in breast cancer cells.

Since GATA3 is an essential gene in ER+ breast cancer cells [33, 34], we could not use silencing of GATA3 to explore the functional impact of DHT-induced AR-GATA3 interactions on the tumor suppressor role of AR in ER+ breast cancer cells. Therefore, we assessed interaction between the two proteins in T-47D cells following treatment with a growth inhibitory dose of the selective AR modulator (SARM), enobosarm (100 nM) and compared that to a dose of enobosarm (10 nM) that does not inhibit proliferation [8]. Only the growth inhibitory dose of enobosarm significantly induced cellular AR-GATA3 interactions and did so to an extent approximately equal to that induced by a growth inhibitory dose of DHT (10 nM) (Additional file 2: Fig. SS6F). This data indicates that AR-GATA3 interactions are required for AR-mediated growth inhibition of ER+ breast cancer cells. In the context of ER- breast cancer, AR activation inhibits proliferation of MFM-223 cells [9, 35], but has variable proliferative effects on MDA-MB-453 cells [36, 37]. We found that silencing GATA3 nearly abolished proliferative capacity of both ER- models, indicating it is an essential gene in ER- as well as ER+ breast cancer cells (Additional file 2: Fig. S6G), which aligns with their GATA3 dependency scores in the Cancer Dependency Map portal (www.depmap.org) (Additional file 2: Fig. S6H). However, treatment with DHT partially rescued proliferative capacity of MDA-MB-453 cells in the absence of GATA3, whereas MFM-223 cells remained non-proliferative in the absence of GATA3 regardless of DHT treatment (Additional file 2: Fig. S6G). The MDA-MB-453 data suggests that DHT-induced interactions between AR and GATA3 restrain the ability of AR to stimulate proliferation, while not conferring the degree of growth inhibition observed in ER+ models or in the MFM-223 model. Notably, the MDA-MB-453 cell line exhibited markedly fewer DHT-induced AR/GATA3 interactions compared to the other three cell lines despite having comparable or higher expression of AR and GATA3 (Fig. 1E, Additional file 2: Fig. S1A-F). Collectively, these data indicate that GATA3 is a critical co-factor for AR action in ER+ and ER- breast cancer and facilitates AR-mediated tumor suppressor activity.

### Androgen-induced AR-GATA3 binding events are associated with a gene program controlling development and differentiation of the mammary epithelium

**Fig. 6:**
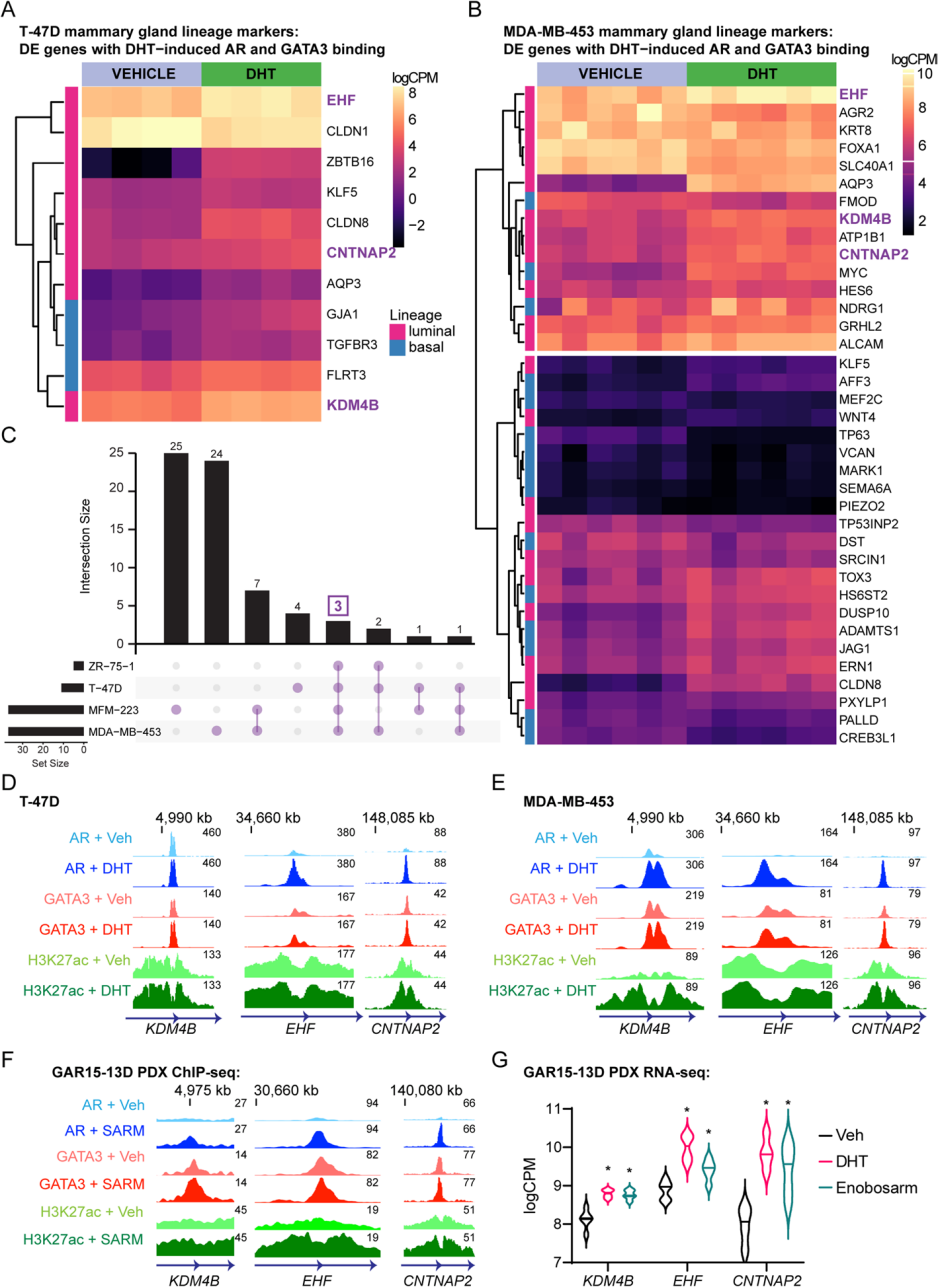
AR agonist-induced shared AR and GATA3 target genes are implicated in development and differentiation of mammary epithelium. **A** RNA-seq heatmap of differentially expressed (FDR < 0.05) mammary lineage marker genes associated with a DHT-induced shared AR and GATA3 binding event in T-47D and **B** MDA-MB-453 cells. Luminal marker genes are denoted by pink squares (*y*-axis) and basal marker genes are denoted by blue squares. Gene expression is represented as the log counts per million (logCPM) so as to observe expression differences between genes (rows). Genes common to all ER+ (T-47D, ZR-75-1) and ER- (MDA-MB-453, MFM-223) cell lines, as shown in **C**, are indicated in purple text. **D** Example genome browser images showing averaged GATA3, AR and H3K27ac ChIP-seq signals at binding sites associated with *KDM4B* (left panel), *EHF* (middle panel), and *CNTNAP2* (right panel) in T-47D, **E** MDA-MB-453 cells, and **F** GAR15-13D ER+ PDX. Data represents the consensus signal of three independent replicates. **G ***KDM4B*, *EHF*, and *CBTBAP2* transcript expression (logCPM) in GAR15-13D ER+ PDX tumors treated with DHT or enobosarm from [8]. Data was analyzed by ordinary two-way ANOVA followed by Dunnett’s multiple comparisons test comparing DHT or enobosarm treatment to vehicle (Veh) for each gene, where *P* < 0.05 versus Veh

To probe the potential biological significance of DHT-induced AR/GATA3 chromatin binding events, we integrated ChIP-seq data (Figs. 2 and 3; Additional file 2: Figs S2-3) with RNA-seq data we generated from ER+ and ER- breast cancer cell lines stimulated with DHT to identify androgen-regulated AR-GATA3 binding sites associated with differentially expressed genes. Six replicates representing sequential passages of cells generated high-quality transcriptomes for MDA-MB-453 and MFM-223 cells (Additional file 6). The ER+ cell line transcriptomic data was generated by our group previously ([8], GSE123770). Most of the genes changing significantly in response to androgen stimulation were induced (T-47D: 85%, 55 genes; ZR-75-1: 85%, 100 genes; MDA-MB-453: 63%, 387 genes; MFM-223: 50%, 385 genes; Additional file 5). Development, morphogenesis, and differentiation pathways were enriched in AR-GATA3 co-occupied sites associated with differentially expressed genes (Additional file 7). Since GATA3 is a master regulator of the luminal phenotype in mammary epithelial cells [28, 30] and activation of AR has been implicated in promotion of a basal-to-luminal epithelial cell transition in the mouse mammary gland [38], we profiled the DHT-induced AR/GATA3 binding sites for association with genes relating to mammary epithelial cell identity and differentiation, selecting those genes demonstrating significant changes in expression with DHT treatment in the RNA-seq datasets (Additional file 6). As expected, we observed a variable expression pattern of luminal and basal lineage genes among the cell line models with hierarchical clustering driven by high vs low gene expression (Fig. 6A, B; Additional file 2: Fig. S7A, B). Nonetheless, three genes (*CNTNAP2*, *EHF*, *KDM4B*; Fig. 6C; Additional file 8) were significantly upregulated by DHT in all four models of breast cancer and displayed enrichment of AR, GATA3, and H3K27ac ChIP-seq signals at associated genomic loci (Fig. 6D, E; Additional file 2: Fig. S7C). In support of this, we found evidence of SARM-induced regulation of *KDM4B, EHF* and *CNTNAP2* in GAR15-13D ER+ PDX tumors (Fig. 6F–G). These genes were of particular interest because EHF is an ETS transcription factor critical for establishing epithelial identity in many tissues [39] and is part of a transcription factor network that distinguishes basal from luminal progenitor cells in the human breast epithelium [40], while KDM4B is a histone demethylase required for expression of ER that also facilitates transcription of ER responsive genes during mammary gland development [41–43]. Although there is no evidence to implicate CNTNAP2 in breast cancer or mammary gland biology, it is thought to play a role in axonal differentiation and guidance [44, 45]. We validated that KDM4B protein expression was increased by DHT in ER+ and ER- breast cancer cell lines, and this increase was abrogated by AR knockdown (Additional file 2: Fig. S7D). Consistent with observations at known AR target genes (Fig. 4), silencing AR prevented DHT-induced enrichment of GATA3 binding and prevented or reduced transactivation of the *KDM4B* and *EHF* genes (Additional file 2: Fig. S8A, B). Likewise, silencing GATA3 negatively impacted but did not eradicate DHT-induced AR binding and gene transactivation (Additional file 2: Fig. S8C, D). In clinical datasets, high expression of KDM4B (Additional file 2: Fig. S8E), but not EHF (data not shown), was associated with better patient outcomes, although EHF is one of 142 genes comprising a gene signature of AR activity that is prognostic in ER+ breast cancer [8]. These data identify *KDM4B* and *EHF* as novel AR target genes in breast cancer cells and show that GATA3 co-regulates AR signaling in transcriptional regulation of these and likely other genes that promote a luminal epithelial phenotype in ER+ and ER- breast cancer contexts. Collectively, our findings revealed that in ER+ and ER- breast cancer cells, AR activation induces an interaction with GATA3 that results in creation of AR-dependent GATA3 chromatin binding events that are predominantly associated with AR target genes, where GATA3 acts as an AR co-factor to regulate a gene program that promotes a luminal epithelial phenotype and can facilitate its role as a tumor suppressor.

## Discussion

The AR is expressed in all major subtypes of breast cancer but mechanistic understanding of AR as a transcription factor in is limited. Since transcription factors interact with numerous other nuclear proteins to regulate transcription, we performed an unbiased proteomic analysis of molecularly diverse breast cancer cell lines to characterize their AR-dependent multi-subunit protein complexes. This approach revealed the DNA binding, chromatin remodeling factor GATA3 as a fundamental member of the breast cancer AR interactome regardless of disease context. Interaction of AR with GATA3 has not been previously reported in any tissue or cellular context. We confirmed the AR-GATA3 interaction in cell line models, showed the interaction increased upon treatment with androgen, and detected AR-GATA3 interactions in primary clinical breast cancers as well as non-malignant human breast tissues. Hormone-induced interactions between AR and GATA3 created new cis-regulatory chromatin binding events that were associated with transcriptional up-regulation of genes with fundamental roles in epithelial cell differentiation in breast and other tissues, indicating biological relevance. Consistent with promotion of cellular differentiation, we provide evidence that the AR-GATA3 interaction facilitates the tumor suppressor function of AR. While expression of ER is a defining feature of breast cancers with the most differentiated luminal phenotype, our data supports a functional role for AR signaling in the promotion of luminal differentiation in breast cancers regardless of ER status, which could mechanistically explain the positive association between AR expression and tumor indolence, luminal phenotypic features as well as prolonged disease-free survival in multiple subtypes of this disease [8, 46–49].

While GATA3 is an established master regulator of the luminal lineage of epithelial cell differentiation in the mouse mammary gland [28–31], a role for AR in this process has not been established. Our discovery that AR and GATA3 cooperate to promote expression of luminal genes is consistent with molecular pathology studies reporting a strong positive correlation between AR and GATA3 expression in breast cancers, even stronger than the correlation between ER and GATA3 [50, 51]. Cooperative promotion of luminal differentiation by AR and GATA3 also mechanistically unites independent studies linking expression of one or the other factor with a more differentiated, less proliferative phenotype in breast cancer cells regardless of molecular sub-type [5, 38, 49, 52–56]. Our new mechanistic insight is consistent with a previous study showing that stimulation of AR in female mice promotes a basal-to-luminal cell phenotypic transition in the normal mammary epithelium and identification of AR positive cells with a hybrid luminal/basal phenotype in human breast tissues [38]. Although the latter study did not examine expression of GATA3, a recent single cell spatial analysis of human breast tissues at the transcriptomic and proteomic level identified a basal/luminal hybrid cell that expressed low levels of AR and GATA3 while also showing a concordance of expression of these factors in diverse subsets of epithelial cells representing multiple subsequent stages of luminal differentiation [57]. All breast cancers arise from epithelial cells committed to the luminal lineage, with different subtypes of disease either arising from progenitor cells at various stages of differentiation [58, 59] or having phenotypes that reflect progressive stages of reversion from differentiated to an undifferentiated mammary epithelial cells during malignant transformation [60, 61]. Our data suggests that AR partners with GATA3 to regulate luminal epithelial cell differentiation from early stages, represented by AR+ER- breast cancer cell lines, through to more mature stages, represented by AR+ER+ breast cancer cell lines, explaining why AR is such a prevalent sex hormone receptor in this disease.

To our knowledge, this is the first report of an AR-GATA3 interaction in any tissue or cell type. Among body tissues or pathologies, AR signaling is most comprehensively investigated in prostate cancer, where it is the key oncogenic driver. Although another member of the GATA family, GATA2, regulates AR signaling in prostate cancer cells [62, 63], proteomic studies have not identified GATA2 as being part of the AR interactome in prostate cancer cell lines or primary tissues [25, 64]. Moreover, GATA2 expression is associated with more, rather than less, aggressive prostate cancer, with overexpression increasing metastatic capacity [65], while GATA3 is associated with less aggressive breast cancer and overexpression prevents metastasis [53]. Hence, although GATA2 and GATA3 share about 55% structural homology, they retain distinct functions [29]. The interaction between AR and GATA3 we discovered herein in breast cancer may in part explain why AR has distinct roles in breast compared to prostate tissues. Indeed, AR has a tumor suppressor role in ER+ breast cancers [8], which may be sustained in some contexts of ER- breast cancer [9]. Data presented herein support the concept that androgen-induced AR/GATA3 interactions facilitate the tumor suppressive function of AR in ER+ and ER- breast cancer contexts. Mechanistically, activation of AR in ER+ breast cancer cells sequestered essential transcriptional co-activators (p300, SRC-3) away from ER-regulated cell cycle genes to up-regulate AR target genes, including known tumor suppressors [8]. In the latter study, ER was recruited to some of the AR target genes in an AR-dependent manner, indicating co-operative activity in the promotion of differentiation. Since AR and ER do not directly interact, an intermediary factor was likely involved. Herein, we provide evidence that GATA3 may form a molecular bridge between AR and ER at a sub-set of growth regulatory genes since recruitment of GATA3 at these genomic loci was dependent on AR. However, the AR-GATA3 interaction does not require ER since AR also recruited GATA3 to loci not occupied by ER, and AR-GATA3 interactions were a feature of ER- breast cancer cells. Therefore, in the context of ER+ breast cancer, the interaction between AR and GATA3 likely plays a role in reprogramming ER signaling to inhibit proliferation and promote terminal differentiation. In the context of ER- breast cancer, the expression of AR and its interaction with GATA3 likely promotes a more differentiated luminal phenotype, explaining why AR-ER- breast cancers represent the most aggressive, undifferentiated breast cancer subtype.

In addition to GATA3, two other DNA binding transcription factors, JUNB and ERF, were identified as AR interacting proteins in all breast cancer cell line models. Both factors have been identified as AR interacting proteins in LNCaP-derived cell line models of prostate cancer [25]. JUNB is a subunit of the AP-1 transcription factor complex and is reported to have tumor suppressive functions in prostate cancer by preventing progression of low-grade prostate intraepithelial neoplasia (PIN) to high-grade PIN lesions [66]. The Ets2 repressor factor, ERF, also has tumor suppressor activity in the prostate [67]. Consistent with being an AR interacting protein that confers tumor suppressor activity, ERF chromatin occupancy overlapped with AR binding sites by 28% in normal prostate organoids but not in a prostate adenocarcinoma cell line [67]. These studies indicate that JUNB and ERF may be key determinants of normal AR signaling in the prostate gland but not determinants of oncogenic AR signaling in prostate cancer. In breast cancer, JUNB and ERF are transcriptional repressors downregulated by ER signaling, a feature that facilitates ER-induced transcription of proliferation genes [68]. Furthermore, in vitro evidence implicates ERF in transcriptional repression of the *c-Myc* oncogene in ER+ breast cancer cells [69]. Hence, androgen-induced interactions between AR and JUNB or ERF may confer growth inhibitory actions that are conserved across breast and prostate cancer contexts, but this requires future investigation.

Interestingly, FOXA1, another DNA binding and chromatin remodeling factor important in steroid receptor signaling [70], was only detected as a high confidence AR interacting protein in the MDA-MB-453 cell line, despite comparable FOXA1 expression in all models (Additional file 2: Fig. S8F; Additional file 1). Like GATA3, FOXA1 is a known ER interacting protein in breast cancer, but unlike GATA3, FOXA1 is essential for ER to bind DNA [71]. FOXA1 is also an integral part of the AR interactome in prostate cancers [25, 64, 72] but is not required for AR to bind DNA [73]. We have previously shown that the AR and FOXA1 cistromes demonstrate an extraordinary overlap in MDA-MB-453 cells and that FOXA1 is required for AR to regulate genes that define the molecular apocrine breast cancer phenotype [20, 71]. In addition, FOXA1 overexpression increased the proliferative capacity of AR signaling in MDA-MB-453 breast and LNCaP prostate cancer cell lines [20]. Our AR RIME data is consistent with these MDA-MB-453 studies but does not rule out an interaction between AR and FOXA1 in the other breast cancer models. Rather, it suggests that the interaction between AR and FOXA1 is not as prevalent or dominant in the other cell lines or is more dependent on hormone stimulation. Given that of all the breast cancer models investigated, the MDA-MB-453 cell line is the only one in which AR signaling can promote rather than inhibit proliferation [23, 36, 37], increased or preferential interaction with FOXA1 over GATA3 or other factors may underpin this distinct biology. Furthermore, we provide the first evidence that silencing GATA3 in MDA-MB-453 cells enables AR to stimulate proliferation, supporting the concept that AR/GATA3 interactions are tumor suppressive even in this context. We have shown via silencing and overexpression approaches that FOXA1 is critical for AR to have oncogenic activity in MDA-MB-453 cells [20, 71, 72], but whether knockdown of FOXA1 would facilitate AR/GATA3 interactions and AR-mediated tumor suppressor activity in this or any other breast cancer context is unknown. Hence, the dynamics of AR signaling in the context of GATA3 and FOXA1 warrants further investigation. In addition, our datasets provide ample candidate AR interacting proteins in different contexts of breast cancer that may play a role in determining context-specific AR signaling activity.

KDM4B was identified as an androgen-induced gene associated with the AR-GATA3 interaction in all breast cancer cell lines regardless of ER status. KDM4B is a histone demethylase also known as JMJD2B with some established roles in mammary epithelial cells [41, 74]. Deletion of KDM4B in mouse mammary epithelial cells causes delayed mammary gland development in female mice [41] and is required for expression of ER and FOXA1 [43]. In breast cancer, KDM4B has been implicated as an estrogen-regulated gene that acts as an ER co-factor in the promotion of luminal differentiation [41, 43, 74]. Using knockdown and overexpression approaches, a recent study has shown that KDM4B inhibits proliferation and metastasis in ER+ and ER- breast cancer cells, supporting a tumor suppressive role for KDM4B in both contexts [75]. Consistent with our results, the latter study also reported an association between higher levels of KDM4B expression and better breast cancer outcomes. Herein, we further demonstrate that KDM4B is an AR/GATA3 target gene in ER- and ER+ breast cancers, supporting the concept of AR co-operating with ER signaling to promote differentiation in ER+ disease and independently promoting differentiation in ER- disease. We propose that AR/GATA3-mediated induction of KDM4B facilitates maintenance of a luminal epithelial transcriptome in breast cancer cells and forms part of a tumor suppressive nexus regardless of ER status. Further investigation of the cistromic interplay between KDM4B, GATA3 and AR in the breast is required to definitively identify how KDM4B contributes to AR-mediated luminal programs and tumor suppression in the breast.

We also identified the epithelial cell specific Ets family transcription factor EHF as a novel AR-GATA3 target gene in breast cancer cells. Developmentally, EHF has been shown to regulate lineage-specific enhancer-promoter interactions associated with terminal differentiation of epithelial cells [76]. In the breast, EHF is a key transcription factor that distinguishes luminal progenitor cells from basal epithelial cells in the mammary epithelial lineage [40], consistent with a role in promoting epithelial differentiation. Indeed, EHF is only expressed in differentiated luminal epithelial cells of the human breast and its expression is significantly decreased or lost in pre-cancerous breast lesions [77, 78]. Using overexpression and knockdown approaches, recent studies have shown that EHF has a tumor suppressor role in triple negative breast cancer cells via inhibition of epithelial-mesenchymal transition (EMT) and metastasis while sensitizing cells to chemotherapy and inducing apoptosis [79, 80]. Likewise, studies have found EHF to be an important tumor suppressor in the prostate and pancreas via promotion of differentiation and inhibition of EMT, which impedes metastasis and is rescued by re-expression of EHF [81–83]. Similar findings have been observed for colorectal and head and neck squamous cell carcinomas [79, 84]. Hence, AR-GATA3 mediated up-regulation of EHF in breast cancer cells may play a key role in mediating the tumor suppressor activity associated with AR signaling.

## Conclusions

In conclusion, we have revealed a novel interaction between AR and GATA3 transcription factors in breast cancer cells that was functionally linked to promotion of a more differentiated phenotype. This interaction may play a role in mediating the tumor suppressor activity of AR in breast tissues regardless of ER status.

## Methods

### Cell culture

The ZR-75-1 (ATCC #CRL-1500 pg, RRID: CVCL_0588), T-47D (HTB-133, RRID: CVCL_0553), and MDA-MB-453 (HTB-131, RRID: CVCL_0418) breast cancer cell lines were obtained from American Type Cell Culture Collection (ATCC; Manassas, VA, USA), and the MFM-223 cell line was obtained from the DMSZ-German Collection of Microorganisms and Cell Cultures (Braunschweig, Germany, RRID: CVCL_1408). All cell lines were routinely tested for mycoplasma infection and authenticity confirmed by short tandem repeat profiling (Cell Bank Australia). ZR-75-1 and T-47D cells were maintained in RPMI-1640 medium (Invitrogen) containing 10% Fetal Bovine Serum (FBS) and 2 nM L-Glutamine (Sigma). MFM-223 cells were cultured in EMEM (Sigma) containing 10% FBS, 2 nM L-Glutamine (Sigma), 1 × Non-essential Amino Acids (Sigma), and 1 × Insulin–Transferrin–Sodium Selenite (Sigma). MDA-MB-453 cells were maintained in DMEM (Sigma) medium containing 10% FBS, 2 nM L-Glutamine (Sigma) and 1 × Sodium Pyruvate (Sigma). All lines were incubated at 37 °C and 5% CO_2_.

### Rapid immunoprecipitation mass spectrometry of endogenous proteins (RIME)

The RIME technique was performed as described previously [13]. Briefly, cells (MDA-MB-453, MFM-223, ZR-75-1, and T-47D) were seeded at approximately 80% confluence in their appropriate growth medium and cultured for 48 h, then cross-linked in warm medium containing 1% formaldehyde for 7 min, quenched with 0.2 M Glycine, chromatin isolated and sonicated then subjected to immunoprecipitation using magnetic beads pre-bound with 10 μg of AR antibody (Santa Cruz Biotechnology Cat# sc-816, RRID:AB_1563391). An on-bead peptide digestion was performed, and a 2–5 μL aliquot of diluted peptide mixture was analyzed by Nano-LC–MS/MS. Peptides were identified using Proteome Discoverer (v1.4) (RRID:SCR_014477) and Mascot (RRID:SCR_014322) and/or SEQUEST (ProteinProphet (RRID:SCR_000286)) search engines as described in [13]. Only those interacting proteins that were identified in 3 of 3 independent biological replicates were considered for further analysis. Additional filtering was achieved by excluding non-specific interactions that appeared in > 1 of the 3 replicates of matching IgG negative controls.

### Proximity ligation assay (PLA)

ZR-75-1, T-47D, MFM-223, and MDA-MB-453 breast cancer cells were seeded on top of sterilized coverslips in 6-well culture plates in phenol red-free media containing dextran coated charcoal-stripped serum (DCC) for hormone starvation. Media was refreshed after 48 h. On the following day, cells were treated with ethanol (Vehicle control) or 10 nM DHT for 4 h before fixation with 4% paraformaldehyde for 10 min at room temperature. Cells were then permeabilized with 0.05% Triton X-100 for an hour at room temperature and stained with AR (LSBio (LifeSpan) Cat# LS-B3326, RRID:AB_2060169) and GATA3 (Thermo Fisher Scientific Cat# MA1-028, RRID:AB_2536713) antibodies diluted in 10% Donkey serum (in PBS) overnight at 4 °C. Antibody dilutions are listed in Additional file 9. PLA probes were mixed and diluted 1:5 in Duolink In Situ Antibody diluent for 1 h at 37 °C in a humid chamber. Ligation and amplification steps were conducted for 30 min and 100 min, respectively, at 37 °C according to the manufacturer’s instructions (Duolink® PLA kit; Sigma-Aldrich). Cell nuclei were stained with DAPI (Thermo Fisher Scientific Cat# D1306, RRID: RRID:AB_2629482) before mounting with Duolink In Situ Mounting Medium. For tissue PLA, sections were cut to 4 μM thickness and dewaxed in xylene followed by rehydrating in ethanol. Antigen retrieval decloaking was performed using citrate solution, pH 6.5. Slides were blocked in 10% donkey serum in PBS for 30 min at room temperature and incubated with 200 μL of diluted primary antibodies (AR (LSBio (LifeSpan) Cat# LS-B3326, RRID:AB_2060169) and GATA3 (Thermo Fisher Scientific Cat# MA1-028, RRID:AB_2536713) overnight at 4 °C. Antibody dilutions are listed in Additional file 9. Ligation and amplification of PLA probes was performed as above, except for a 1 h ligation.

Images were sequentially acquired on an Olympus Confocal Laser Scanning Microscope Fluoview FV3000 (RRID:SCR_017015). Nine sections that were systematically sampled at × 60 magnification were selected from 5 random spots for each slide as technical replicates. Level adjustments were applied across entire images. Quantification of the total number of the cells and the cytoplasmic and nuclear foci inside was performed using Fiji software (RRID:SCR_002285) (ImageJ (RRID:SCR_003070)).

### Immunofluorescent staining (IF)

T-47D and MDA-MB-453 breast cancer cells were seeded on top of sterilized coverslips in 6-well plates, serum-starved, and treated as described above for 4 h before fixation with 4% paraformaldehyde for 10 min at room temperature. Permeabilization was performed with 0.05% Triton X-100 (in PBS) for 1 h at room temperature. Dual immunofluorescence staining with AR (LSBio (LifeSpan) Cat# LS-B3326, RRID:AB_2060169) and GATA3 (Thermo Fisher Scientific Cat# MA1-028, RRID:AB_2536713) antibodies was performed at 4 °C, overnight. Antibody dilutions are listed in Additional file 9. On the following day, the cells were washed thrice (5 min each) in 1 × DPBS and incubated protected from light for 30 min at room temperature with secondary antibodies of Goat-anti-Rabbit (Alexa Fluor® 647; Life Technologies Cat# A-11036, RRID:AB_10563566) and Goat-anti-mouse (Alexa Fluor® 488; Life Technologies Cat# A-11029, RRID:AB_2534088) diluted in 10% goat serum at 1:400. Subsequently, the cells were washed as described above and incubated with Alexa Fluor® 568 Phalloidin (Invitrogen Cat# A12380, RRID not available) at 1:400 for 20 min in the dark at room temperature before DAPI staining (Thermo Fisher Scientific Cat# D1306, RRID: RRID:AB_2629482) and mounting onto glass slides with DAKO fluorescent mounting medium and sealing with clear nail polish.

Immunofluorescent stained slides were scanned through the ZEISS Axio Scan.Z1 Slide Scanner (RRID:SCR_020927) and imaged on an Olympus FV3000 confocal microscope. Representative images were processed using ZEN 3.0 (blue edition) software (ZEISS). All representative images were taken with the scale of 10 μm maintaining channel intensity range of DAPI in blue (black (250); white (2000)), GATA3 in green (black (400); white (1600)), and AR in red (black (130); white (260)). Images captured on a confocal microscope were acquired by using × 60 objective (with silicone immersion oil).

### Co-immunoprecipitation assay (co-IP)

ZR-75-1, T-47D, MFM-223, and MA-MB-453 cells were seeded at 9 × 10^6^ cells/plate, 9 × 10^6^ cells/plate, 11 × 10^6^ cells/plate, and 10 × 10^6^ cells/plate, respectively, in a 15-cm culture plate in normal growth media. After 24 h, the cells were washed twice with PBS and the media was replaced with phenol red-free DCC hormone starvation media. A PBS wash and media refresh was performed daily for 2 days. Cells were treated with ethanol or 10 nM DHT for 4 h before cross-linking with 1% formaldehyde, quenching with 2 M Glycine pH 7.5, and collection by scraping in PBS + protease inhibitors (Complete(R), Roche). The cells were suspended in lysis buffer (10 mM Tris–HCl (pH 8), 100 mM NaCl, 1 mM EDTA, 0.5 mM EGTA, 0.1% Na-Deoxycholate, and 0.5% N-lauryl sarcosine) plus protease inhibitors (Complete(R), Roche) and sonicated with the BioRuptor PLUS for 30 s on/off for 10 cycles. After centrifugation, the supernatant was collected and immunoprecipitated with protein A magnetic beads (Dynabeads®, Invitrogen) pre-bound with 5 µg/IP of GATA3 (Abcam Cat# ab199428, RRID:AB_2819013) or AR (Abcam Cat# ab108341, RRID:AB_10865716) at 4 °C overnight excluding the Input samples. The following day, the beads were washed 4 times with the wash buffer (20 mM Tris HCL Ph 8, 50 mM NaCl, 1 mM EDTA, 0.1% Tween 20, and 2 mM DTT (MW 154.25)) at room temperature, eluted with 30 μL of 0.2 M Glycine pH 2.6, neutralized with 1 M Tris–HCL pH 8, and boiled at 95 °C for 10 min to elute associated proteins, prior to analysis by Western blotting.

### Western blotting

Cells were harvested by scraping and lysed into Radioimmunoprecipitation assay (RIPA) buffer. Protein concentration was quantified by BCA protein assay (Thermo Fisher Scientific). Proteins were separated by SDS-PAGE on 10% Criterion™ TGX Stain Free-gels (BIO-RAD) and then transferred to nitrocellulose blotting membranes (GE Healthcare). The membrane was blocked in 5% skim milk, TBST for 2 h, followed by immunoblotting for AR (Santa Cruz Biotechnology Cat# sc-816, RRID:AB_1563391), ER (Santa Cruz Biotechnology Cat# sc-8002, RRID:AB_627558), GATA3 (Abcam Cat# ab199428, RRID:AB_2819013) overnight at 4 °C, or B-actin (Abcam Cat# ab6276, RRID:AB_2223210) and GAPDH (Millipore Cat# MAB374, RRID:AB_2107445) for 1 h at room temperature. Antibody dilutions are listed in Additional file 9. Membranes were washed with 0.1% Tween-20, PBS for 3 × 10 min. HRP-coupled secondary antibodies (Goat anti-Rabbit (Agilent Cat# P0448, RRID:AB_2617138; 1:2000; 1 h at room temperature) or Rabbit anti-Mouse (Agilent Cat# P0161, RRID:AB_2687969; 1:2000; 1 h at room temperature)) were detected with Clarity Western ECL Substrate (BIO-RAD) and visualized using a Bio Rad ChemiDoc MP Imaging System (RRID:SCR_019037). Veriblot (Abcam; Cat# ab131367, RRID:AB_2892718) was used as the secondary antibody for Co-IP (1:2000) samples before ECL detection.

### ChIP-sequencing (ChIP-seq)

ZR-75-1, T-47D, MFM-223, and MDA-MB-453 cells were seeded in 15 cm plates at 9 × 10^6^, 9 × 10^6^, 11 × 10^6^, and 10 × 10^6^ cells/plate, respectively, in their normal growth medium. Media was changed to phenol-red-free medium supplemented with 5% DCC-FBS after 24 h with daily media changes for 2 days prior to hormone treatment. Cells were then treated with either Vehicle (Ethanol) or 10 nM DHT (MFM-223 and MDA-MB-453) or Vehicle, 10 nM E2, 10 nM DHT, or combination E2 + DHT for 4 h prior to cross-linking and harvest. Each experiment was done in three independent biological replicates representing consecutive passages of cells. Additionally, frozen tumor tissues from experiments with the endocrine-resistant ER+ PDX model of breast cancer, GAR15-13D, were used to perform GATA3 ChIP-seq (Enobosarm vs Vehicle) as described in [8]. Tumors from Vehicle (*n* = 3) and Enobosarm (*n* = 3) treated animals harvested 5 days post treatment were cryo-sectioned before cross-linking. Cross-linking and sonication for all in vitro and in vivo models were carried out as described above, except PDX tumor samples were sonicated in the BioRuptor PICO 30 s on/off for 5.5 cycles. Immunoprecipitations were performed using 5 µg/IP of GATA3 (Abcam Cat# ab199428, RRID:AB_2819013) (for all in vitro and in vivo models) and AR (Abcam Cat# ab108341, RRID:AB_10865716) or 2 μg/IP of H3K27ac (Abcam Cat# ab4729, RRID:AB_2118291) antibodies. DNA was sequenced via Illumina NextSeq 500 (High Output) with 75 bp single-end reads, and resultant data was processed in Galaxy (RRID:SCR_006281). Briefly, trimmed FASTQ files were aligned to the hg19 genome assembly using Bowtie2 (version: 2.3.4.3, default parameters; RRID:SCR_016368); mapped reads with a minimum MAPQ > 10 and duplicate reads were removed using SAMTOOLS (RRID:SCR_002105); peaks were called using MACS2 callpeak (version: 2.1.1, default settings; RRID:SCR_013291), with a pooled input sample as the control. Only peaks found in at least 2 replicates were kept for the consensus peak-set. Heatmaps (Galaxy Version 3.3.2.0.1) and PCAs (Galaxy Version 3.3.2.0.0) were generated in Galaxy using Deeptools (RRID:SCR_016366). Peak annotations were performed using CisGenome (v2.0, RRID:SCR_001558). Motif analysis was performed on extracted fasta sequences using MEME ChIP [85] in discriminative mode against JASPAR CORE set of genes (2022). Differential binding analysis (DiffBind; RRID:SCR_012918) was performed in RStudio (RRID:SCR_000432) as described previously [16, 86] with peak annotations using ChIPseeker (RRID:SCR_021322) [87].

### ChIP-PCR

T-47D and MDA-MB-453 cells were seeded (8 × 10^6^, 10 ug/ 10^6^ cells/15 cm plate, respectively) in their normal growth media, which was changed to appropriate hormone-depleted media containing 5% DCC-FBS the following day. Forty-eight hours after seeding (DPBS wash and media refreshed each day), both cell line models were treated with Vehicle (0.001% EtOH) or 10 nM DHT for 4 h, followed by cross-linking, harvesting, and ChIP processing as described above. ChIP-PCR reactions were prepared using iQ SYBR Green Supermix (BIO-RAD) using primers listed in Additional file 9. PCR was performed with the CFX384 Real Time PCR Detection System (BIO-RAD, RRID:SCR_018057) and standard cycling conditions. ChIP-PCR data was analyzed by the percentage input method and/or fold enrichment over negative control as described previously [8].

For siRNA experiments, pre-designed siRNAs against GATA3, AR, and a negative (non-targeting) control siRNA (Qiagen Allstars Negative Control siRNA) were purchased commercially (Additional file 9); siGATA3-1 and siGATA3-2 (10 nM each) were used in T-47D cells; siGATA3-3 and siGATA3-4 (5 nM each) were used in MDA-MB-453 cells, versus 10 nM of siControl. Two siARs were used with the concentration of 10 nM for both cell line models as previously described [8]. Cells were transfected with siRNAs by reverse transfection using RNAiMAX (Invitrogen) (0.5 μL/cm^2^) at the time of seeding according to the manufacturer's protocols (Thermo Fisher Scientific, Waltham, MA, USA). The medium and transfection mix were changed the following day to a phenol red-free medium supplemented with 5% DCC-FBS. After 48 h (media refreshed each day), both cell models were treated for 4 h with Vehicle (0.001% EtOH) or 10 nM DHT, before cross-linking and harvesting as described above.

### RNA-seq

MFM-223 and MDA-MB-453 cells were seeded in 6-well dishes and treated for 6 h with Vehicle (0.001% EtOH) or 10 nM DHT prior to collection with TriReagent (Sigma). Six independent replicate experiments representing consecutive passages of cells were used to generate samples for RNA-seq. RNA was extracted from cells using the Direct-Zol RNA kit (Zymo Research). RNA integrity was assessed using the Experion RNA StdSens Analysis kit (Cat# 700–7111, BIO-RAD) on the Experion Automated Electrophoresis System (BIO-RAD, RRID:SCR_019691) and quantified by Nanodrop 2000 microvolume spectrophotometer (Thermo Scientific, RRID:SCR_018042). Total RNA was supplied to the Cancer Research UK Cambridge Institute (CRUK-CI) Genomics Core Facility for library preparation and high throughput sequencing. Conversion of the RNA into sequencing libraries was performed using the TruSeq® Total RNA HT kit (Illumina). Sequencing was performed using the Illumina HiSeq 2500 with single-end 40 bp reads. Raw data was trimmed using AdapterRemoval (RRID:SCR_011834) [88] and aligned to hg19 using STAR v2.7.5c (RRID:SCR_004463) [89]. Alignments were summarized to gene-level counts using featureCounts v2.0.1 (RRID:SCR_012919) [90] and gene annotations from Ensembl Release 101. QC at all stages was performed using FastQC and the R package ngsReports [91]. Counts were normalized for GC content and Gene length bias using CQN (RRID:SCR_001786) [92]. Differential expression analysis was performed using the Quasi-Likelihood models [93] with a range-based Null-Hypothesism [94] set with a threshold of 1.2 and an FDR of 0.05. GO Pathway analysis against the Molecular Signatures Database (MSigDB; RRID:SCR_016863) Hallmark gene list was performed via ShinyGO (RRID:SCR_019213) [95]. Upset plots were generated in RStudio (RRID:SCR_000432) using UpSetR [96].

### RNA extraction and quantitative real-time PCR (qRT-PCR)

T-47D and MDA-MB-453 cells were seeded at 0.5 × 10^6^ cells/well in a 6-well dish and hormone starved for 48 h followed by hormone treatment as described above. For siRNA experiments, cells were simultaneously transfected with siRNAs against GATA3 or AR. After treatment with vehicle (0.001% EtOH) or DHT (10 nM) for 6 h, total RNA was extracted with TriReagent (Sigma) followed by DNase treatment using the TURBO DNase Kit (Invitrogen) according to manufacturer’s protocol. Reverse transcription was performed with 500 ng of total RNA using the iScript cDNA Synthesis Kit (BIO-RAD). The resulting cDNA was diluted 1:10 and used for qRT-PCR and mRNA levels were normalized to *GAPDH* using the ΔΔCt method in BIO-RAD CFX-manager software. Additional file 9 outlines RT-PCR primers used herein.

### Immunohistochemistry (IHC)

Standard immunohistochemical techniques were performed as previously described [8] and employed using the following primary antibodies: AR (Santa Cruz Biotechnology Cat# sc-816, RRID:AB_1563391; 1:1000) and GATA3 (Santa Cruz Cat# sc-268, RRID:AB_2108591; 1:300). Appropriate positive and negative controls were included in all experiments. Tissue sections were incubated with primary antibody overnight at 4 °C; secondary antibodies and Streptavidin-HRP for 1 h at room temperature. The slides were scanned through the Nanozoomer S60 digital slide scanner slide scanner (Hamamatsu; RRID:SCR_022537).

### Statistical analyses

Survival plots were generated via KM Plotter using breast cancer mRNA data plotting each PAM50 subtype separately [97]. Statistical calculations were performed using GraphPad Prism (RRID: SCR_002798). Normality was assumed for all statistical tests unless otherwise stated. Multiple comparisons were adjusted for Tukey’s test, Student’s *t*-test, and ANOVA tests with post hoc corrections where appropriate. All tests were two-sided with a 95% confidence interval, and a *P* value < 0.05 was indicative of statistical significance.

### Supplementary Information

**Additional file 1.** AR RIME results.

**Additional file 2.** Supplementary figures.

**Additional file 3.** DiffBind results.

**Additional file 4.** AR-GATA3 co-occupied loci.

**Additional file 5.** Upset Plot intersections (gene lists) associated with AR/GATA3 co-bound loci (Additional file 2: Fig. SI).

**Additional file 6.** RNA-seq differential expression results.

**Additional file 7.** GO pathway analysis.

**Additional file 8.** Upset Plot intersections (gene lists) associated with Fig. 6C.

**Additional file 9.** Supplementary methods.

**Additional file 10.** Uncropped images for the blots in figure 1, additional file 2: supplementary figure s1, s6-8.

**Additional file 11.** Review history.

## Data Availability

The datasets generated during the current study are available in the Gene Expression Omnibus (GEO; RRID:SCR_005012) under accession number GSE176010 [98]. Proteomic datasets are included in this published article. The datasets analyzed from [8] are deposited in the Gene Expression Omnibus under accession # GSE123770 [99]. RNA-seq custom scripts have been deposited in the GitHub repository (RRID:SCR_002630) under GNU General Public License v3.0 [100–103] and under Zenodo [104–107]. Proteomic data have been deposited to the ProteomeXchange Consortium via the PRIDE [108] partner repository with the dataset identifier PXD047997 [109].
