# Supplementary material for: The androgen receptor interacts with GATA3 to transcriptionally regulate a luminal epithelial cell phenotype in breast cancer
Source: Genome Biol. 2024 Feb 5;25:44. doi: 10.1186/s13059-023-03161-y (PMC10840202; doi:10.1186/s13059-023-03161-y)
Supplement: Supplementary file 2 — Additional file 2. Supplementary figures. [file 13059_2023_3161_MOESM2_ESM.pdf]

Additional file 2: Supplementary figures

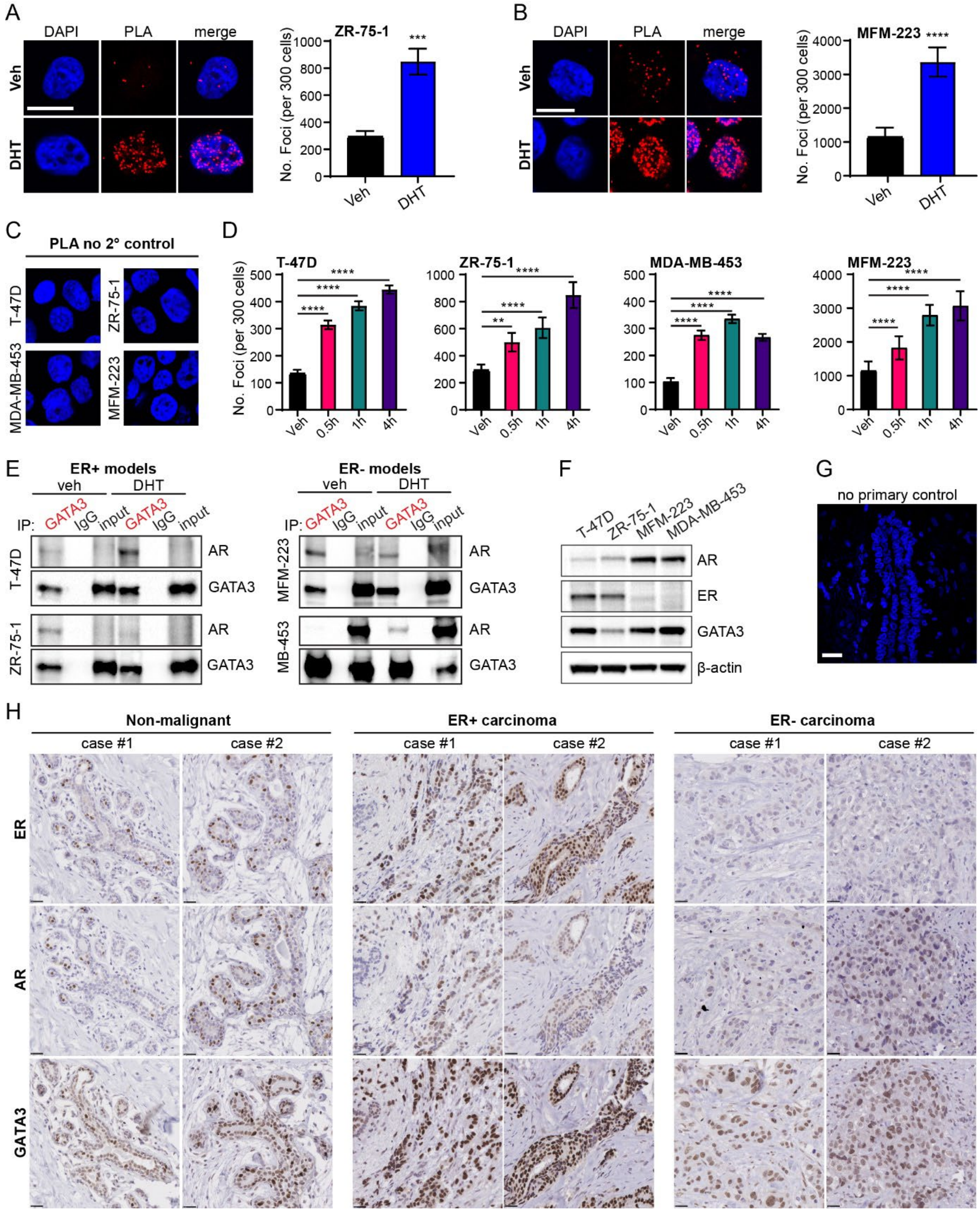

Fig. S1: GATA3 and AR interact in cell line and patient-derived models of breast cancer. Proximity ligation assays (PLA) showing AR-GATA3 interactions in **(A)** ZR-75-1 (ER+) and **(B)** MFM-223 (ER-) breast cancer cell lines treated with Veh or DHT (10 nM). Scale bar = 20μm.

Quantification represented as mean foci per 300 nuclei ± SEM (n=5 technical replicates) analyzed by one-sided unpaired t-test (***$P<0.001$; ****$P<0.0001$). **(C)** Representative images of the no secondary control used for PLA to confirm specificity of the interaction. **(D)** Quantification of mean PLA foci per 300 nuclei after DHT treatment for 30 min, 1 h or 4 h. Analyzed by one-sided unpaired t-test (**$p<0.01$; ****$p<0.0001$). **(E)** Western blot showing co-immunoprecipitation of AR and GATA3 in ER+ and ER- breast cancer cell lines treated with Veh (EtOH) or DHT (10 nM). **(F)** Protein levels of AR, ER and GATA3 in ER+ (T-47D, ZR-75-1) and ER- (MDA-MB-453, MFM-223) cell lines. **(G)** Representative image of the no primary control used for the tissue PLA. Scale bar = 30 μm. **(H)** IHC for ER, AR and GATA3 in non-malignant and malignant (ER+ and ER-) clinical breast tissues matching Figure 1G.

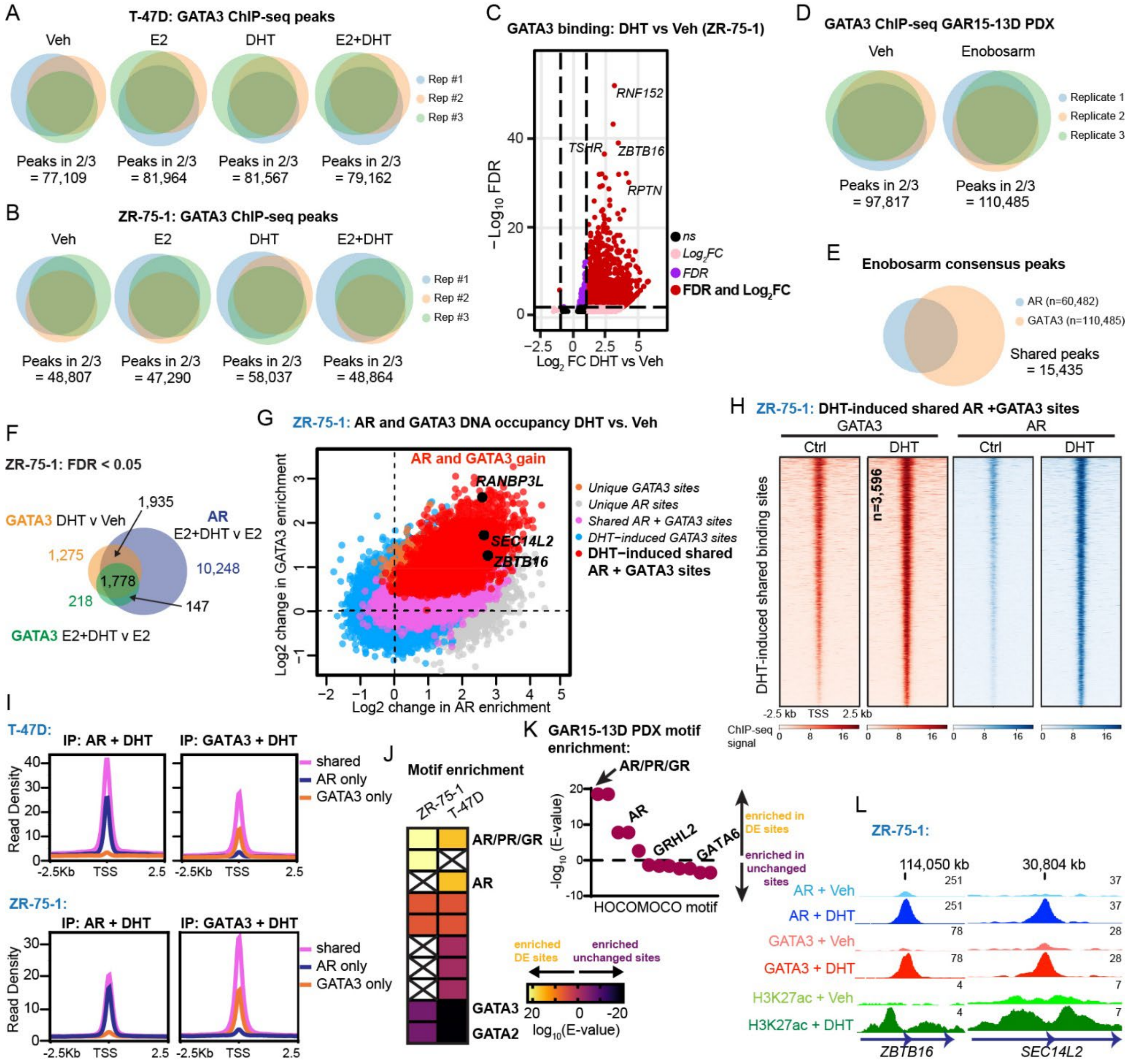

Fig. S2: Activation of AR induces GATA3 chromatin binding at AR/GATA3 co-occupied loci in ZR-75-1 breast cancer cells. Replicate data for GATA3 ChIP-seq *in vitro* experiments in T-47D **(A)** and ZR-75-1 **(B)** cells associated with Fig 2. Venn diagrams show the overlap of three independent experiments representing consecutive passages of cells treated with vehicle (Veh; EtOH), estradiol (E2; 10 nM), DHT (10 nM) or E2 + DHT (10 nM each). Peaks present in at least 2 of 3 replicates were used to generate a consensus cistrome, indicated below the Venn diagrams, for further comparative analyses. **(C)** Volcano plot reporting the FDR adjusted p-value and the log_2FC of GATA3 chromatin binding events in ZR-75-1 (ZR)

breast cancer cells treated with DHT vs Veh. For visualization of differential binding patterns, the threshold for gain or loss in volcano plots is shown as fold-change > 1 (vertical lines) with an FDR cut-off of 5 x 10^{-3} (horizontal line). **(D)** Replicate data for GATA3 ChIP-seq in GAR15-13D ER+ PDX associated with Fig 2. Venn diagrams show the overlap of three independent PDX treated with Veh or DHT for 5 days as described in [8]. Peaks present in at least 2 of 3 replicates were used to generate a consensus cistrome, indicated below the Venn diagrams, for further comparative analyses. **(E)** Venn diagram showing overlap of consensus AR and GATA3 cistromes in enobosarm treated PDX. **(F)** Venn diagram showing overlap of significantly enriched (FDR < 0.05, no fold-change threshold) AR and GATA3 binding sites after stimulation with DHT. **(G)** Two-factor log-ratio (M) plot displaying DHT-induced changes in GATA3 and AR enrichment at consensus chromatin binding sites in ZR-75-1 cells. Point color denotes treatment-induced changes in transcription factor occupancy (called peaks) as in Fig 3 where shared AR/GATA3 peaks that are gained with DHT stimulation are denoted in red. Point co-ordinates are derived from the average enrichment score of three independent ChIP-seq replicates for each consensus binding site. Example binding sites near known AR target genes are highlighted. **(H)** Consensus GATA3 and AR ChIP-seq heatmaps demonstrative of a DHT-induced gain in GATA3 chromatin binding sites that are shared with AR. **(I)** Average read density plots for AR (left) and GATA3 (right) chromatin occupancy proximal (<100 kb) to unique or shared genes in T-47D cells (top) and ZR-75-1 cells (bottom) treated *in vitro* with DHT. Motif enrichment of shared DHT-induced AR/GATA3 binding sites *in vitro* (T-47D and ZR-75-1) **(J)** and *in vivo* **(K)**. **(L)** Example genome browser images showing GATA3, AR and H3K27ac ChIP-seq signals at loci associated with AR target genes *ZBTB16* (left panel) and *SEC14L2* (right panel) in ZR-75-1 cells. Data represents the average signal of three replicates.

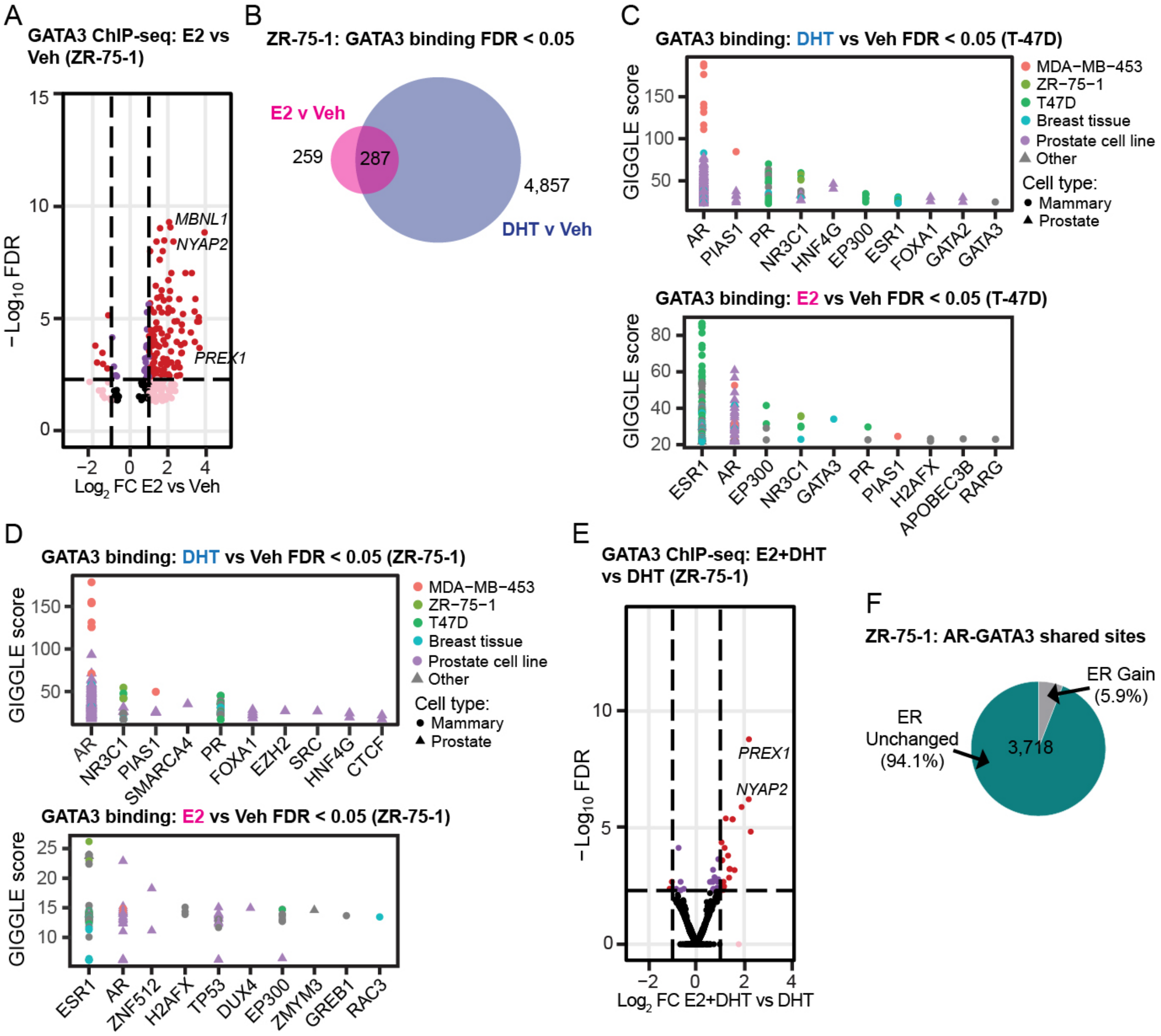

Fig. S3: GATA3 chromatin occupancy is altered by hormone treatment of ER-positive breast cancer cells. (A) Volcano plot reporting the FDR adjusted p-value and the log2FC of GATA3 chromatin binding events in ZR-75-1 breast cancer cells treated *in vitro* with E2 vs Veh. Threshold is set as in Figure 2. **(B)** Venn diagram showing the overlap of significantly enriched (FDR < 0.05) GATA3 binding sites with E2 or DHT in ZR-75-1 cells. **(C)** Transcription factor binding score at GATA3 DHT-induced loci (FDR < 0.05) or E2-induced loci in T-47D cells or **(D)** ZR-75-1 cells. Circles represent public data from breast cancer cell lines/tissue while triangles are derived from prostate cancer cell data, with color denoting cell line. **(E)** Volcano plot reporting the FDR adjusted p-value and the log_2FC of GATA3 chromatin binding events with simultaneous hormone treatment (E2+DHT) compared to DHT alone in ZR-75-1. For visualization of differential binding patterns, the threshold for gain or loss in volcano plots

is shown as in Figure 2. **(F)** Differential ER binding (from [8], FDR < 0.05, no fold-change threshold) at AR and GATA3 shared sites.

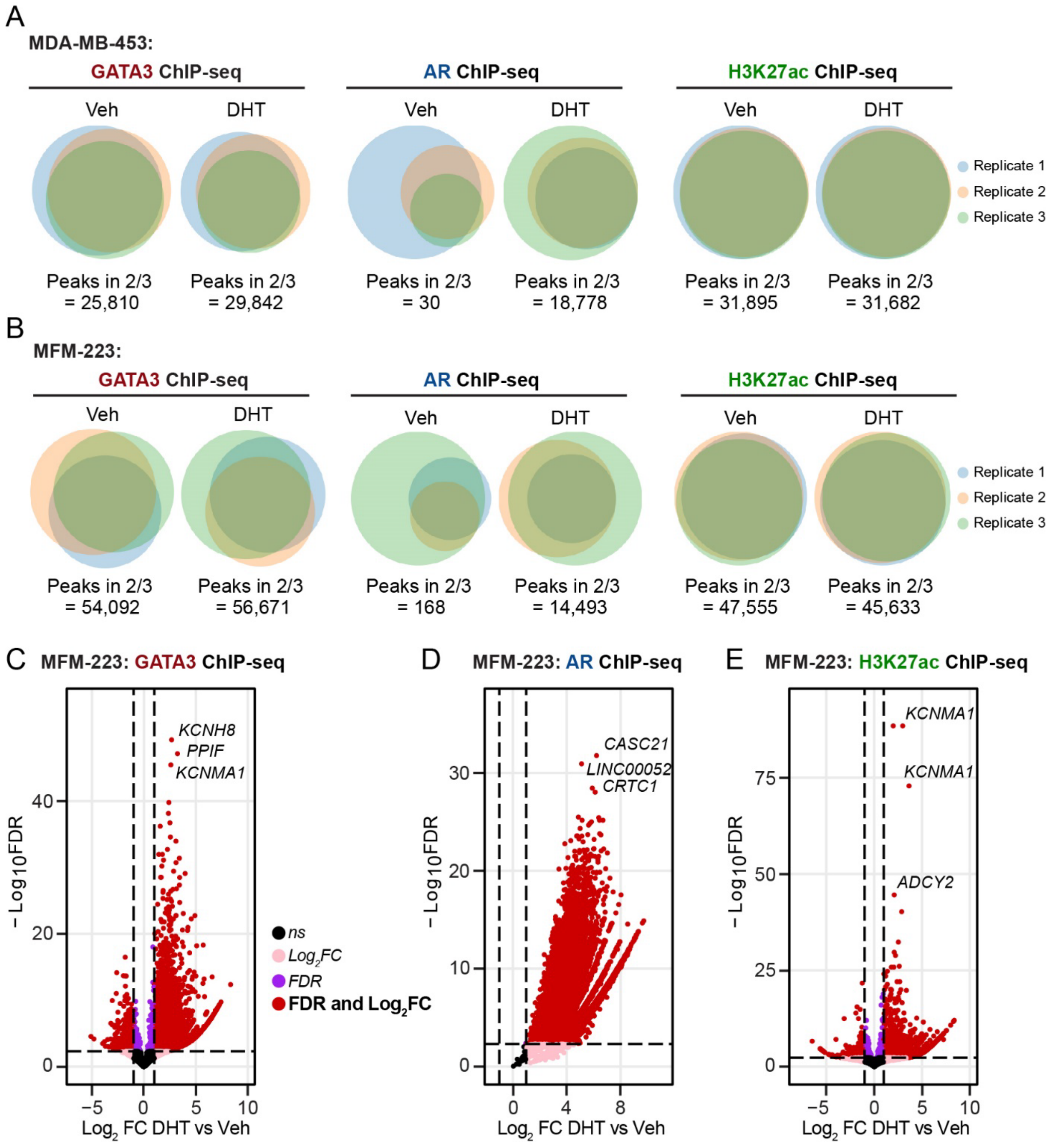

Fig. S4: Activation of AR induces changes in GATA3, AR and H3K27ac chromatin binding in ER-negative breast cancer cells. Replicate data for GATA3 ChIP-seq, AR ChIP-seq and H3K27ac ChIP-seq in MDA-MB-453 **(A)** cells and MFM-223 **(B)** cells. Venn diagrams show the overlap of three independent experiments representing consecutive passages of

cells treated with Veh or DHT (10 nM). Peaks present in at least 2 of 3 replicates were used to generate a consensus cistrome, indicated below the Venn diagrams, for further comparative analyses. Volcano plot reporting the FDR adjusted p-value and the log2FC of GATA3 **(C)**, AR **(D)**, and H3K27ac **(E)** chromatin binding events in MFM-223 breast cancer cells treated with DHT vs Veh. The threshold for denoting gain or loss by dot color is set as in Figure 2.

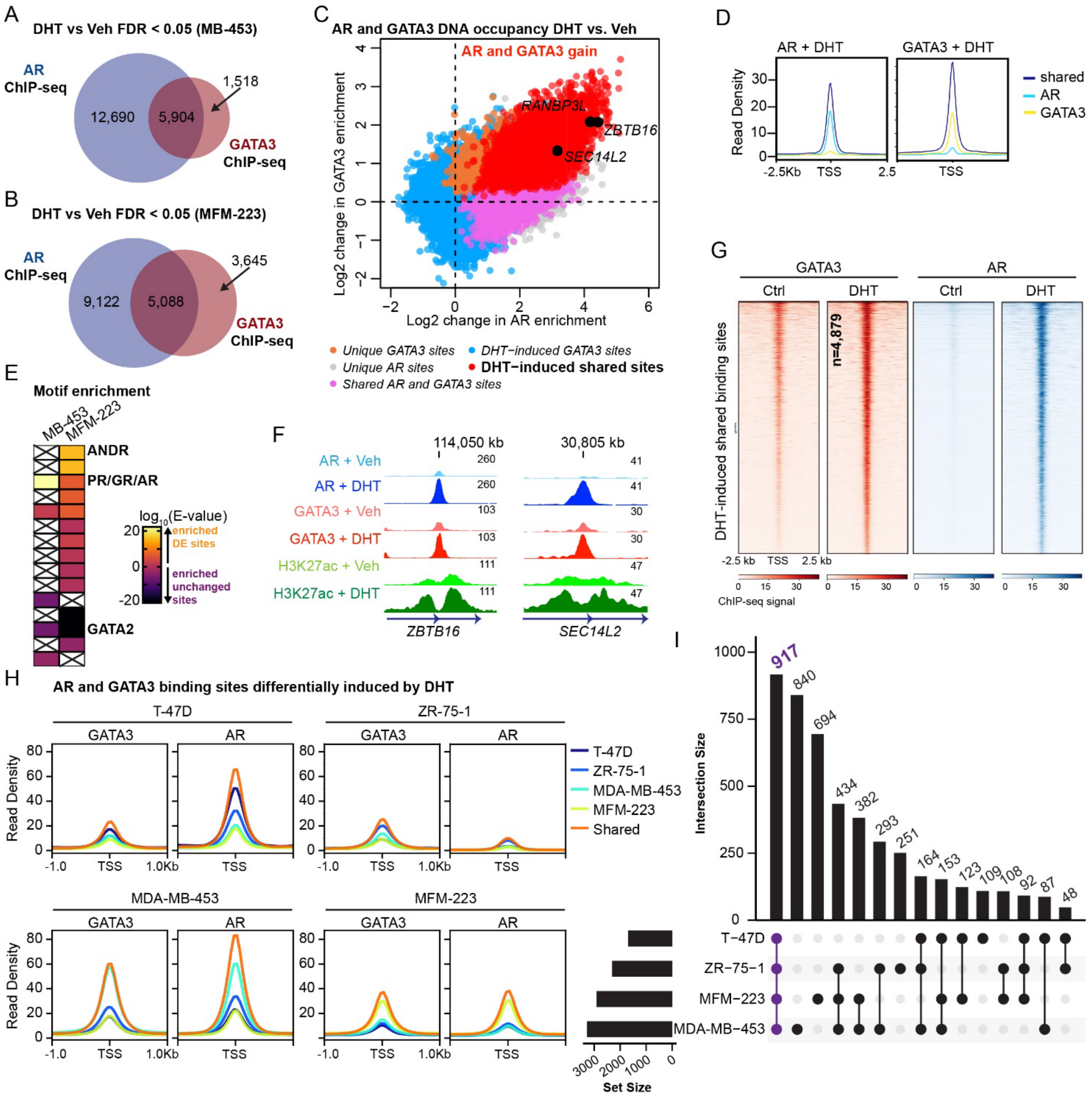

Fig. S5: Activation of AR induces binding at shared AR and GATA3 loci in ER- breast cancer cell lines. Venn diagram showing overlap of GATA3 and AR binding sites significantly

enriched (FDR < 0.05) by DHT treatment in MDA-MB-453 **(A)** and MFM-223 **(B)** cells. **C)** Two-factor log-ratio (M) plot displaying DHT-induced changes in GATA3 and AR enrichment at chromatin binding sites in MFM-223 cells. Point color denoting treatment-induced transcription factor occupancy (called peaks) as described in Figure 3, where red dots indicate loci in which AR and GATA3 are enriched following treatment with DHT. **D)** Average read density plots for AR (left) and GATA3 (right) chromatin occupancy proximal (<100 kb) to unique or shared genes in MDA-MB-453 cells *in vitro* treated with DHT. **(E)** Motif enrichment of shared DHT-induced AR/GATA3 binding sites in ER- cell lines. **(F)** Example genome browser images showing GATA3, AR and H3K27ac ChIP-seq signals at binding sites associated with *KDM4B* (left panel), *EHF* (middle panel) and *CNTNAP2* (right panel) in MFM-223 cells. Data represents the average signal of three replicates. **(G)** Consensus GATA3 and AR ChIP-seq data from (C) showing heatmaps demonstrative of a DHT-induced gain in GATA3 chromatin binding sites that are shared with AR. **(H)** Read density plot comparing DHT-induced AR-GATA3 DNA binding patterns between cell lines and **(I)** overlap of the associated annotated genes. Shared loci are indicated by orange line in read density plots and common genes are denoted in purple.

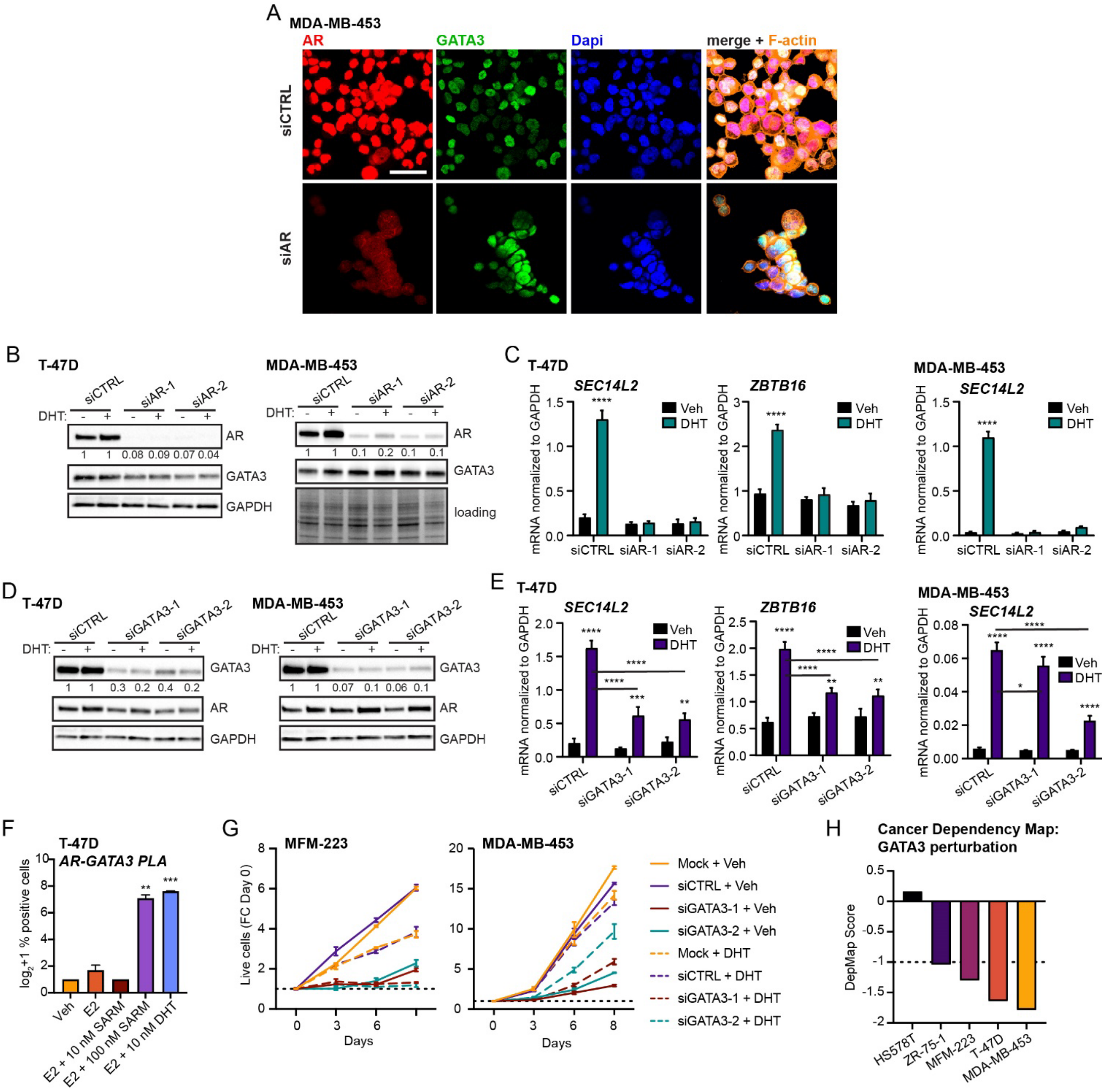

Fig. S6: GATA3 promotes, but is not essential for, expression of shared AR and GATA3 target genes. (A) Representative immunofluorescence images showing AR silencing in MDA-MB-453 cells. Scale bar = 30 µm. **(B)** Western blot confirming AR silencing in T-47D and MDA-MB-453 cells. **(C)** mRNA expression of DHT-regulated AR target genes following AR silencing by two different siRNAs in T-47D cells (upper panel) and MDA-MB-453 cells, treated *in vitro* with Veh (EtOH) or DHT related to Fig 5B. Data was analyzed by a two-way ANOVA. Post-hoc analyses were performed using Tukey's multiple comparisons test, where *SEC14L2*, $P < 0.0001$ for Veh versus DHT in siControl only; *ZBTB16*, $P < 0.0001$ and $P < 0.01$ for Veh

versus DHT in siControl and siAR-1, respectively. **(D)** Western blot confirming GATA3 silencing in T-47D and MDA-MB-453 cells. **(E)** mRNA expression of DHT-regulated genes following GATA3 knockdown in T-47D cells (upper panel) and MDA-MB-453 cells (lower panel), treated *in vitro* with Veh (EtOH) or DHT related to Fig 5C. Data was analyzed by a two-way ANOVA. Post-hoc analyses were performed using Tukey's multiple comparisons test, where *SEC14L2*, $P < 0.0001$, and $P<0.01$ for Veh versus DHT in siControl and siGATA3, respectively, and $P<0.0001$ for siControl + DHT versus siAR + DHT; *ZBTB16*, $P < 0.0001$ for Veh versus DHT in siControl and siGATA3, and $P<0.0001$ for siControl + DHT versus siGATA3 + DHT. Data is shown as mean ± SEM of 3 biological replicates from consecutive passages of cells. **(F)** Proximity ligation assays (PLA) showing AR-GATA3 interactions in T-47D cells treated with Veh (EtOH), estradiol (E2; 10 nM), or E2 in combination with 10 nM enobosarm (SARM), 100 nM enobosarm, or 10 nM. Quantification represented as log2+1 transformed percentage of cells that are positive with at least 1 interaction (n=3 technical replicates) analyzed by one-way nonparametric ANOVA with post-hoc corrections (** $P<0.01$; *** $P<0.001$). **(G)** Representative growth curves showing proliferation of MFM-223 (left) and MDA-MB-453 (right) cell lines in response to Veh versus DHT (10 nM) following GATA3 knock-down with two separate siRNAs. Data was compared using a two-way repeated measures ANOVA. **(H)** DepMap dependency score (CRISPR) for GATA3 in the four cell lines used in this manuscript. HS578T cells are included as a negative control (i.e., not dependent on GATA3).

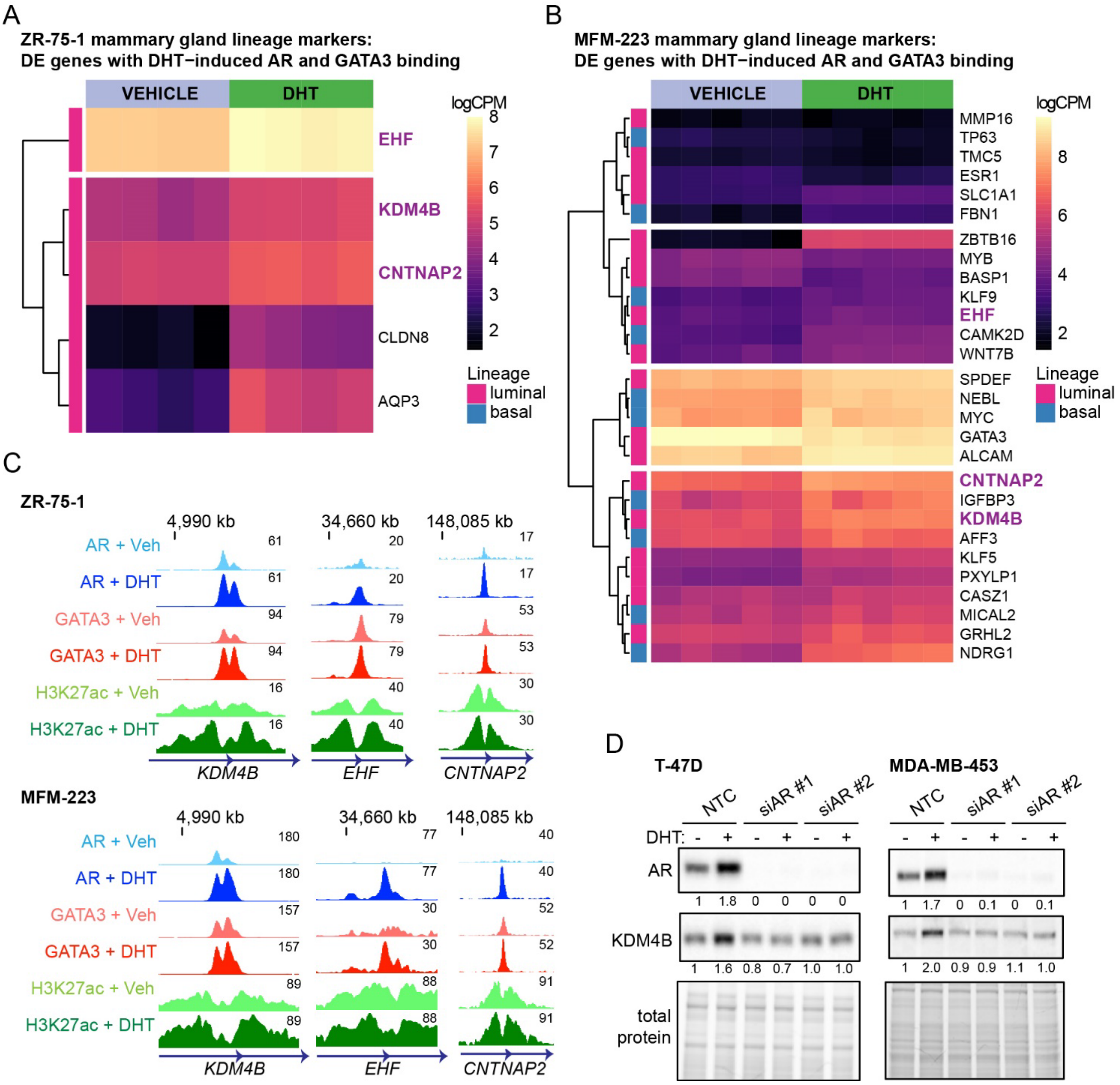

Fig. S7: AR agonist-induced shared AR and GATA3 target genes are associated with development and differentiation of mammary epithelium. (A) RNA-seq heatmap of differentially expressed (FDR < 0.05) mammary lineage marker genes with DHT-induced shared AR and GATA3 binding in ZR-75-1 and **(B)** MFM-223 cells. Luminal marker genes are denoted by pink squares (y-axis) and basal marker genes are denoted by blue squares. Gene expression is represented as the log counts per million (logCPM) to observe patterns of expression between genes (rows). Genes common to all ER+ (T-47D, ZR-75-1) and ER- (MDA-MB-453, MFM-223) cell lines are indicated in purple. **(C)** Example genome browser images showing averaged GATA3, AR and H3K27ac ChIP-seq signals at binding sites

associated with *KDM4B* (left panel), *EHF* (middle panel) and *CNTNAP2* (right panel) in ZR-75-1 and MFM-223 cells. Data represents the average signal of three replicates. **(D)** Western blot for AR and KDM4B in T-47D cells and MDA-MB-453 cells treated with Veh or DHT (10 nM) after AR silencing.

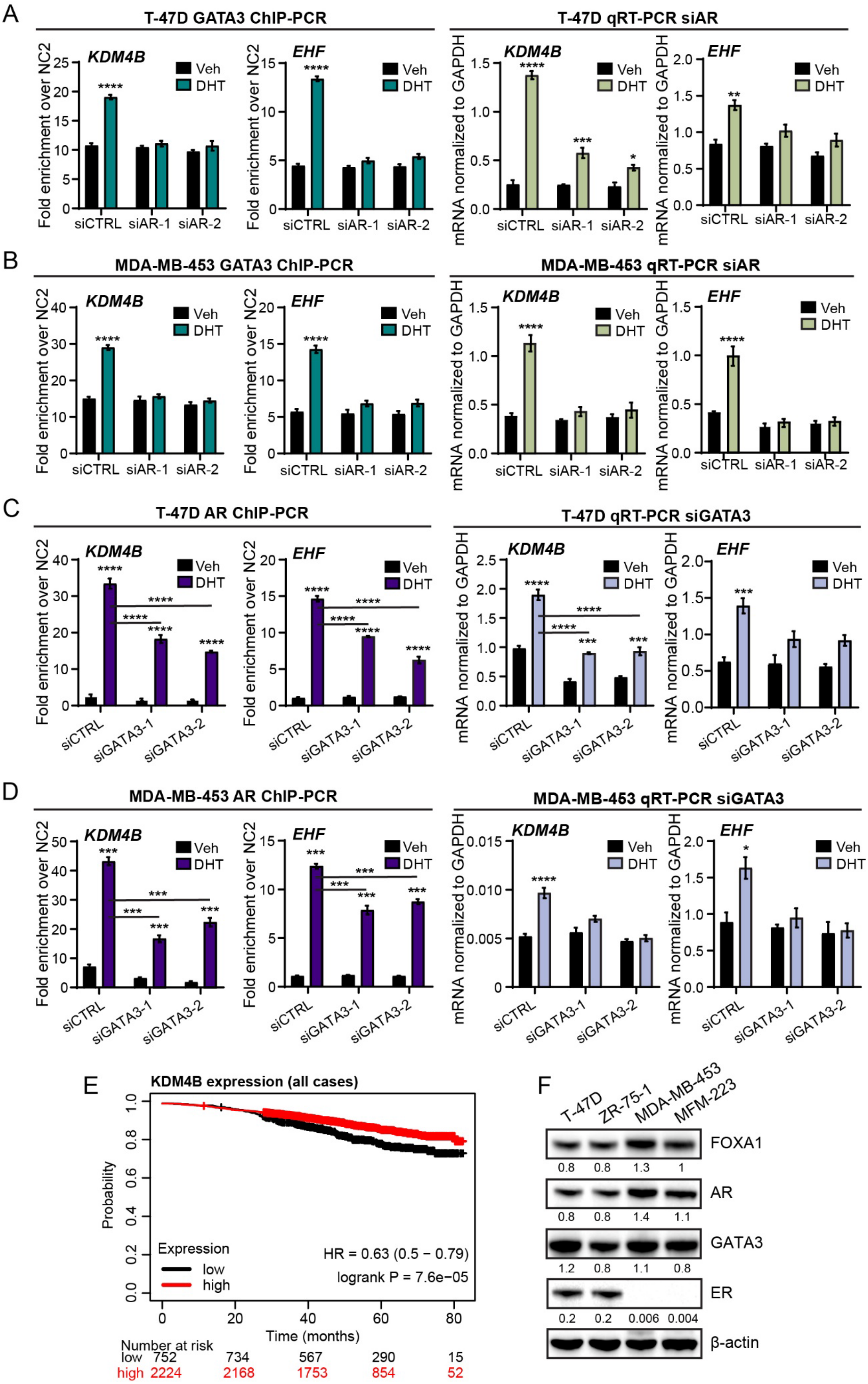

A
T-47D GATA3 ChIP-PCR
T-47D qRT-PCR siAR
B
MDA-MB-453 GATA3 ChIP-PCR
MDA-MB-453 qRT-PCR siAR
C
T-47D AR ChIP-PCR
T-47D qRT-PCR siGATA3
D
MDA-MB-453 AR ChIP-PCR
MDA-MB-453 qRT-PCR siGATA3
KDM4B
EHF
Veh
DHT
Fold enrichment over NC2
mRNA normalized to GAPDH
siCTRL
siAR-1
siAR-2
siGATA3-1
siGATA3-2
E
KDM4B expression (all cases)
Probability
Expression
low
high
HR = 0.63 (0.5 − 0.79)
logrank P = 7.6e−05
Time (months)
Number at risk
low 752 734 567 290 15
high 2224 2168 1753 854 52
F
T-47D
ZR-75-1
MDA-MB-453
MFM-223
FOXA1
0.8 0.8 1.3 1
AR
0.8 0.8 1.4 1.1
GATA3
1.2 0.8 1.1 0.8
ER
0.2 0.2 0.006 0.004
β-actin

Fig. S8. GATA3 loss disrupts expression of shared luminal epithelial shared AR/GATA3 target genes. GATA3 ChIP-PCR at AR/GATA3 co-occupied loci and resultant mRNA expression of associated luminal lineage genes following AR silencing by two different siRNAs in T-47D **(A)** and MDA-MB-453 **(B)** cells treated *in vitro* with Veh (EtOH) or DHT. Data was analyzed by a two-way ANOVA. Post-hoc analyses were performed using Tukey's multiple comparisons test, where **** indicates $P < 0.0001$; *** indicates $P < 0.001$; * indicates $P<0.05$. AR ChIP-PCR at AR/GATA3 co-occupied loci and resultant mRNA expression of associated luminal lineage genes following GATA3 knockdown in T-47D **(C)** and MDA-MB-453 **(D)** cells treated *in vitro* with Veh (EtOH) or DHT. Data was analyzed by a two-way ANOVA. Post-hoc analyses were performed using Tukey's multiple comparisons test, where **** indicates $P < 0.0001$ and *** indicates $P < 0.001$. Data is shown as mean ± SEM of 3 biological replicates from consecutive passages of cells. **(E)** Survival analyses using the KM plotter mRNA database to stratify *KDM4B* expression in breast cancer (all cases). **(F)** Western blot for FOXA1, AR, GATA3 and ER in ER+ (T-47D, ZR-75-1) and ER- (MDA-MB-453, MFM-223) cycling cell lines grown in regular culture media.
