# Supplementary material for: The androgen receptor interacts with GATA3 to transcriptionally regulate a luminal epithelial cell phenotype in breast cancer
Source: Genome Biol. 2024 Feb 5;25:44. doi: 10.1186/s13059-023-03161-y (PMC10840202; doi:10.1186/s13059-023-03161-y)

**GBIO-D-23-00307R1: Key to raw western blot files**

**Figure 1**

Files provided:

1. **T-47D; AR:** Fig 1F 2019-12-02 12hr 41min_Exp_4.0sec T47 ZR AR IP AR WB

Samples by lane:

1. ZR-75-1: Veh, AR IP
2. ZR-75-1: Veh, IgG IP
3. ZR-75-1: Veh, Input
4. ZR-75-1: DHT, AR IP
5. ZR-75-1: DHT, IgG IP
6. ZR-75-1: DHT, Input
7. Empty lane
8. T-47D: Veh, AR IP
9. T-47D: Veh, IgG IP
10. T-47D: Veh, Input
11. T-47D: DHT, AR IP
12. T-47D: DHT, IgG IP
13. T-47D: DHT, Input
14. **T-47D; GATA3:** Fig 1F 2019-12-03 19hr 15min_Exposure_29.0sec_T47 ZR AR IP GATA3 WB

Samples by lane: as outlined in (a).

1. **ZR-75-1; AR:** Fig 1F 2019-12-02 12hr 41min_Exp_4.0sec T47 ZR AR IP AR WB

Samples by lane: as outlined in (a).

1. **ZR-75-1; GATA3:** Fig 1F 2019-12-03 19hr 15min_Exposure_29.0sec_T47 ZR AR IP GATA3 WB

Samples by lane: as outlined in (a).

1. **MFM-223; AR:** Fig1F AUTO 2 -Chemidoc 2020-04-21 13hr 07min 223 AR and GAT

Samples by lane:

1. Veh: AR IP
2. Veh: IgG IP
3. Veh: Input
4. DHT: AR IP
5. DHT: IgG IP
6. DHT: Input
7. **MFM-223; GATA3:** Fig1F AUTO 2 -Chemidoc 2020-04-21 13hr 07min 223 AR and GAT

Samples by lane: as outlined in (e) above.

1. **MDA-MB-453; AR:** Fig 1 453 AR_Chemidoc 2019-12-02 13hr 06min_Exp_1.0sec

Samples by lane:

1. Veh: AR IP
2. Veh: IgG IP
3. Veh: Input
4. Empty lane
5. DHT: AR IP
6. DHT: IgG IP
7. DHT: Input
8. Empty lane
9. Veh: GATA3 IP
10. Veh: IgG IP
11. Veh: Input
12. Empty lane
13. DHT: GATA3 IP
14. DHT: IgG IP
15. DHT: Input
16. **MDA-MB-453; GATA3:** Fig 1 Fig S1 453-GATA BAND_Exposure_20.0sec

Samples by lane: as outlined in (g) above.

**Figure S1E**

Files provided:

1. **T-47D; AR:** Fig S1E 2020-01-26 14hr 45min_Exp_18.0sec T47 AR

Samples by lane:

1. DHT: Input
2. DHT: IgG IP
3. DHT: GATA3 IP
4. Veh: Input
5. Veh: IgG IP
6. Veh: GATA3 IP
7. **T-47D; GATA3:** Fig S1E T47 GATA Chemidoc 2020-01-25 17hr 39min_Exp_20.0sec

Samples by lane: as outlined in (a) above.

1. **ZR-75-1; AR:** Fig S1E ZR AR Chemidoc 2020-01-26 14hr 35min_Exp_10.0sec

Samples by lane:

1. DHT: Input
2. DHT: IgG IP
3. DHT: GATA3 IP
4. Veh: Input
5. Veh: IgG IP
6. Veh: GATA3 IP
7. **ZR-75-1; GATA3:** Fig S1E ZR GATA3 Chemidoc 2020-01-25 17hr 32min_Exp_29.0sec

Samples by lane: as outlined in (c) above.

1. **MFM-223; AR:** Fig S1E Auto-Chemidoc 2020-04-21 12hr 57min 223 AR

Samples by lane:

1. DHT: Input
2. DHT: IgG IP
3. DHT: GATA3 IP
4. Veh: Input
5. Veh: IgG IP
6. Veh: GATA3 IP
7. **MFM-223; GATA3:** Fig S1E Auto - Chemidoc 2020-04-20 11hr 51min 223 GATA3

Samples by lane: as outlined in (e) above.

1. **MDA-MB-453; AR:** Fig S1 453 AR_Chemidoc 2019-12-02 13hr 06min_Exp_1.0sec

Samples by lane:

1. Veh: AR IP
2. Veh: IgG IP
3. Veh: Input
4. Empty lane
5. DHT: AR IP
6. DHT: IgG IP
7. DHT: Input
8. Empty lane
9. Veh: GATA3 IP
10. Veh: IgG IP
11. Veh: Input
12. Empty lane
13. DHT: GATA3 IP
14. DHT: IgG IP
15. DHT: Input
16. **MDA-MB-453; GATA3:** Fig 1 Fig S1 453-GATA BAND_Exposure_20.0sec

Samples by lane: as outlined in (g) above.

**Figure S1F**

Files provided:

1. **GATA3:** Fig S1F AR GATA3 Chemidoc 2019-02-03 14hr 02min_Exposure_7.4sec
2. **AR:** Fig S1F AR GATA3 Chemidoc 2019-02-03 14hr 02min_Exposure_7.4sec
3. **ER:** Fig S1F ER Chemidoc 2019-02-04 11hr 50min_Exposure_19.3sec
4. **β-actin:** Fig S1F actin Chemidoc 2019-02-06 12hr 45min

Samples by lane (all files):

1. T-47D
2. ZR-75-1
3. MFM-223
4. MDA-MB-453

**Figure S6B**

Files provided:

1. **T-47D; siAR:** Fig S6 T47D-siAR-Chemidoc 2021-01-25 14hr 42min_Exposure_7.0sec where the top band is AR western blot and the bottom is GATA3 western blot

Samples by lane:

1. siControl + veh
2. siControl + DHT
3. siAR-1 + veh replicate #1
4. siAR-1 + DHT replicate #1
5. siAR-2 + veh replicate #1
6. siAR-2 + DHT replicate #1
7. siAR-1 + veh replicate #2
8. siAR-1 + DHT replicate #2
9. siAR-2 + veh replicate #2
10. siAR-2 + DHT replicate #2
11. siAR-1 + veh replicate #3
12. siAR-1 + DHT replicate #3
13. siAR-2 + veh replicate #3
14. siAR-2 + DHT replicate #3
15. **T-47D; GAPDH siAR and siGATA3:** Fig S6 T47D-GAPDH-siAR top- siGATA3 bottom-Exposure_3.0sec

Samples by lane: as outlined above in (a).

1. **MDA-MB-453; siAR, AR western blot:** Fig S6 453-siAR-AR

Samples by lane:

1. siControl + veh replicate #1
2. siControl + DHT replicate #1
3. siControl + veh replicate #2
4. siControl + DHT replicate #2
5. siAR-1 + veh replicate #1
6. siAR-1 + DHT replicate #1
7. siAR-2 + veh replicate #1
8. siAR-2 + DHT replicate #1
9. siAR-1 + veh replicate #2
10. siAR-1 + DHT replicate #2
11. siAR-2 + veh replicate #2
12. siAR-2 + DHT replicate #2
13. siAR-1 + veh replicate #3
14. siAR-1 + DHT replicate #3
15. siAR-2 + veh replicate #3
16. siAR-2 + DHT replicate #3
17. **MDA-MB-453; siAR, GATA3 western blot:** Fig S6 453-siAR-GATA3

Samples by lane: as outlined above in (a).

1. **MDA-MB-453; total protein loading control siAR:** Fig S6 453-siAR-stainfreegel

Samples by lane: as outlined above in (a).

**Figure S6D**

Files provided:

1. **T-47D; siGATA3:** Fig S6 T47D-siGATA3-Chemidoc 2021-01-25 14hr 53min_Exposure_10.1sec where the top band is AR western blot and the bottom is GATA3 western blot

Samples by lane:

1. Spill over from siControl + veh
2. siControl + veh
3. siControl + DHT
4. siGATA3-1 + veh replicate #1
5. siGATA3-1 + DHT replicate #1
6. siGATA3-2 + veh replicate #1
7. siGATA3-2 + DHT replicate #1
8. siGATA3-1 + veh replicate #2
9. siGATA3-1 + DHT replicate #2
10. siGATA3-2 + veh replicate #2
11. siGATA3-2 + DHT replicate #2
12. siGATA3-1 + veh replicate #3
13. siGATA3-1 + DHT replicate #3
14. siGATA3-2 + veh replicate #3
15. siGATA3-2 + DHT replicate #3
16. **T-47D; GAPDH siAR and siGATA3:** Fig S6 T47D-GAPDH-siAR top- siGATA3 bottom-Exposure_3.0sec

Samples by lane: as outlined above in (a).

1. **MDA-MB-453; siGATA3, AR and GATA3 western blot:** Fig S6 453 siGATA3 AR GAT 2021-01-25 15hr 00min_Exp_10.1sec where the top band is AR western blot and the bottom is GATA3 western blot

Samples by lane:

1. siAR-2 + DHT replicate #3
2. siAR-2 + veh replicate #3
3. siAR-1 + DHT replicate #3
4. siAR-1 + veh replicate #3
5. siAR-2 + DHT replicate #2
6. siAR-2 + veh replicate #2
7. siAR-1 + DHT replicate #2
8. siAR-1 + veh replicate #2
9. siAR-2 + DHT replicate #1
10. siAR-2 + veh replicate #1
11. siAR-1 + DHT replicate #1
12. siAR-1 + veh replicate #1
13. siControl + DHT
14. siControl + veh
15. **MDA-MB-453; siAR, GAPDH:** Fig S6 453 siGATA3 GAPDH 2021-01-25 17hr 29min_Exp_2.0sec

Samples by lane: as above for (b)

**Figure S7**

Files provided:

1. **T-47D; AR:** Fig S7 GLL372 GLL373 T-47D AR 3 sec

Samples by lane:

1. siControl veh replicate #1
2. siControl DHT replicate #1
3. siAR-1 veh replicate #1
4. siAR-1 DHT replicate #1
5. siAR-2 veh replicate #1
6. siAR-2 DHT replicate #1
7. siControl veh replicate #2
8. siControl DHT replicate #2
9. siAR-1 veh replicate #2
10. siAR-1 DHT replicate #2
11. siAR-2 veh replicate #2
12. siAR-2 DHT replicate #2
13. empty lane
14. – 25 other samples not related to this manuscript
15. **T-47D; KDM4B:** Fig S7 GLL372 GLL373 T-47D KDM4B blot 10 sec

Samples by lane: as outlined in (a) above.

1. **T-47D; total protein:** Fig S7 GLL372 GLL373 T-47D gel

Samples by lane: as outlined in (a) above.

1. **MDA-MB-453; AR:** Fig S7 GLL372 GLL373 MDA-MB-453 siAR AR

Samples by lane:

1. siControl veh replicate #1
2. siControl DHT replicate #1
3. siAR-1 veh replicate #1
4. siAR-1 DHT replicate #1
5. siAR-2 veh replicate #1
6. siAR-2 DHT replicate #1
7. empty lane
8. siControl veh replicate #2
9. siControl DHT replicate #2
10. siAR-1 veh replicate #2
11. siAR-1 DHT replicate #2
12. siAR-2 veh replicate #2
13. siAR-2 DHT replicate #2
14. **MDA-MB-453; KDM4B:** Fig S7 GLL372 GLL373 MDA-MB-453 siAR KDM4B 10 sec

Samples by lane: as outlined in (d)

1. **MDA-MB-453; total protein:** Fig S7 GLL372 GLL373 MDA-MB-453 siAR gel

Samples by lane: as outlined in (d)

**Figure S8**

Files provided:

1. **FOXA1:** Fig S8 FOXA1_3seconds_20221214 CP
2. **AR:** Fig S8 AR_11seconds_20221214 CP
3. **GATA3:** Fig S8 GATA3_7seconds_20221214 CP
4. **ER:** Fig S8 ER_38seconds_20221214 CP
5. **β-actin:** Fig S8 BActin_5seconds_1_LHS_2_RHS_20221215 CP

Samples by lane (all files):

1. T-47D
2. ZR-75-1
3. MDA-MB-453
4. MFM-223
5. Other samples not related to this manuscript
6. Other samples not related to this manuscript
7. Other samples not related to this manuscript

Fig 1F 2019-12-02 12hr 41min_Exp_4.0sec T47 ZR AR IP AR WB


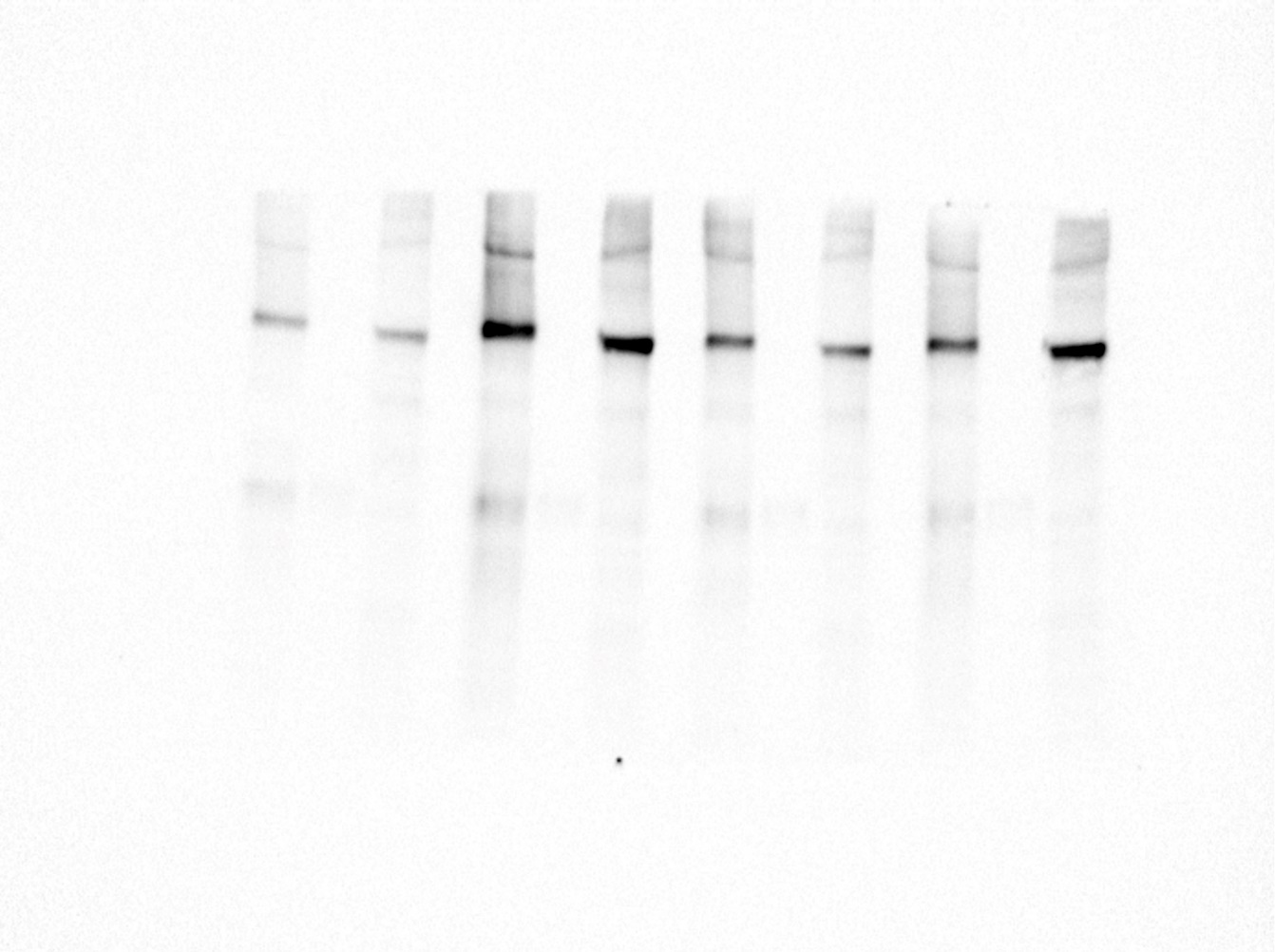


Fig 1F 2019-12-03 19hr 15min_Exposure_29.0sec_T47 ZR AR IP GATA3 WB


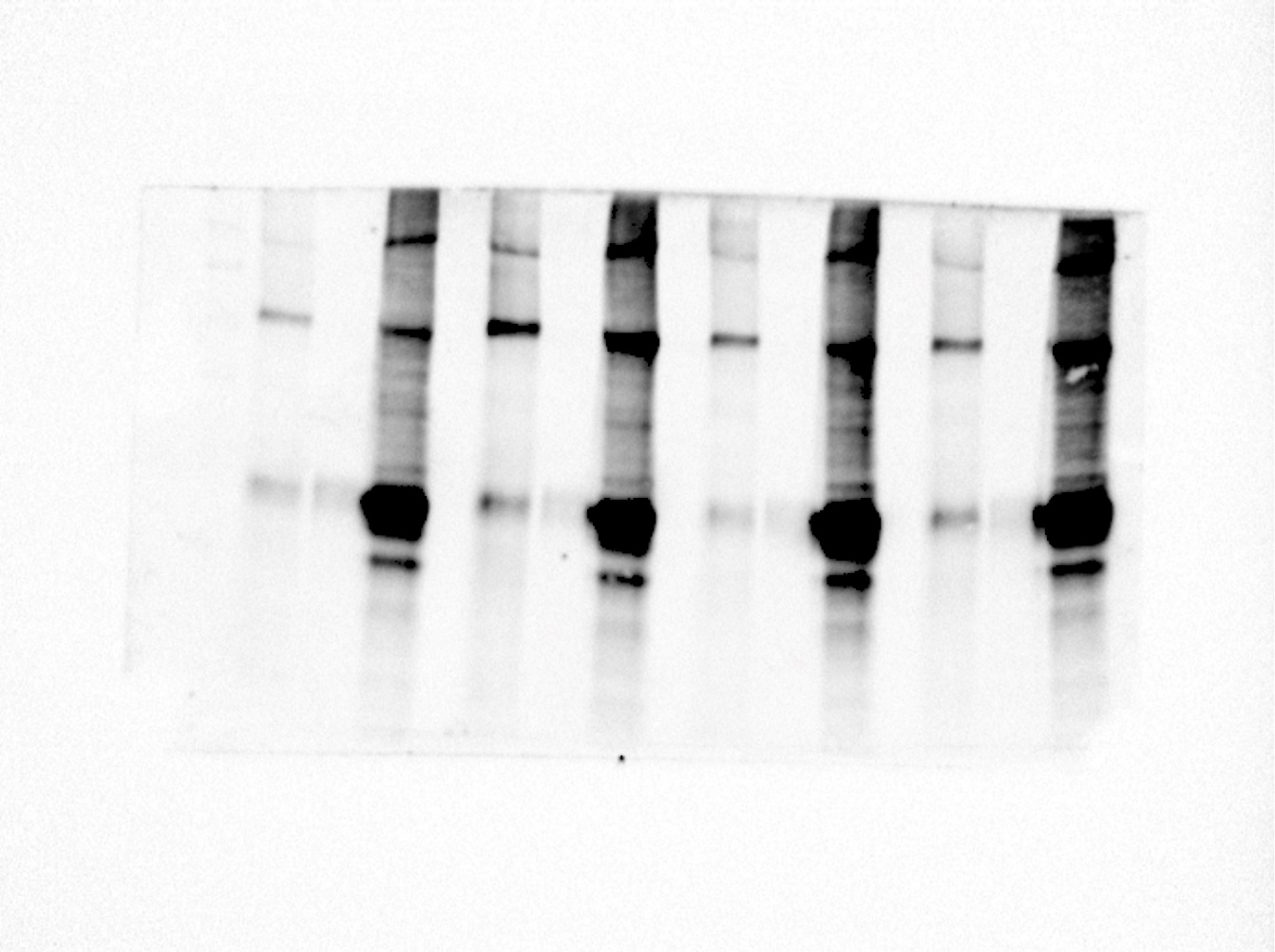


Fig1F AUTO 2 -Chemidoc 2020-04-21 13hr 07min 223 AR and GAT


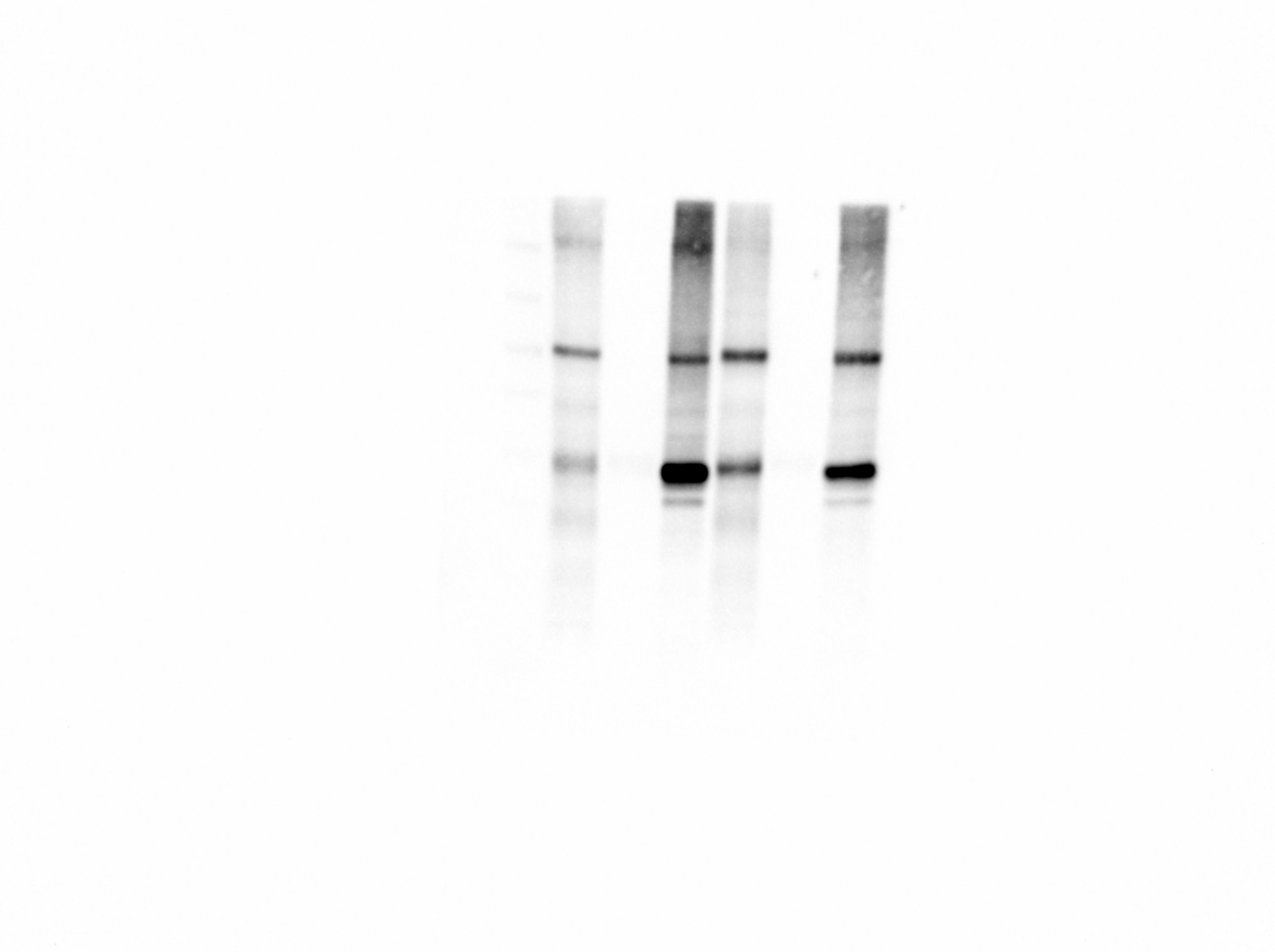


Fig 1 453 AR_Chemidoc 2019-12-02 13hr 06min_Exposure_10.0sec


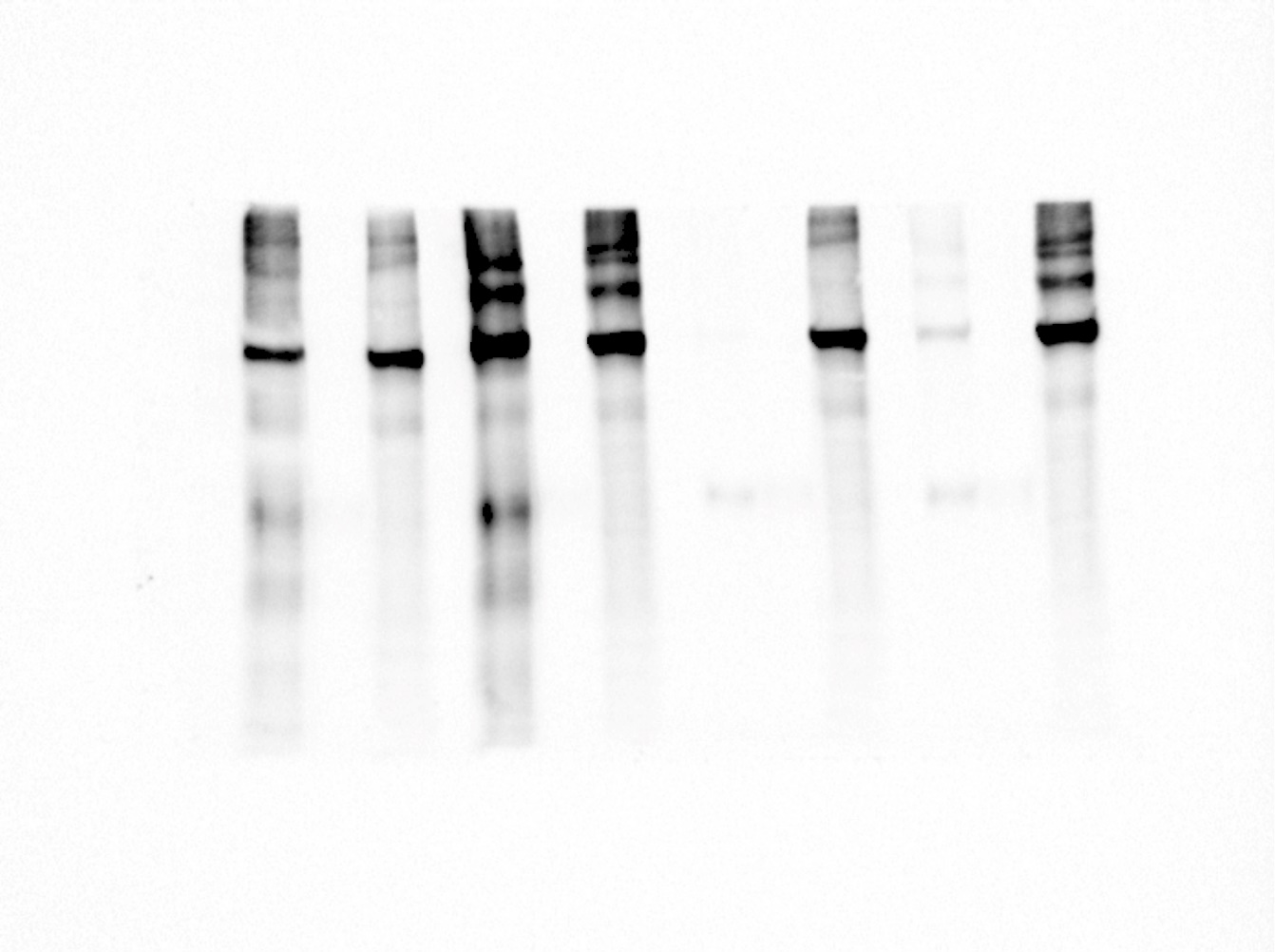


Fig 1 Fig S1 453-GATA BAND_Exposure_20.0sec


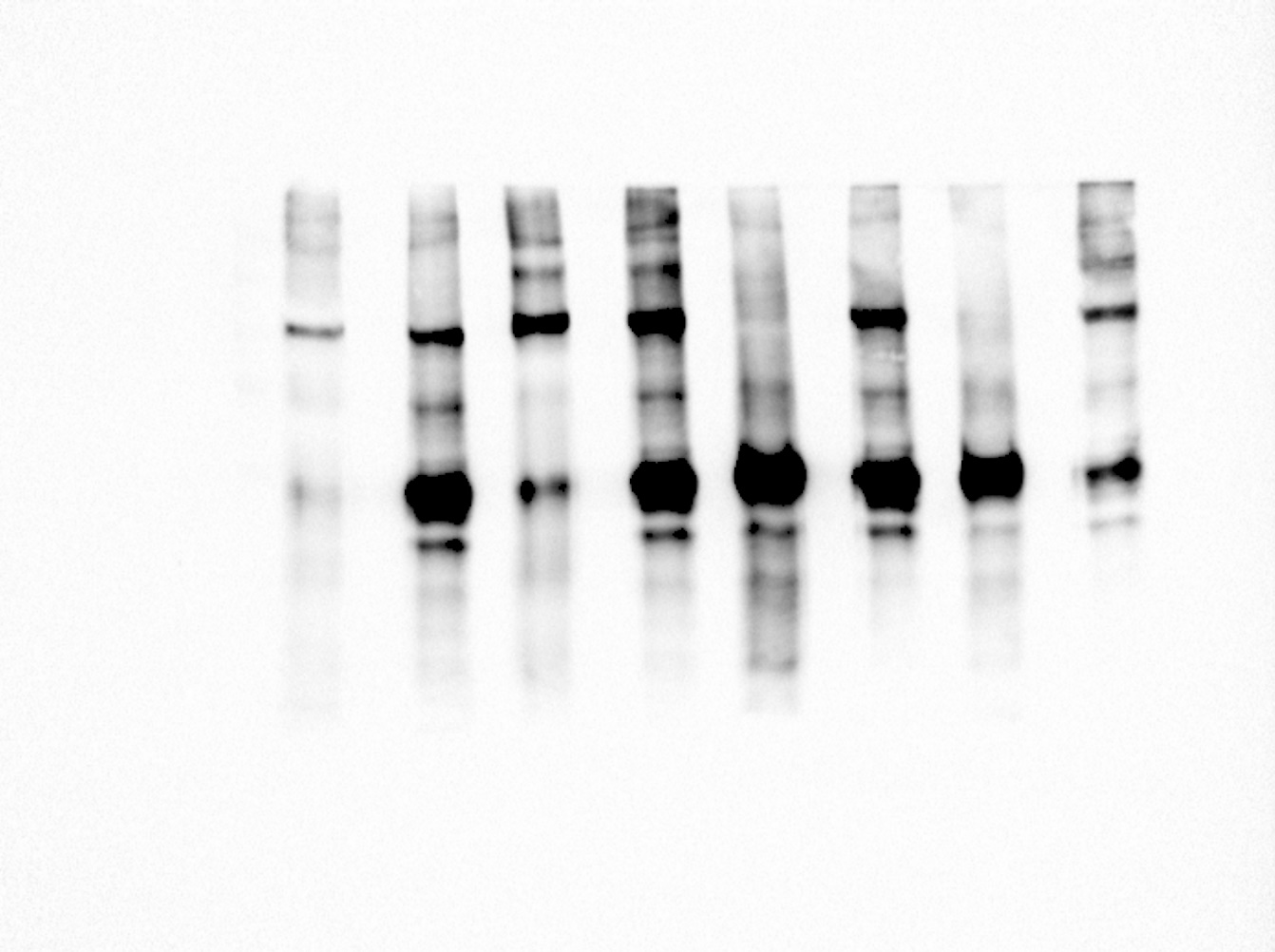


Fig S1E 2020-01-26 14hr 45min_Exp_18.0sec T47 AR


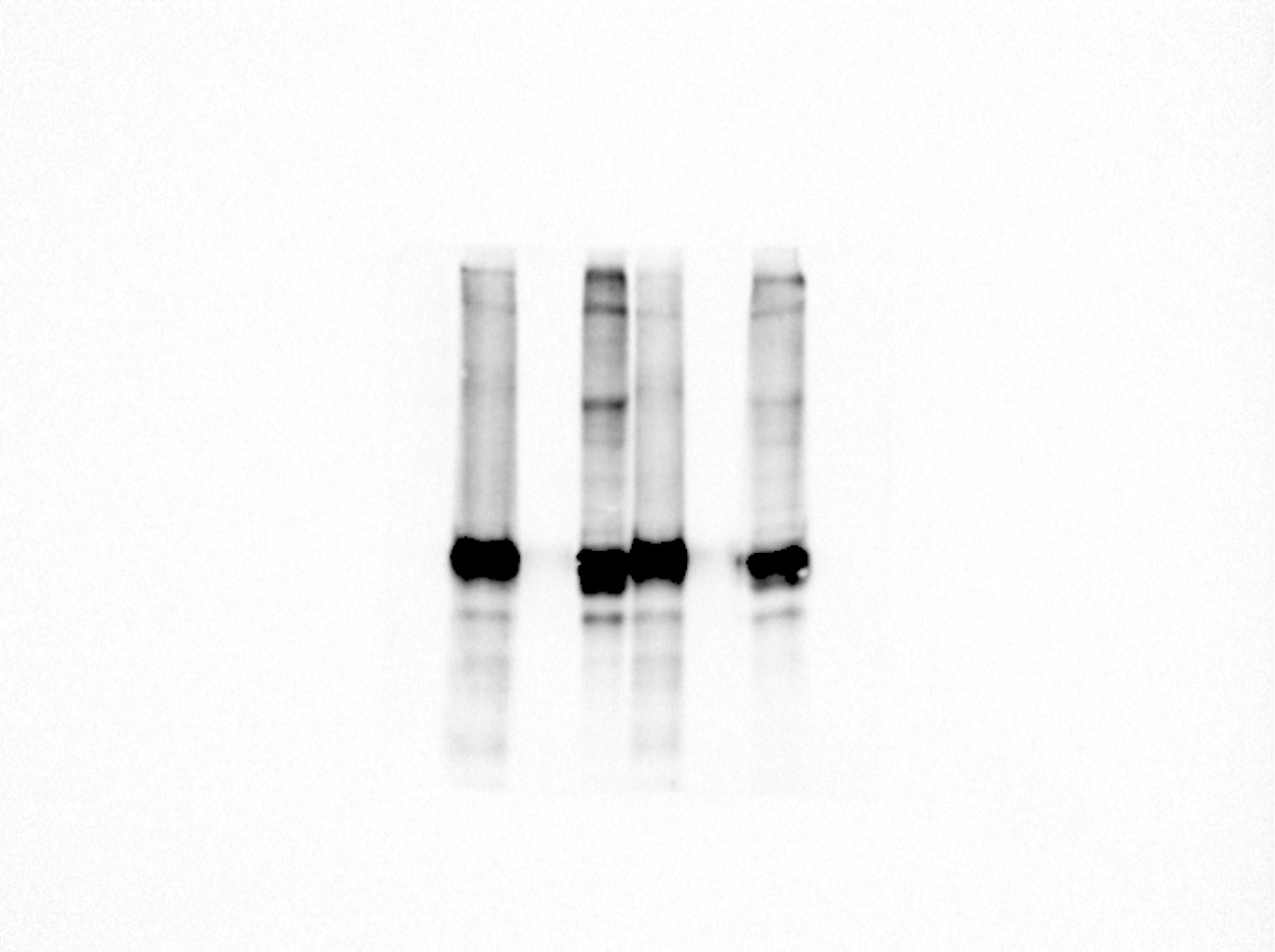


Fig S1E T47 GATA Chemidoc 2020-01-25 17hr 39min_Exp_20.0sec


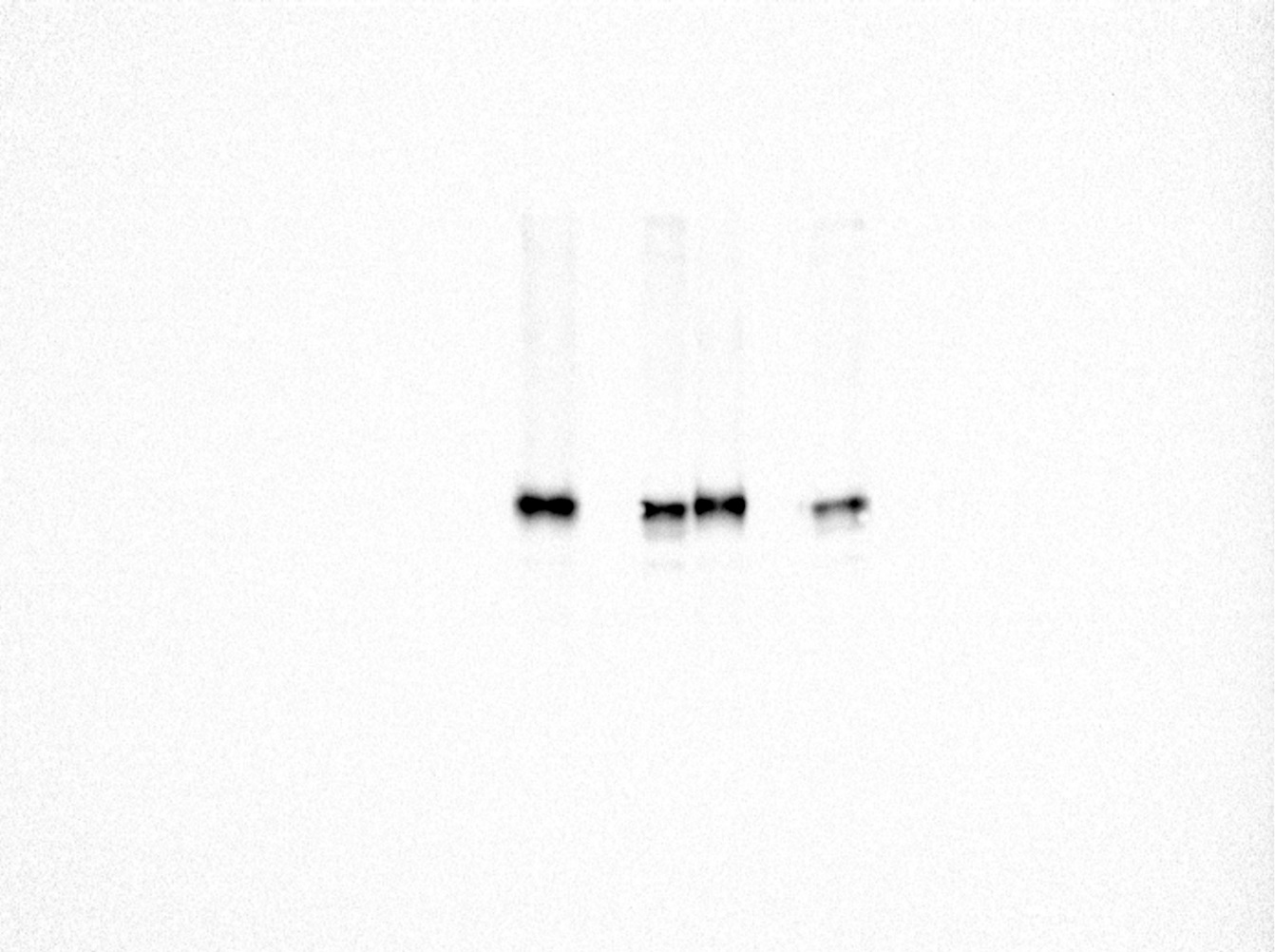


Fig S1E ZR AR Chemidoc 2020-01-26 14hr 35min_Exp_10.0sec


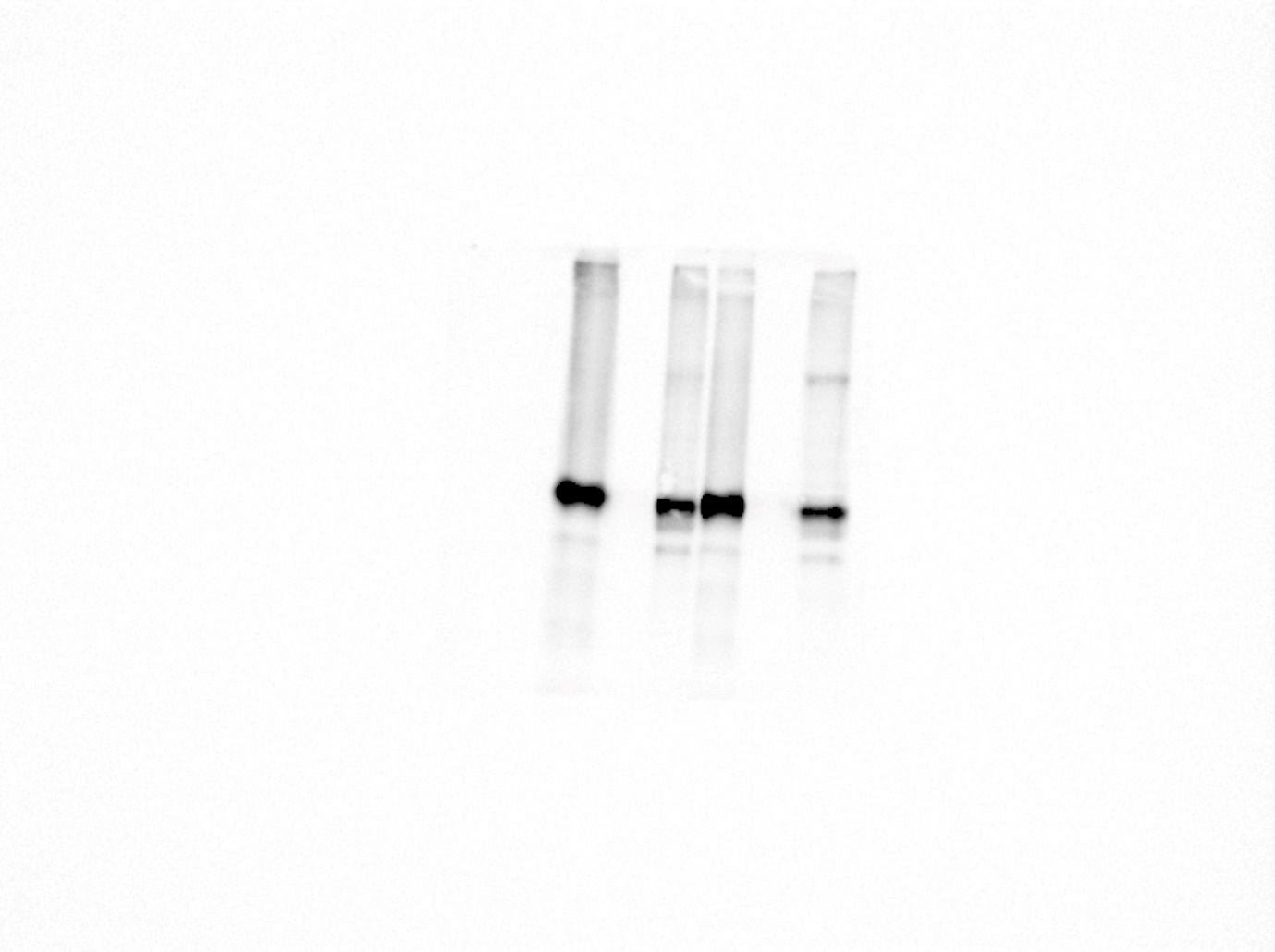


Fig S1E ZR GATA3 Chemidoc 2020-01-25 17hr 32min_Exp_29.0sec


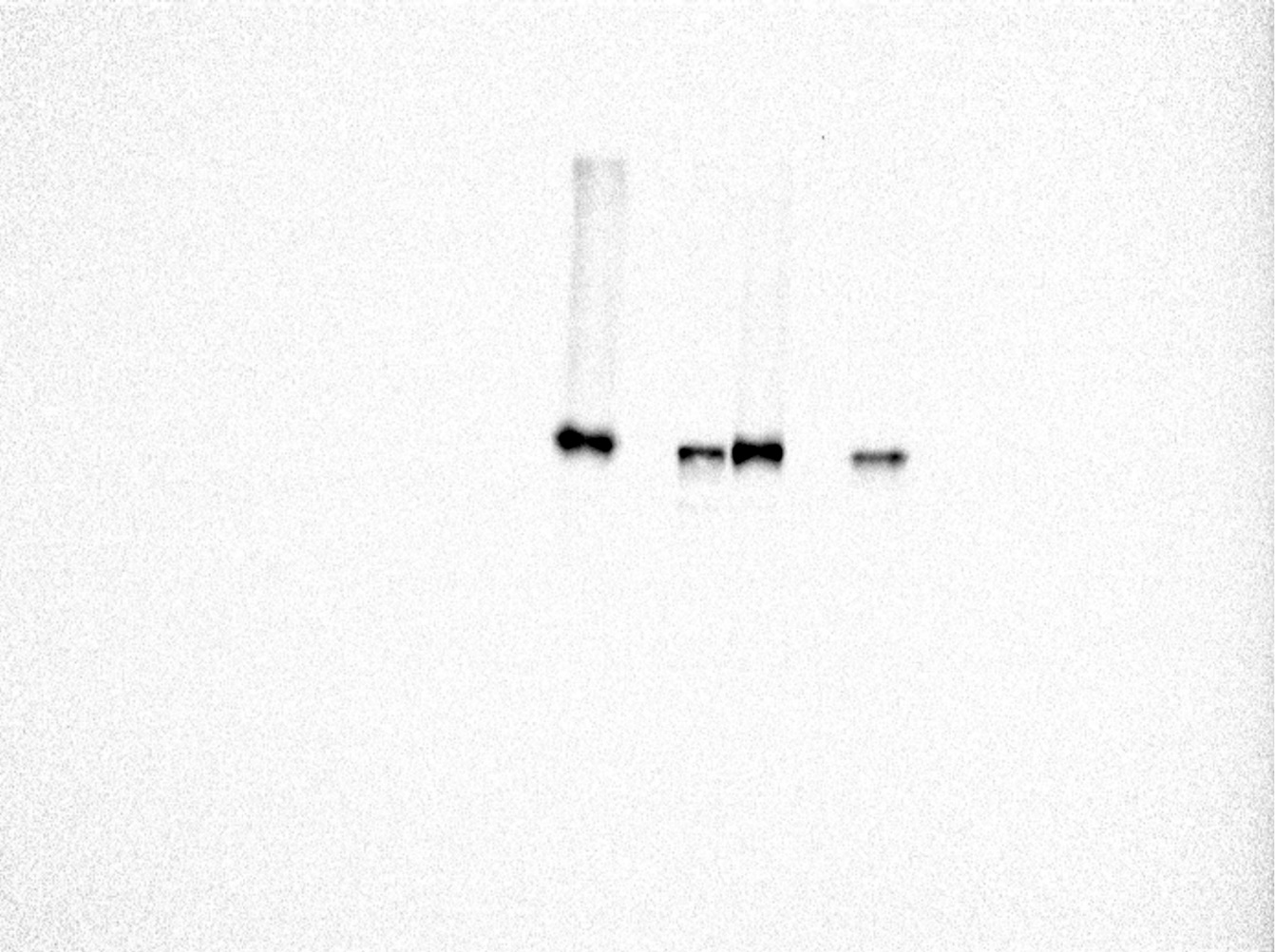


Fig S1E Auto-Chemidoc 2020-04-21 12hr 57min 223 AR


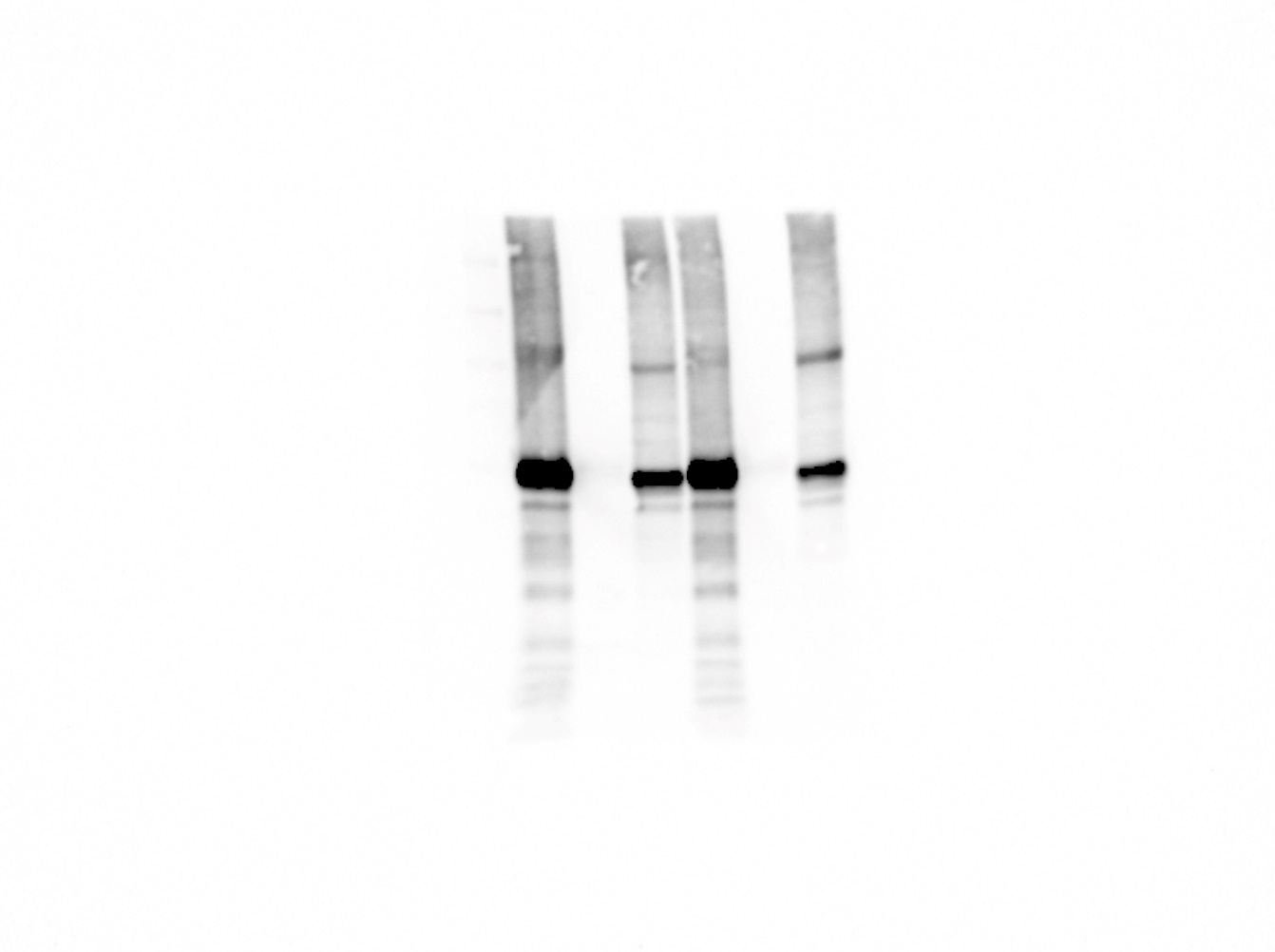


Fig S1E Auto - Chemidoc 2020-04-20 11hr 51min 223 GATA3


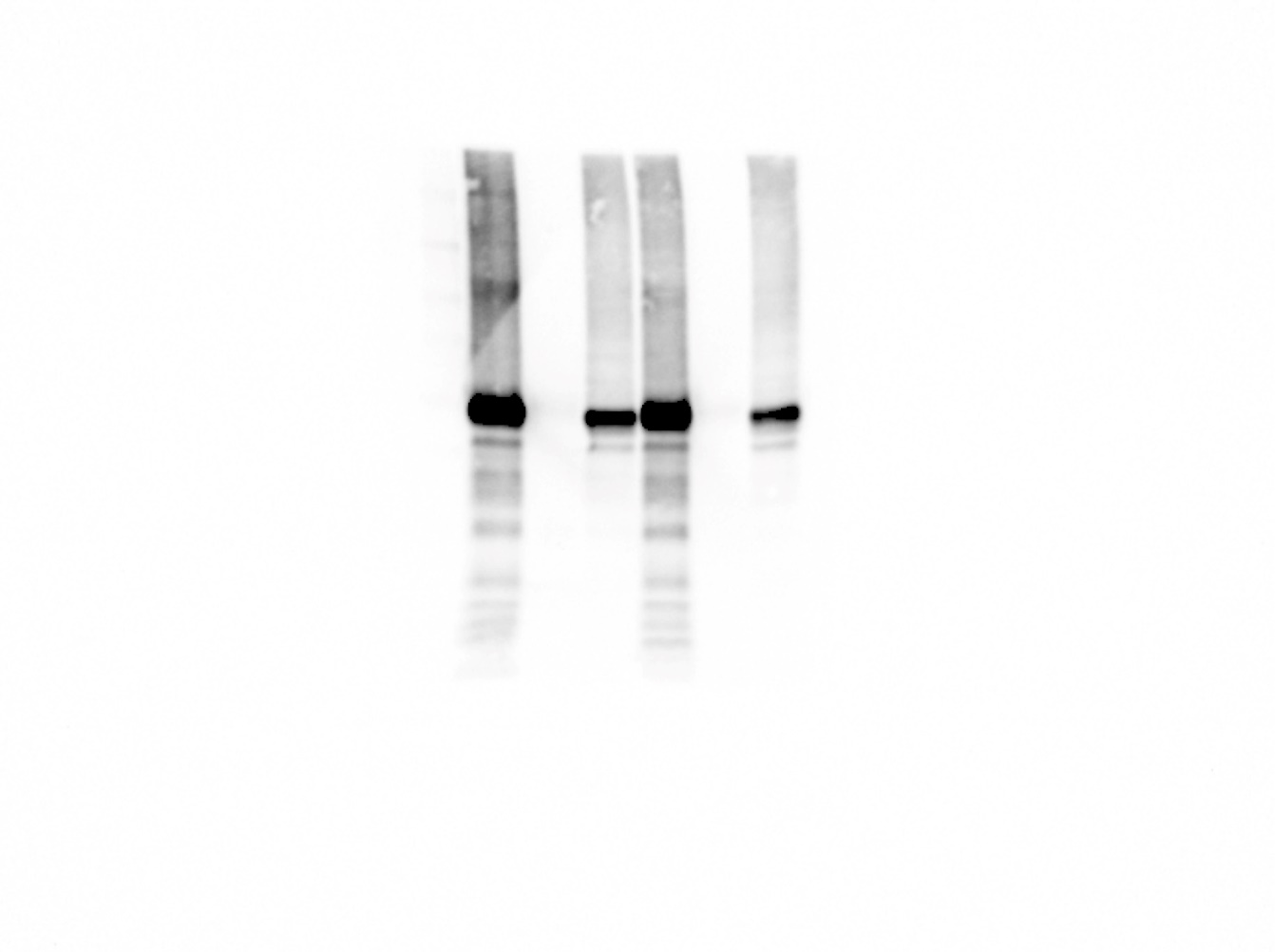


Fig S1 453 AR_Chemidoc 2019-12-02 13hr 06min_Exp_1.0sec
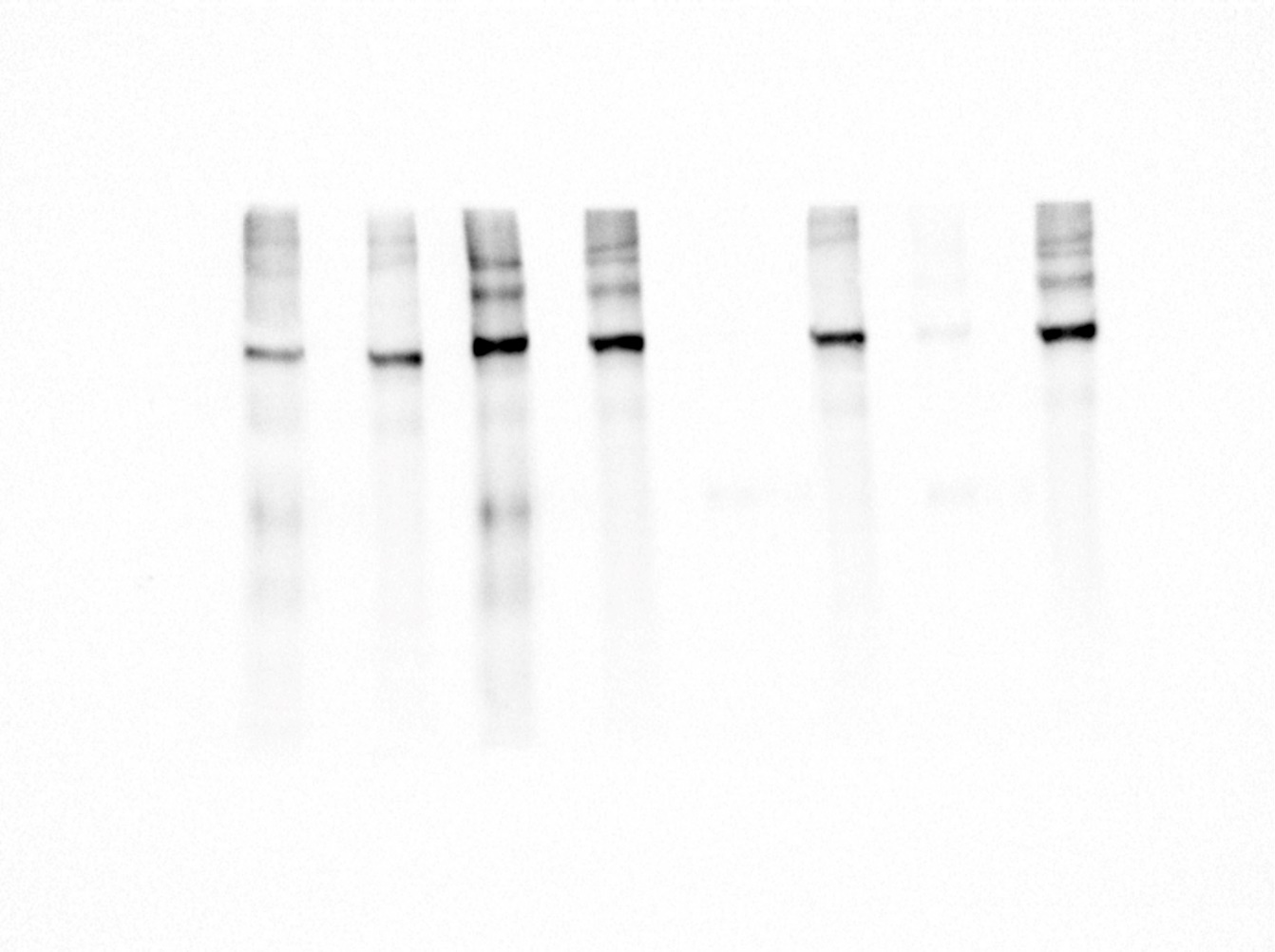


Fig S1F AR GATA3 Chemidoc 2019-02-03 14hr 02min_Exposure_7.4sec


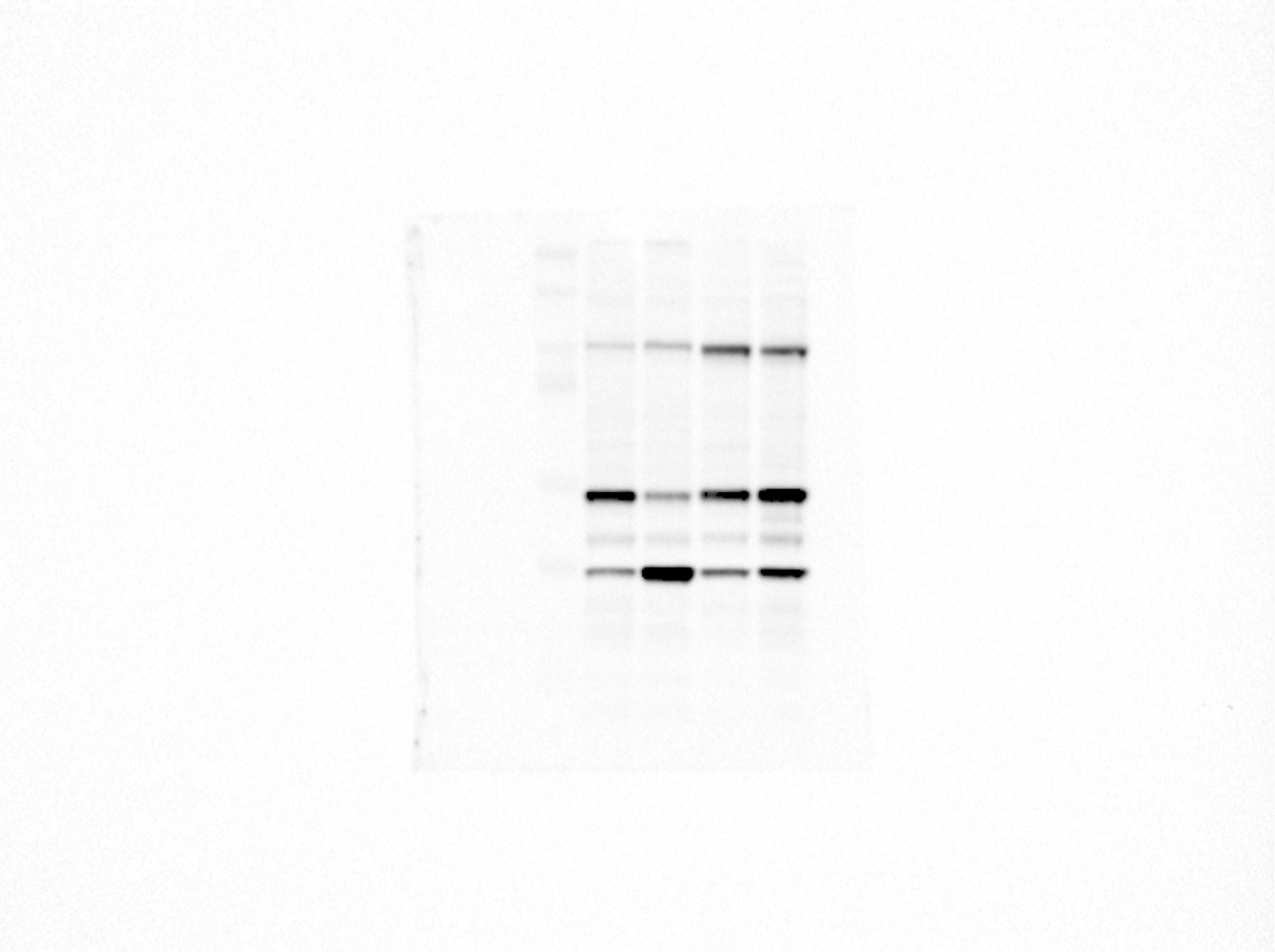


Fig S1F ER Chemidoc 2019-02-04 11hr 50min_Exposure_19.3sec


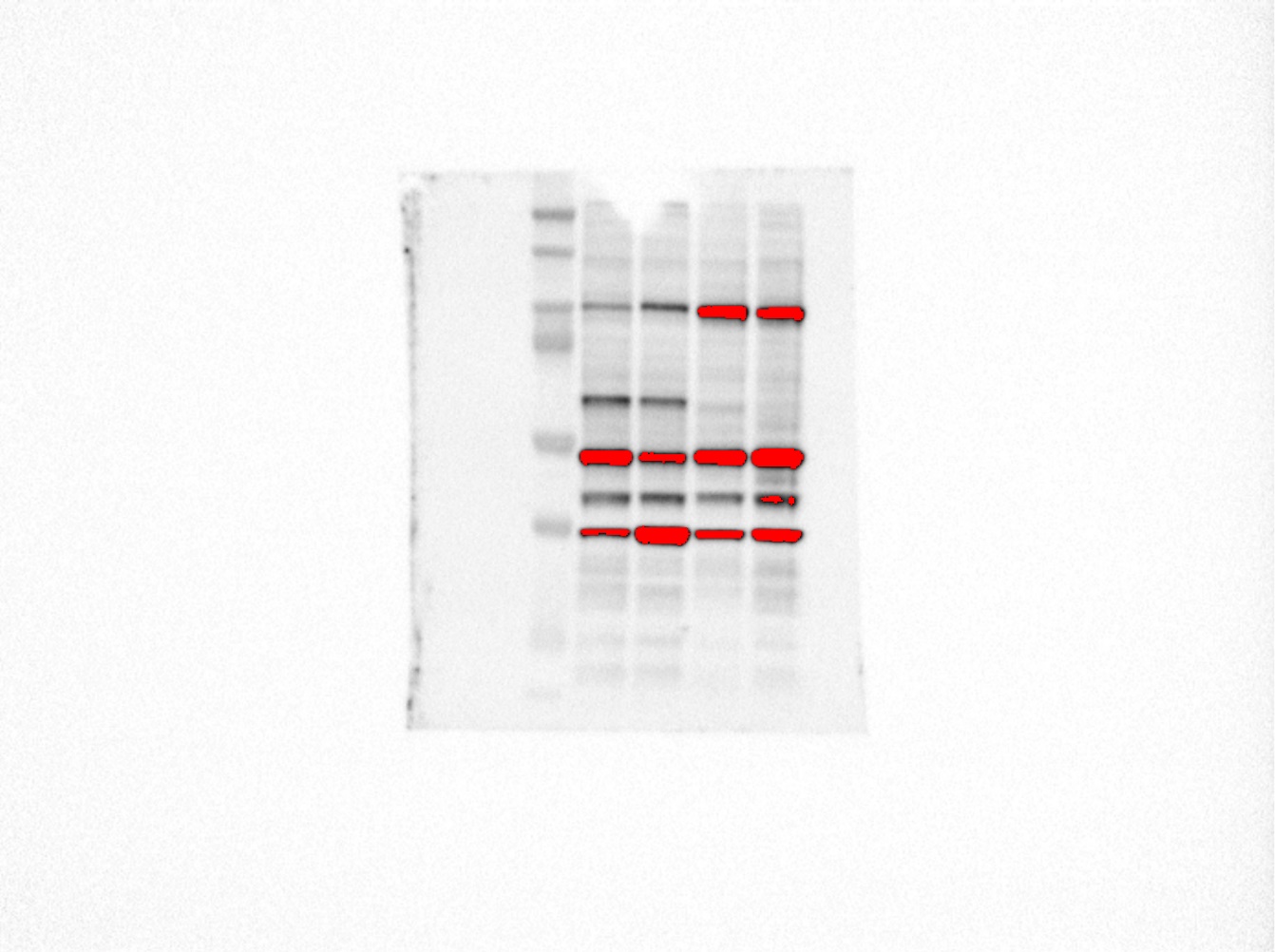


Fig S1F actin Chemidoc 2019-02-06 12hr 45min


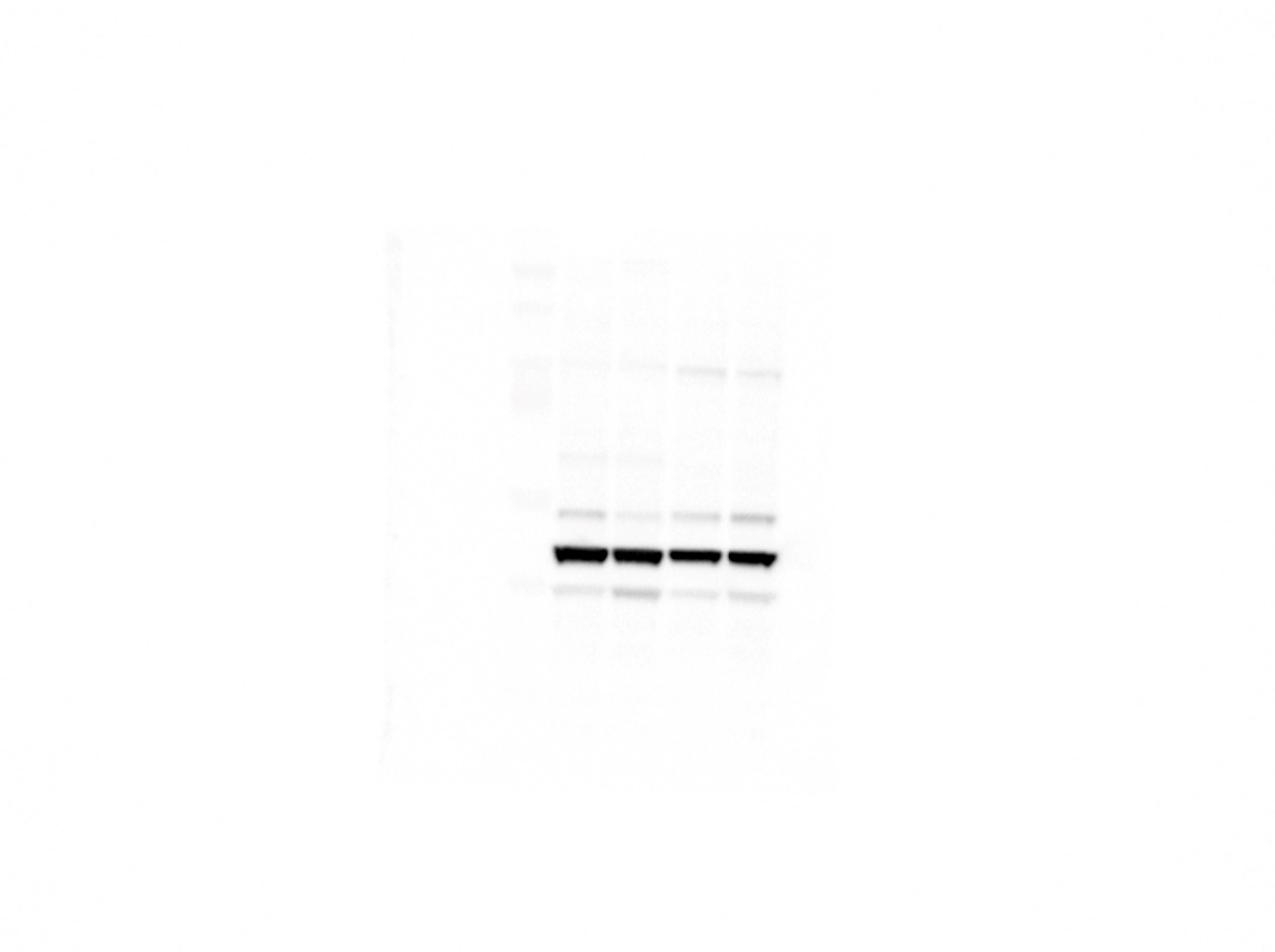


Fig S6 T47D-siAR-Chemidoc 2021-01-25 14hr 42min_Exposure_7.0sec


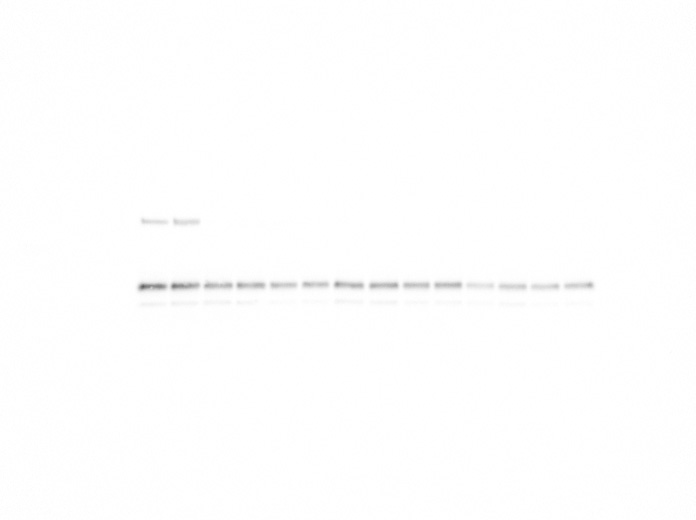


Fig S6 T47D-GAPDH-siAR top- siGATA3 bottom-Exposure_3.0sec


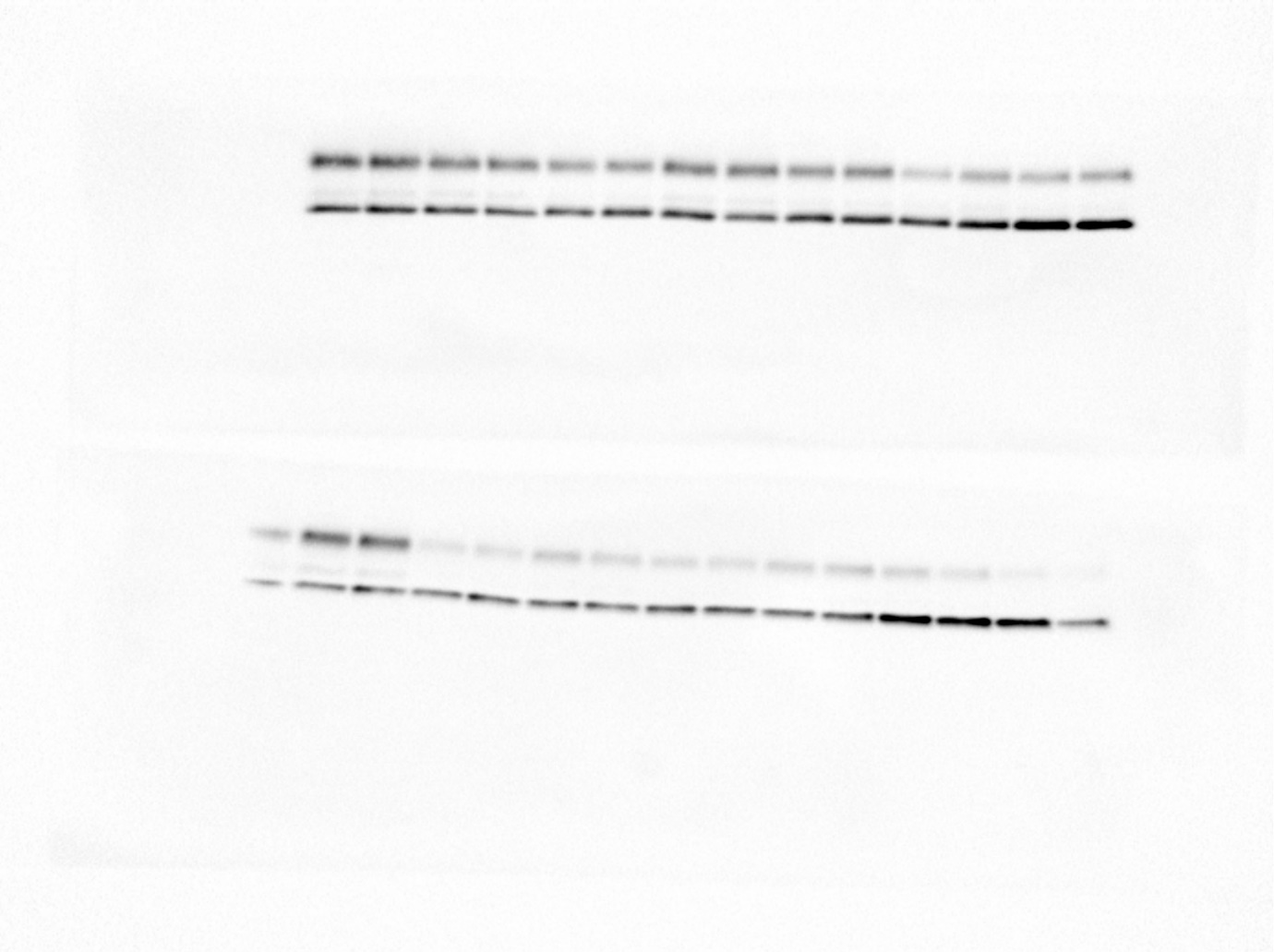


Fig S6 453-siAR-AR


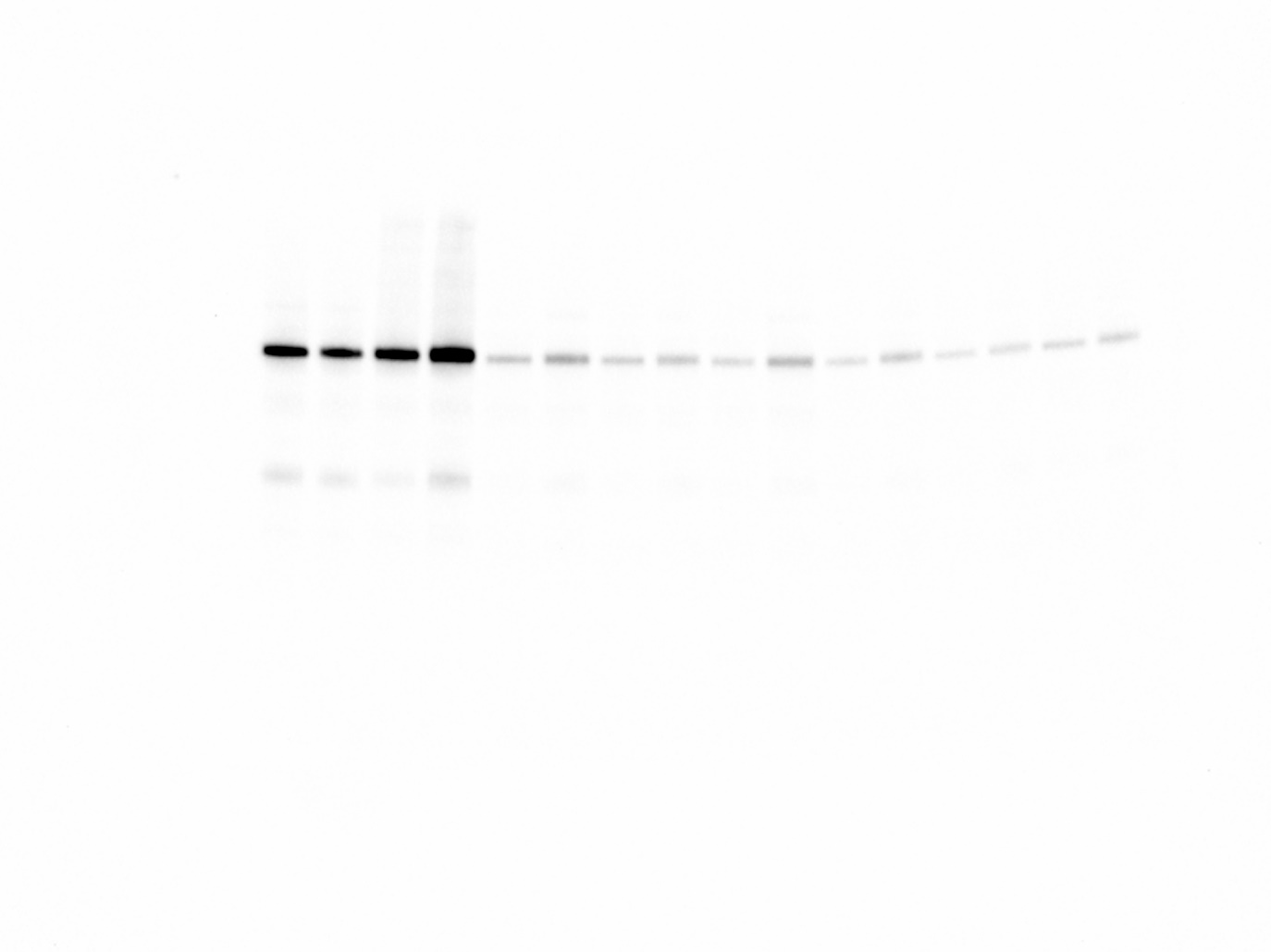


Fig S6 453-siAR-GATA3


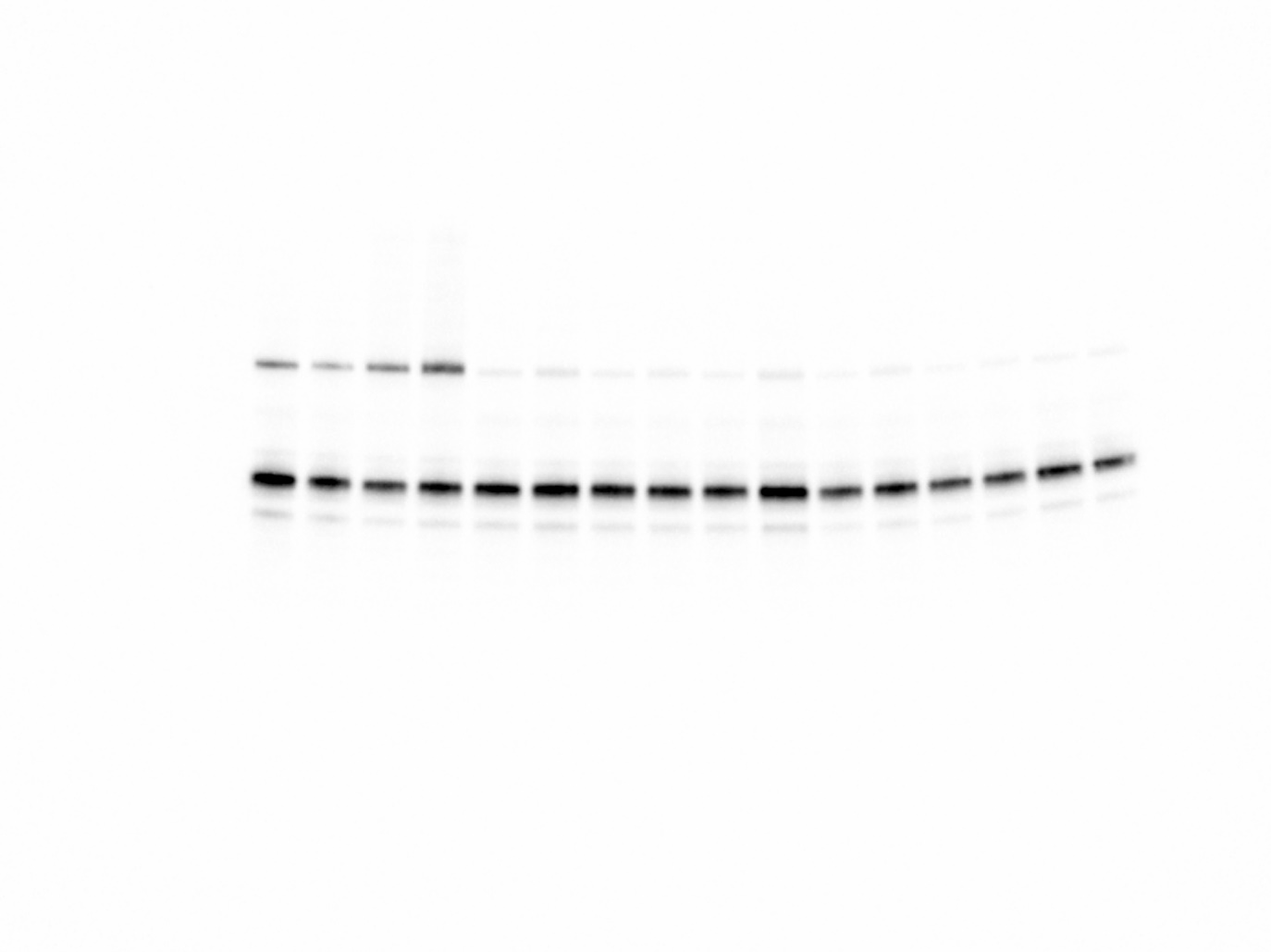


Fig S6 453-siAR-stainfreegel


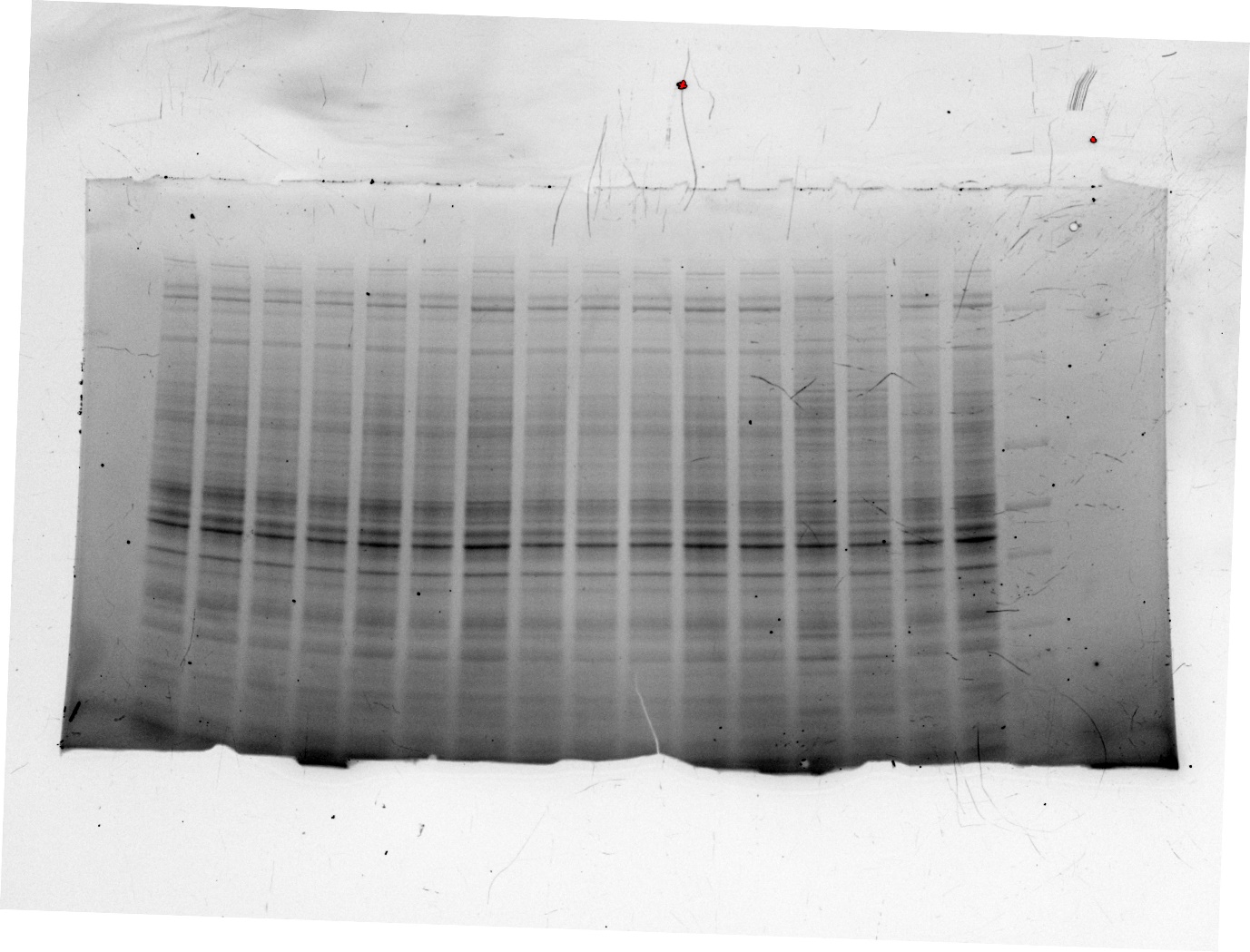


Fig S6 T47D-siGATA3-Chemidoc 2021-01-25 14hr 53min_Exposure_10.1sec


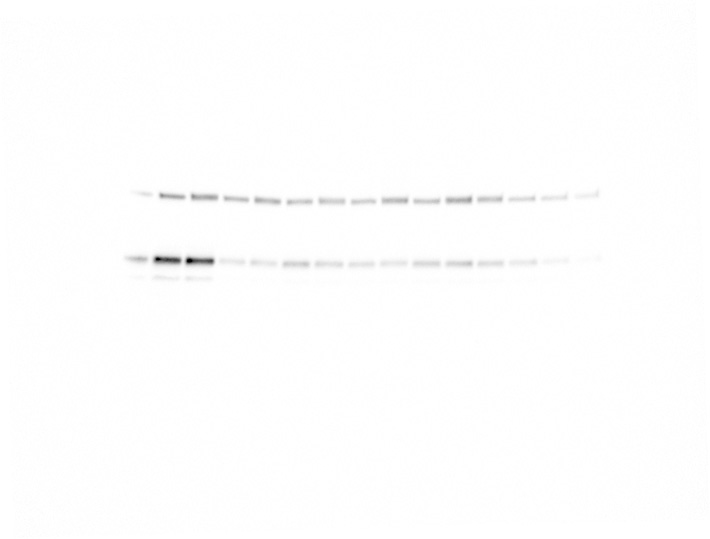


Fig S6 453 siGATA3 AR GAT 2021-01-25 15hr 00min_Exp_10.1sec


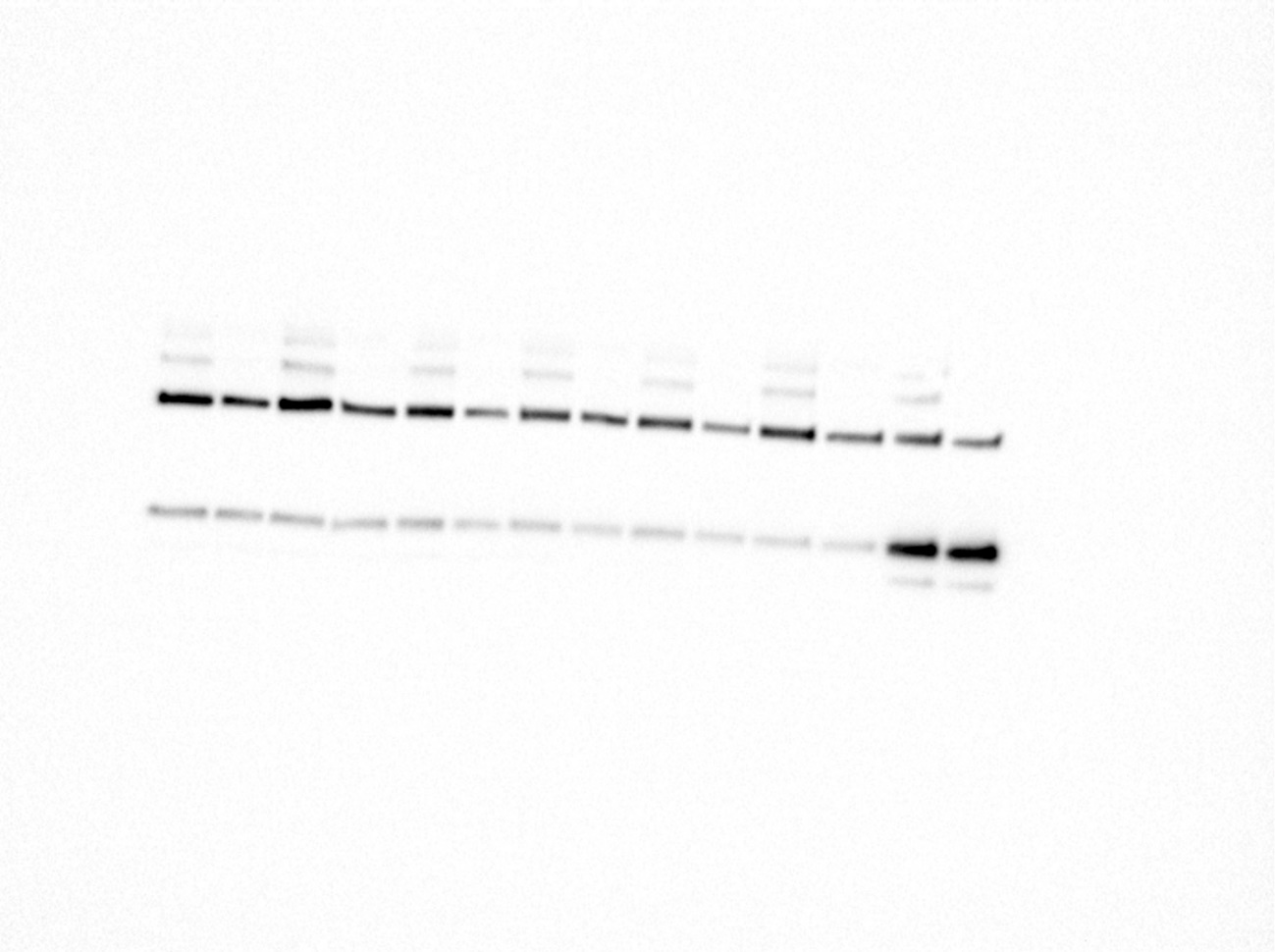


Fig S6 453 siGATA3 GAPDH 2021-01-25 17hr 29min_Exp_2.0sec


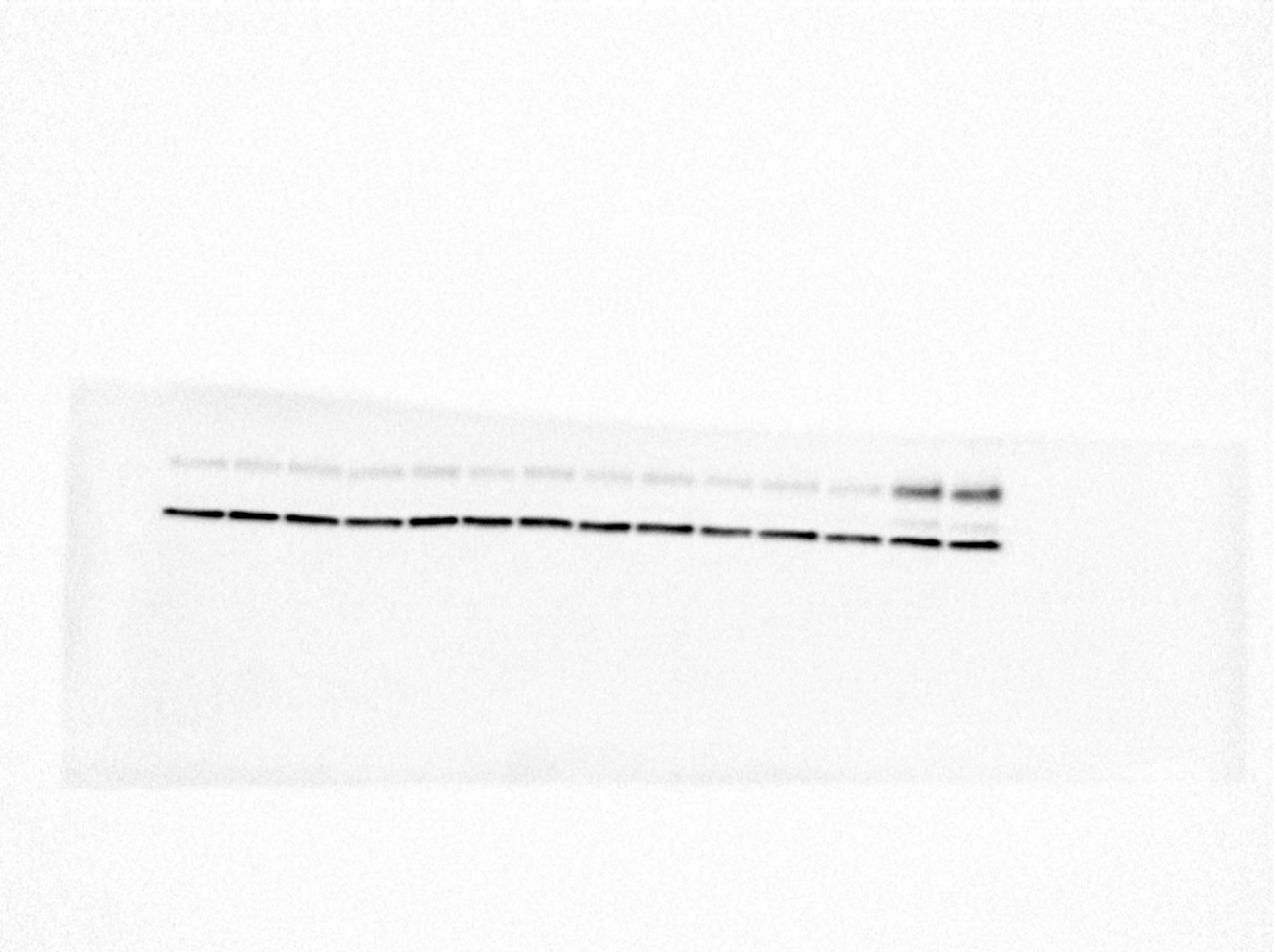


Fig S7 GLL372 GLL373 T-47D AR 3 sec


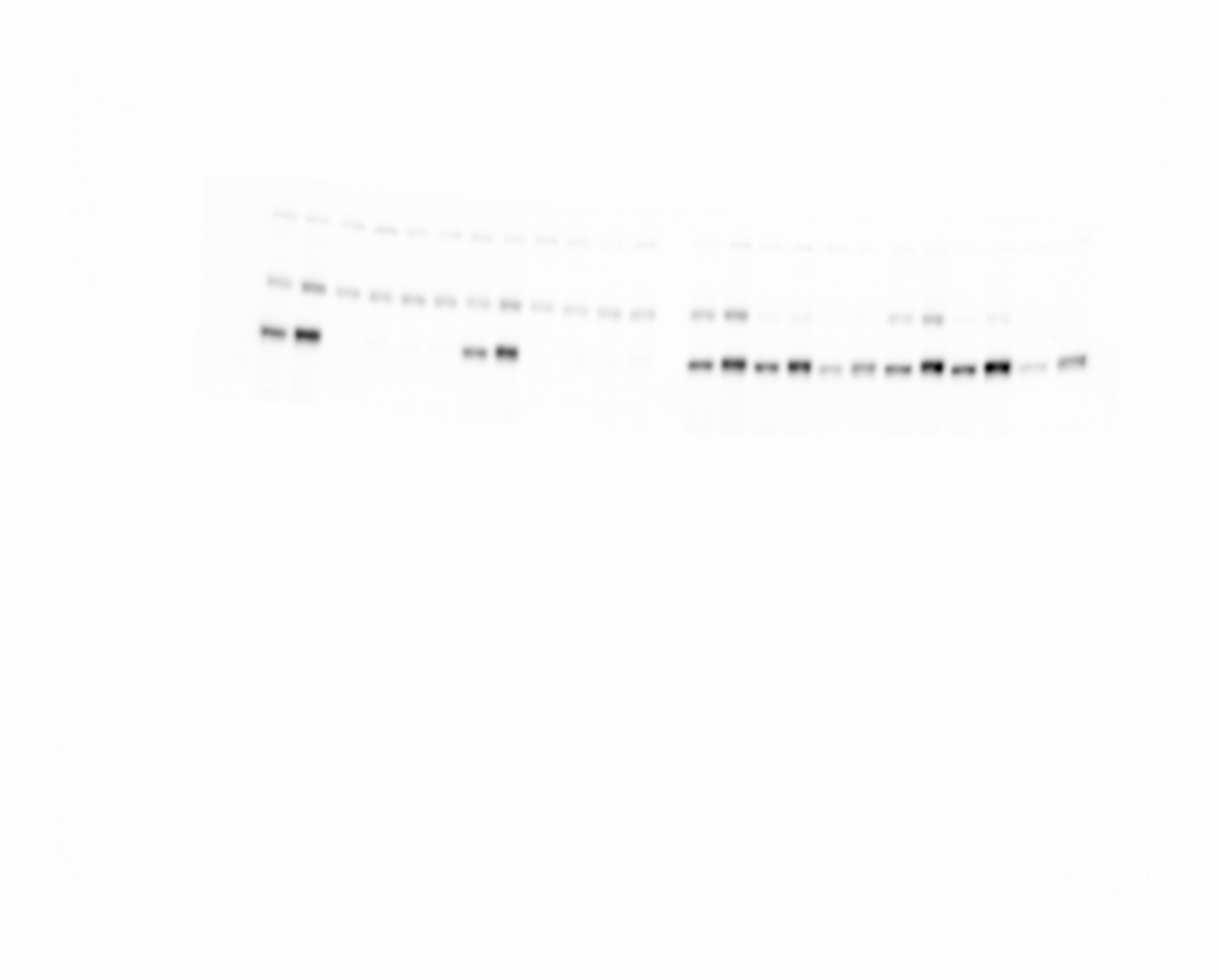


Fig S7 GLL372 GLL373 T-47D KDM4B blot 10 sec


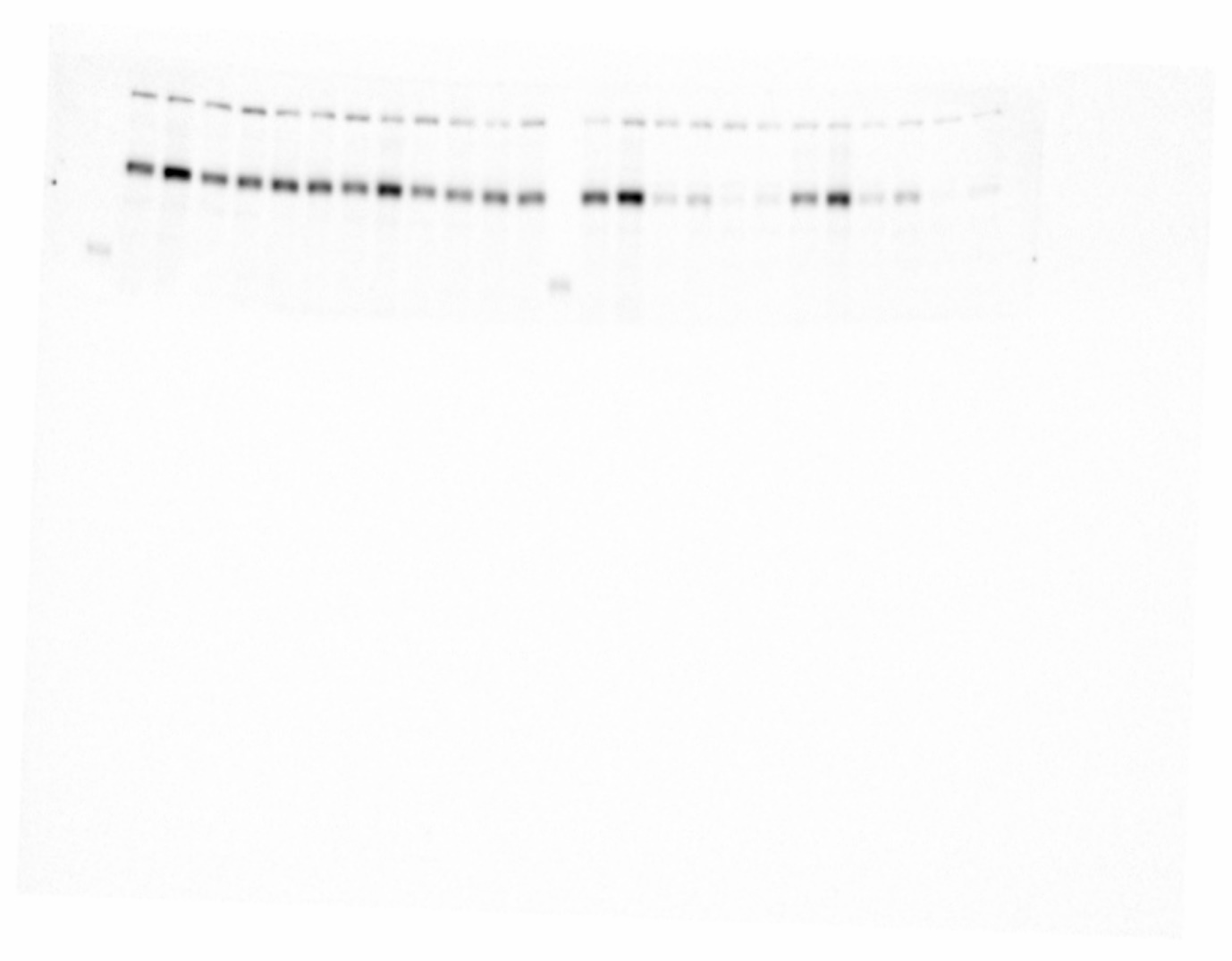


Fig S7 GLL372 GLL373 T-47D gel


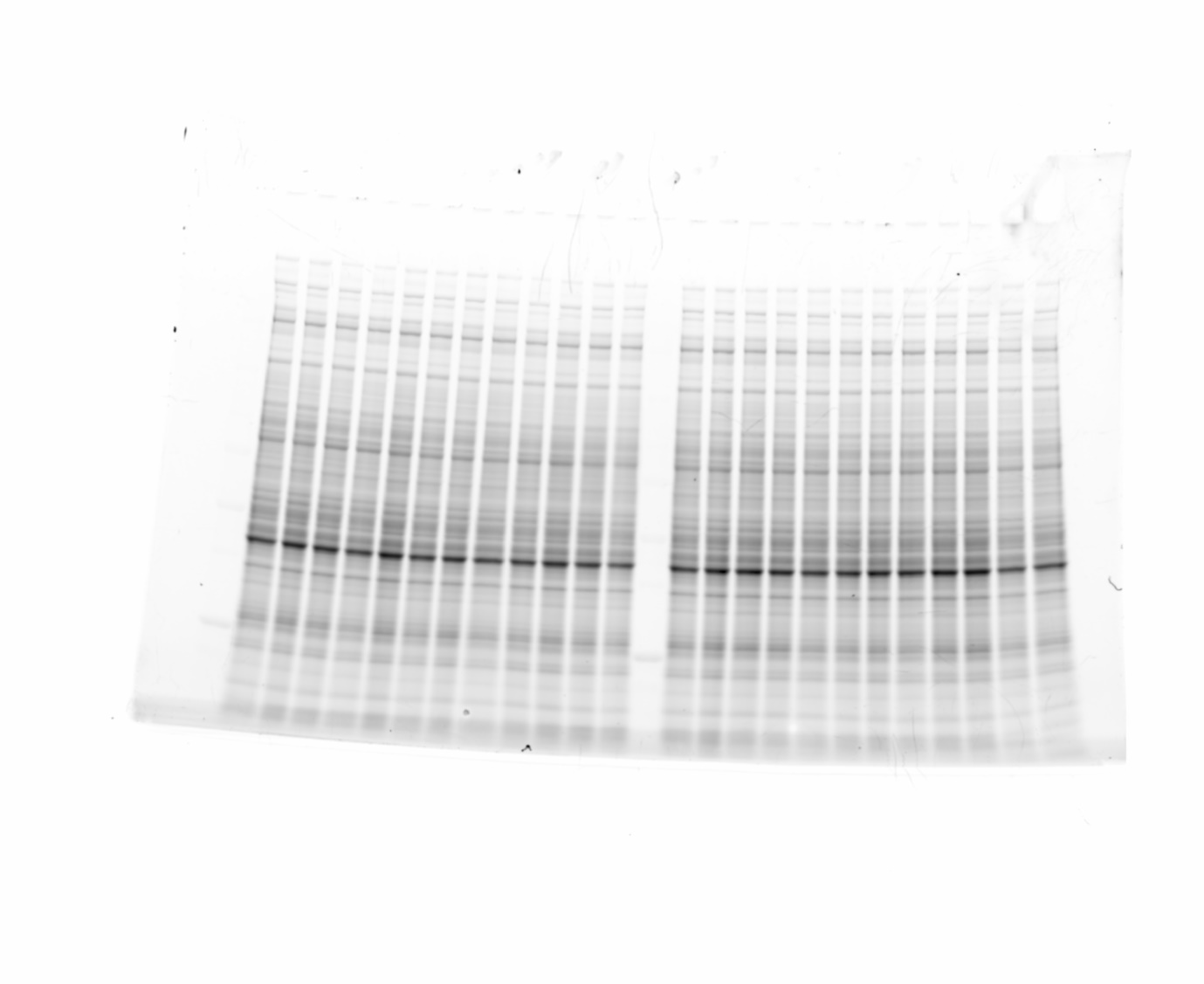


Fig S7 GLL372 GLL373 MDA-MB-453 siAR AR


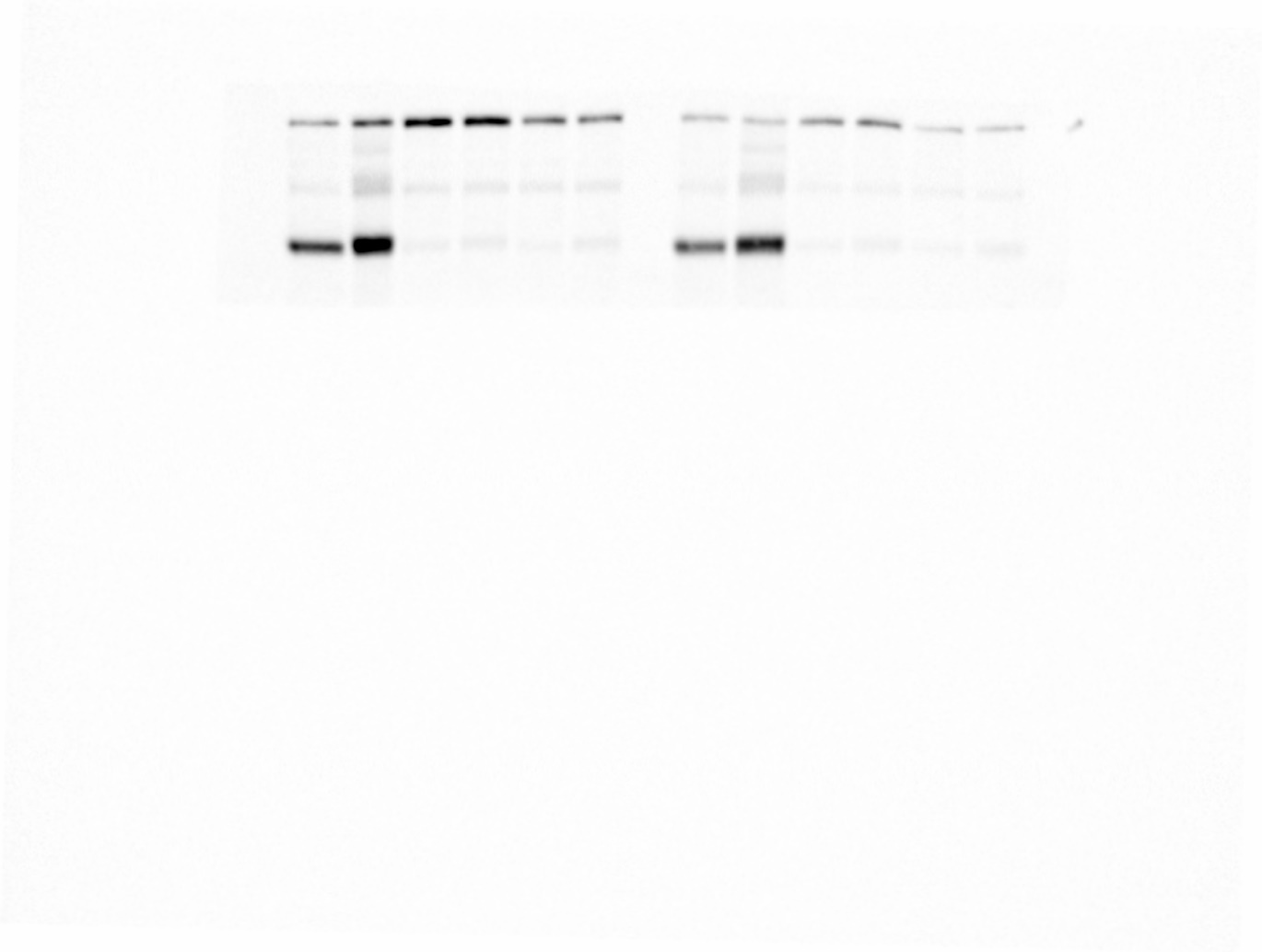


Fig S7 GLL372 GLL373 MDA-MB-453 siAR KDM4B 10 sec


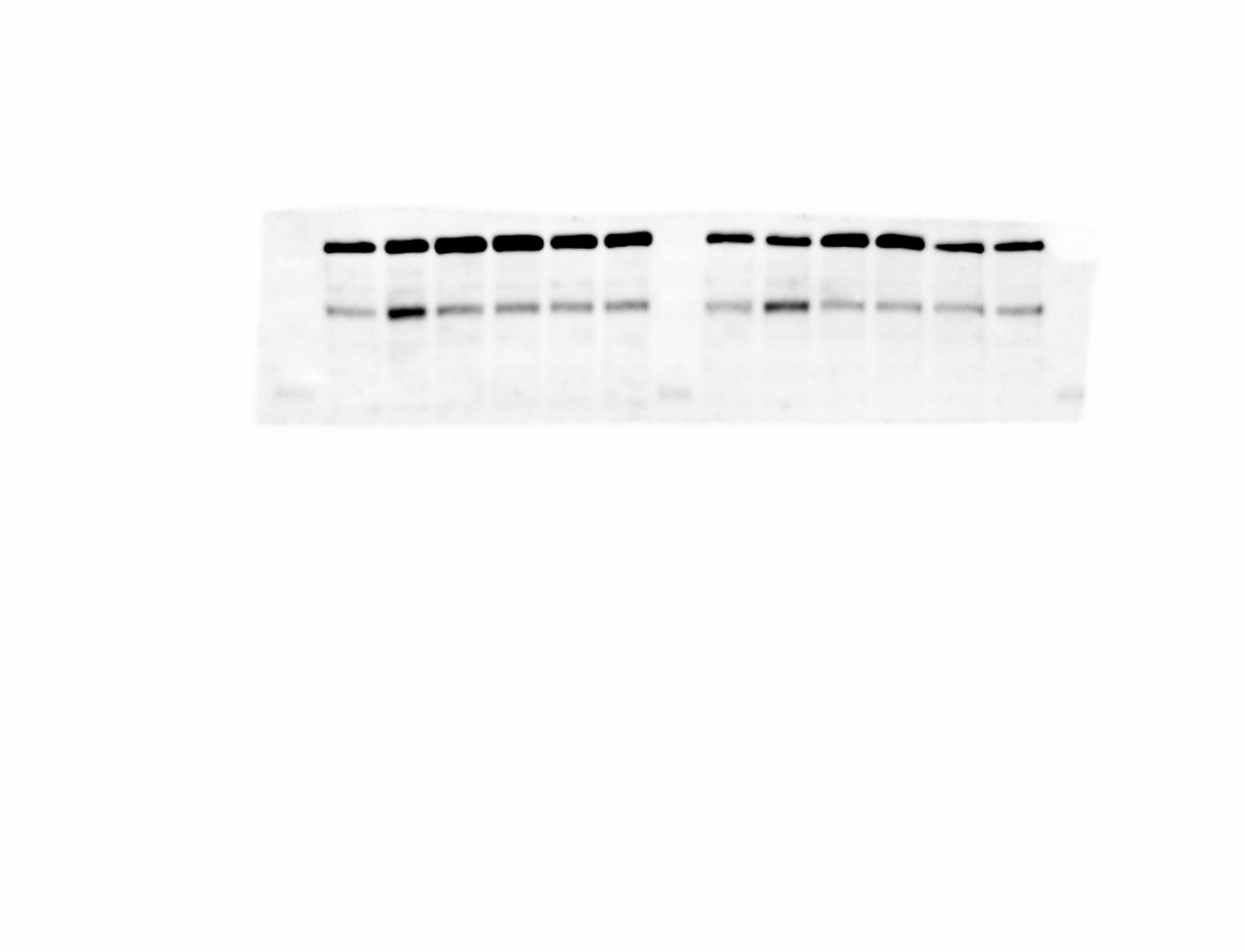


Fig S7 GLL372 GLL373 MDA-MB-453 siAR gel


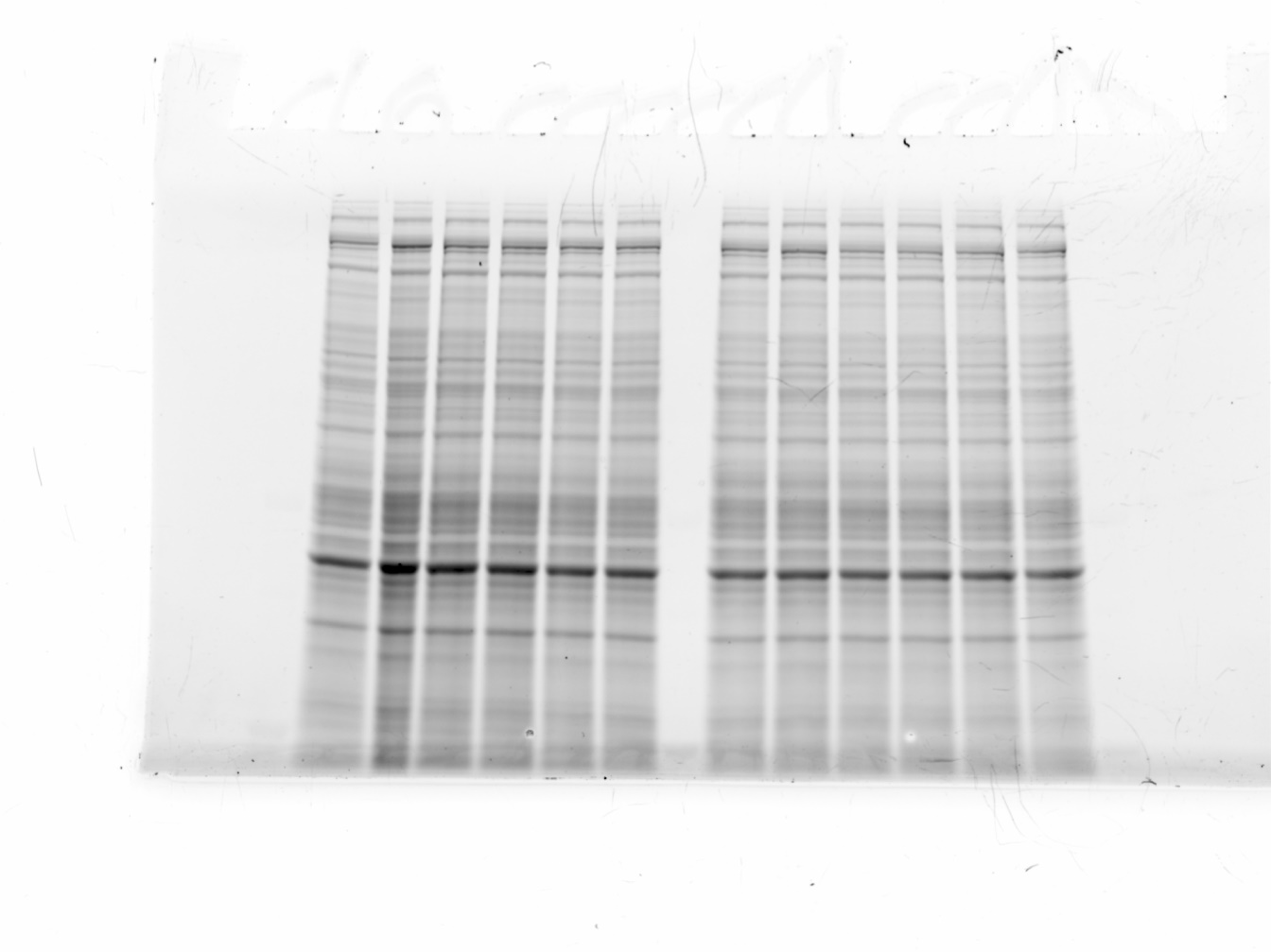


Fig S8 FOXA1_3seconds_20221214 CP


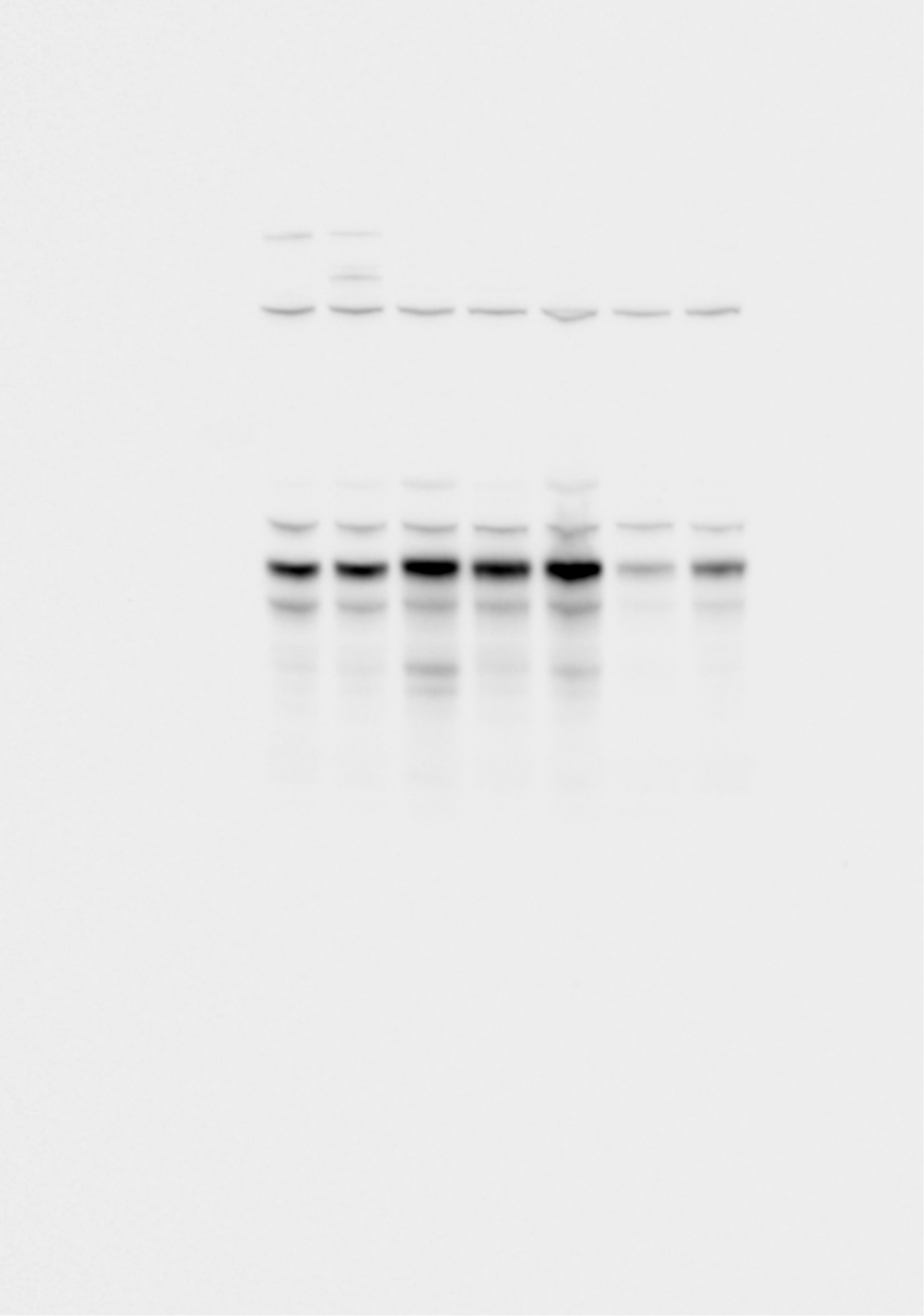


Fig S8 AR_11seconds_20221214 CP


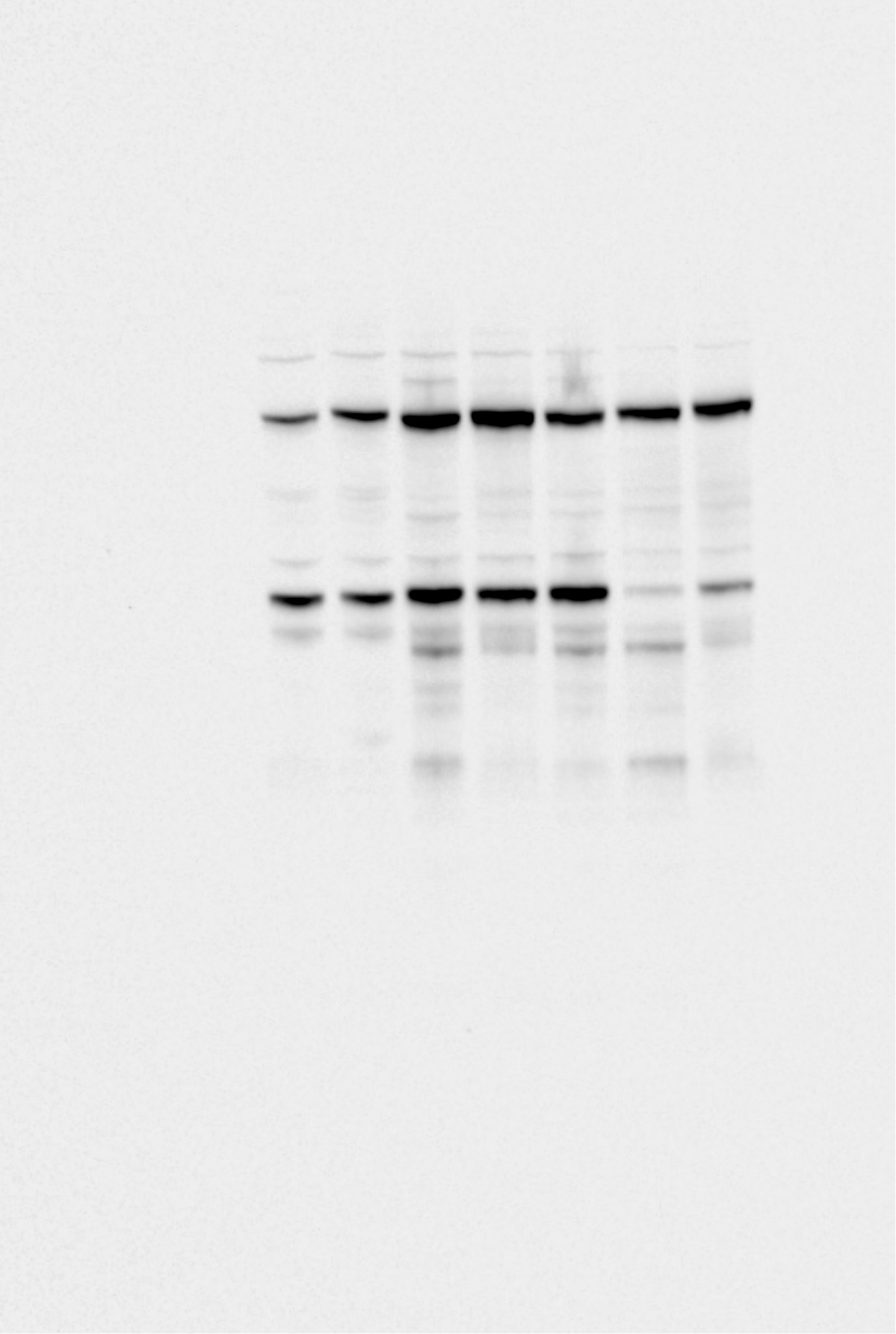


Fig S8 GATA3_7seconds_20221214 CP


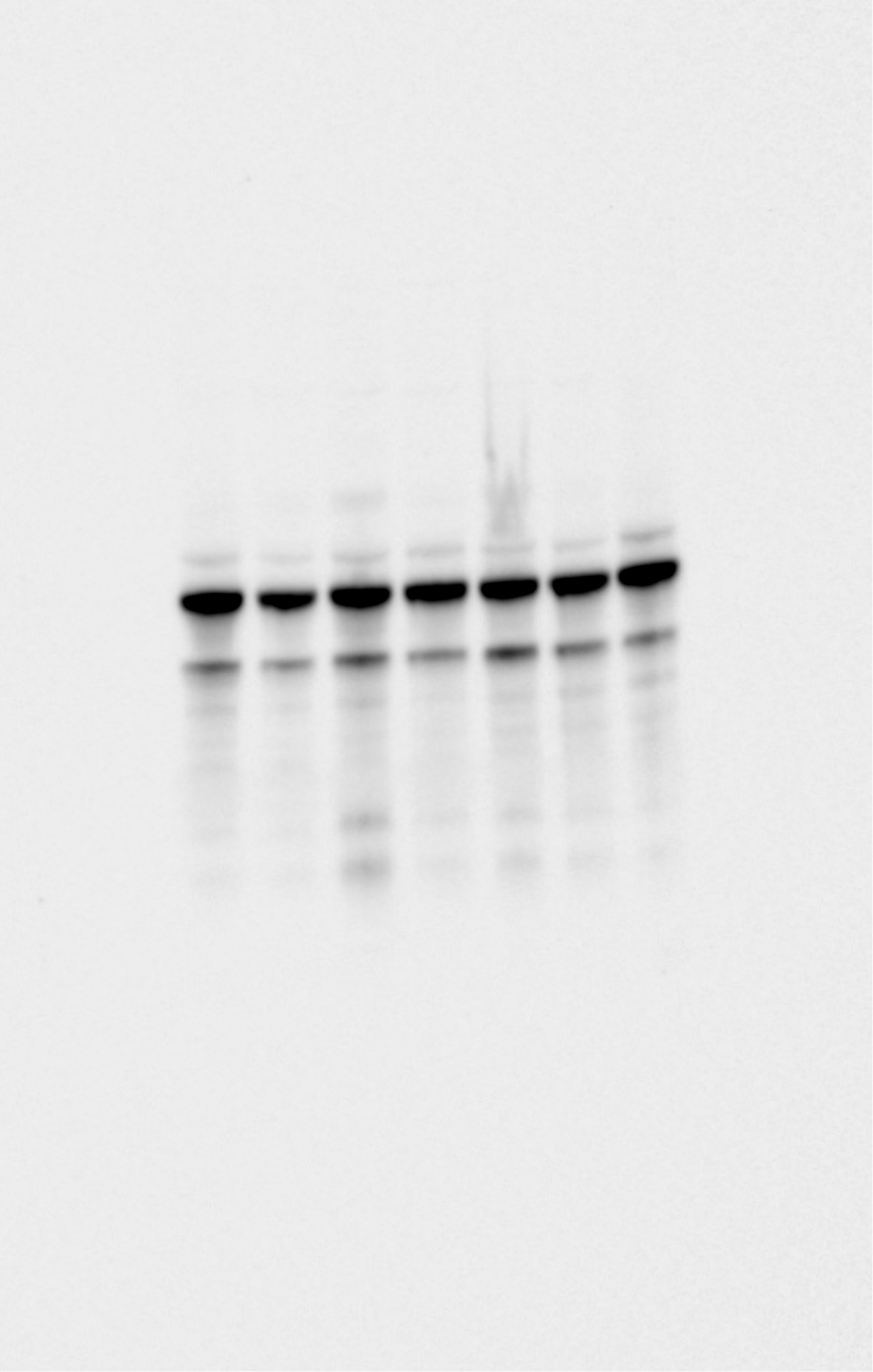


Fig S8 ER_38seconds_20221214 CP


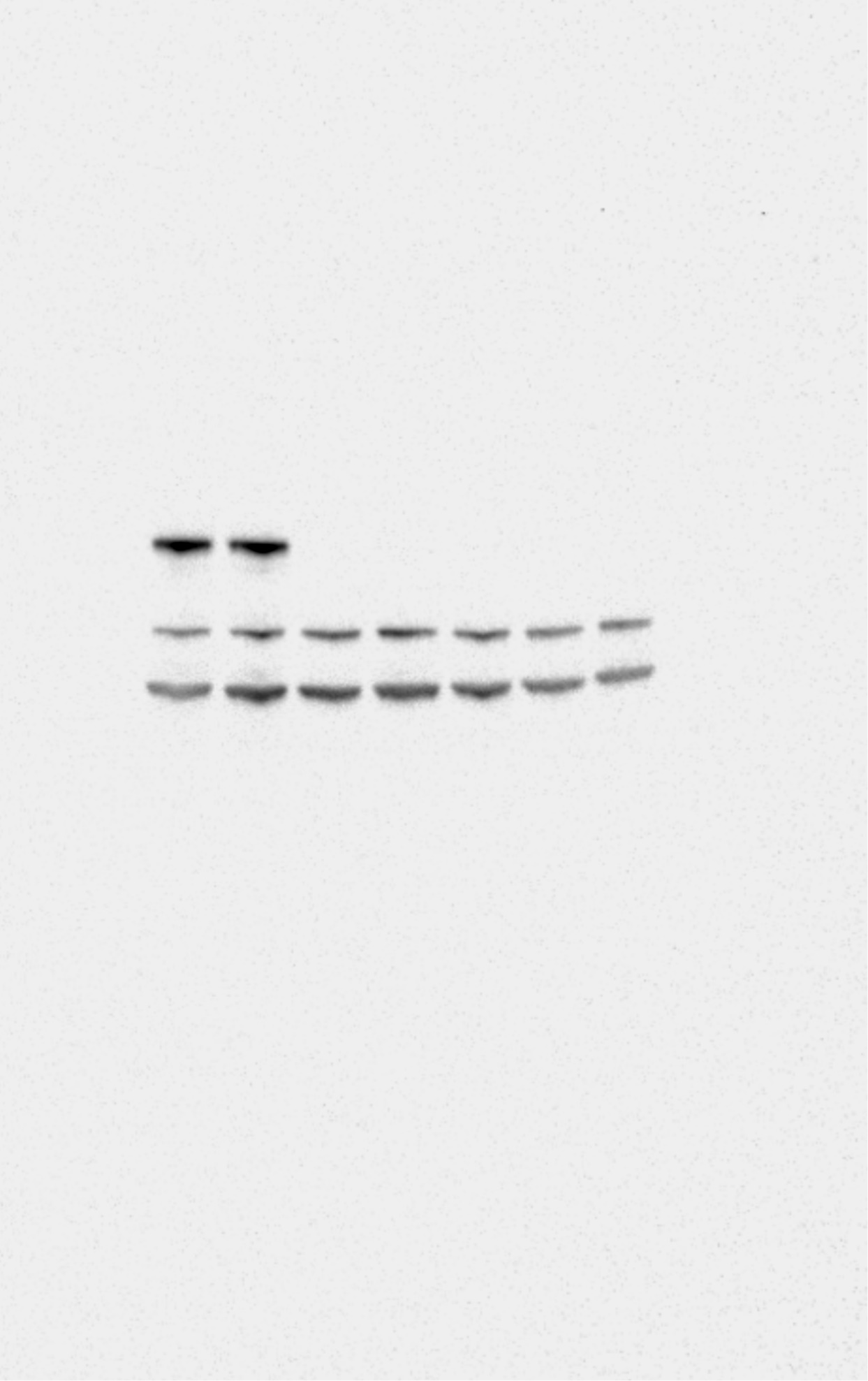


Fig S8 BActin_5seconds_1_LHS_2_RHS_20221215 CP


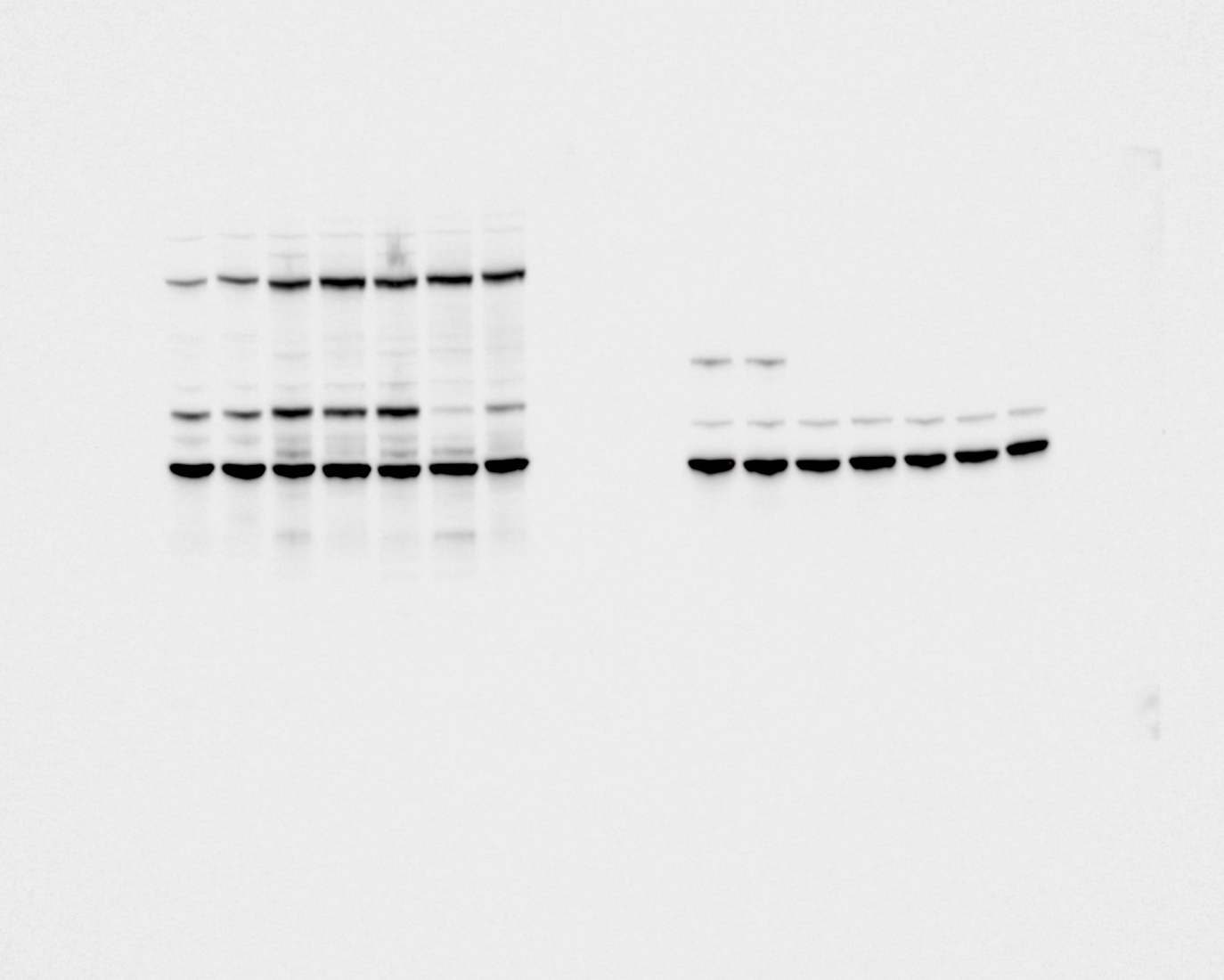

Supplement: Supplementary file 10 — Additional file 10. Uncropped images for the blots in figure 1, additional file 2: supplementary figure s1, s6-8. [file 13059_2023_3161_MOESM10_ESM.docx]
