# Supplementary material for: The androgen receptor interacts with GATA3 to transcriptionally regulate a luminal epithelial cell phenotype in breast cancer
Source: Genome Biol. 2024 Feb 5;25:44. doi: 10.1186/s13059-023-03161-y (PMC10840202; doi:10.1186/s13059-023-03161-y)
Supplement: Supplementary file 11 — Additional file 11. Review history. [file 13059_2023_3161_MOESM11_ESM.docx]

Review History

**First round of review**

**Reviewer 1**

**Were you able to assess all statistics in the manuscript, including the appropriateness of statistical tests used?**

No, I do not feel adequately qualified to assess the statistics.

**Comments to author:**

The Androgen receptor (AR) is frequently expressed and acts as a tumor suppressor in estrogen receptor (ER) positive breast cancer. However, the potential collaborating factors that may enhance AR genomic activities and functions in both ER positive and negative breast cancer models remain unclear. In this study, Hosseinzadeh et al. explored the AR interactome without bias, employing the chromatin immunoprecipitation-based proteomic technique (RIME), and identified GATA3 as a novel AR cofactor in four breast cancer cells, irrespective of ER status. Using a combination of cell lines and a patient-derived xenograft (PDX) tumor, the authors demonstrated that AR activation by 5α-dihydrotestosterone (DHT) enhanced the interaction between AR and GATA3 on chromatin. This interaction led to a significant enrichment of GATA3 on a subset of genomic regions that were co-occupied by AR with increased binding intensity. Intriguingly, while ER activation also induced GATA3 bindings, the extent was substantially lesser compared to that stimulated by DHT. Moreover, the study observed that the increased GATA3 bindings were AR-dependent, and the loss of GATA3 diminished - but did not completely eradicate AR transcriptional activities, thereby further strengthening the role of GATA3 as an AR co-activator. Finally, integration of ChIP-seq with RNA-seq data following DHT treatment revealed androgen-regulated AR-GATA3 binding sites associated with genes that promoted a luminal epithelial phenotype in both ER+ and ER- breast cancer contexts. Overall, this is a commendable study that establishes for the first time the genomic and functional implications of AR-GATA3 interactions in breast cancer. A couple of points should be addressed by the authors:

1. Could the authors provide additional experimental and clinical evidence to reinforce the functions of the two key AR-GATA3 regulated genes (i.e., EHF, KDM4B) in breast cancer?

2. In line 122, "supplemental Fig 1E" should be revised to "Fig 1F".

**Reviewer 2**

**Were you able to assess all statistics in the manuscript, including the appropriateness of statistical tests used?**

Yes, and I have assessed the statistics in my report.

**Comments to author:**

The androgen receptor (AR) is a tumor suppressor in estrogen receptor (ER) positive breast cancer. The group has previously supported this paradigm in a recent publication (Nature Medicine 2021).

To dissect the mechanisms involved in AR-induced tumor suppression, they employ an unbiased chromatin immunoprecipitation-based proteomic technique (RIME) to identify and compare endogenous AR interacting proteins in ER-positive and negative models of breast cancer.

Among the proteins identified, they focused on a protein shared in all four cell lines tested: the DNA-binding factor GATA3.

GATA3 was validated as a novel AR interacting protein in breast cancer cells irrespective of ER status. They found that AR activation by the 5α-dihydrotestosterone (DHT) increased nuclear AR-GATA3 interactions, resulting in AR-dependent enrichment of GATA3 chromatin binding at a sub-set of genomic loci. Silencing GATA3 reduced but did not prevent AR DNA binding and transactivation of genes associated with AR/GATA3 co-occupied loci, indicating a co-regulatory role for GATA3 in AR signaling. Silencing of GATA3 prevented the induction of EHF and KDM4, the two genes validated as important downstream activated targets and responsible for the pro-differentiation effects observed.

Overall, the approaches implemented are accurate, and the manuscript is well-written and clear.

The notion that AR has a tumor suppressor role in breast cancer is broadly accepted. However, it is relevant to understand how the AR selectively executes a tumor suppressive transcriptional program and the co-factors involved. Previous publications have shown the correlation between GATA3 and AR. The novelty here is the physical interaction at the chromatin level and the regulatory consequences. However, there are gaps that should be addressed to provide relevant insights into this newly discovered interaction. Most of the weaknesses are related to the functional relevance of the connection between AR and GATA3 and the contribution of GATA3 to the tumor suppressor consequences of AR activation.

Specific comments

The authors show consensus GATA3/AR loci: it would be supportive to show the genes involved at the gained foci in the model tested and point to the convergence and divergence between the different cell models. Moreover, functional annotation of the genes putatively affected (GATA3/AR co-bound loci) should be included.

It would be informative to define the genomic areas involved (peaks annotation) in the consensus/co-bound loci. Functional annotation analysis of the genes involved by the induction in the four cell lines upon manipulation should also be included

To understand the functional impact of AR/GATA3 interaction on the tumor suppressor role of AR in breast cancer cells, the authors should perform functional assays on the cell lines and PDX tested following GATA3 manipulation. No data supports the reversion of the malignant phenotype by AR/GATA3 interaction in all the cell lines included. However, to what extent GATA3 interaction with AR contributes to the malignant reversion by AR is not clear. To this end, the authors can implement cells and organoids from PDX to evaluate the luminal reversion upon AR and GATA3 manipulation. This is important to establish that GATA3 is relevant for the AR-induced reversion of the malignant phenotype.The induction of luminal differentiation with enhanced EHF and KDM4B expression is of interest but is insufficient to determine the relevance of GATA3.

Moreover, to understand the transcriptional impact of the AR/GATA3 interaction, the authors should perform RNA sequencing in cell lines induced by DHT and transfected with and without GATA3 silencing.

The integration of the ChIP seq data with RNA seq data performed upon DHT induction (in ER negative cells) (ER+positive cells),is not sufficient to define the transcriptional impact of GATA3.

GATA3 is important for the induction of luminal pro-differentiation genes, but to what extent GATA3 ablation would prevent the tumor suppressor effects of AR in breast cancer is not clear.

In each cell line, many interacting proteins were identified. These data support that the role of AR might be divergent in each cell context based on different co-factors. The authors should comment on this point.

For this reason, evaluating the functional consequences of AR induction in cell lines might be relevant to establish the protective/tumor suppressor role of GATA3 in each cell context.

**Authors Response**

**Point-by-point responses to the reviewers’ comments:**

Reviewer #1:

The Androgen receptor (AR) is frequently expressed and acts as a tumor suppressor in estrogen receptor (ER) positive breast cancer. However, the potential collaborating factors that may enhance AR genomic activities and functions in both ER positive and negative breast cancer models remain unclear. In this study, Hosseinzadeh et al. explored the AR interactome without bias, employing the chromatin immunoprecipitation-based proteomic technique (RIME), and identified GATA3 as a novel AR cofactor in four breast cancer cells, irrespective of ER status. Using a combination of cell lines and a patient-derived xenograft (PDX) tumor, the authors demonstrated that AR activation by 5α-dihydrotestosterone (DHT) enhanced the interaction between AR and GATA3 on chromatin. This interaction led to a significant enrichment of GATA3 on a subset of genomic regions that were co-occupied by AR with increased binding intensity. Intriguingly, while ER activation also induced GATA3 bindings, the extent was substantially lesser compared to that stimulated by DHT. Moreover, the study observed that the increased GATA3 bindings were AR-dependent, and the loss of GATA3 diminished - but did not completely eradicate AR transcriptional activities, thereby further strengthening the role of GATA3 as an AR co-activator. Finally, integration of ChIP seq with RNA-seq data following DHT treatment revealed androgen-regulated AR-GATA3 binding sites associated with genes that promoted a luminal epithelial phenotype in both ER+ and ER- breast cancer contexts. Overall, this is a commendable study that establishes for the first time the genomic and functional implications of AR-GATA3 interactions in breast cancer. A couple of points should be addressed by the authors.

*Response: We thank Reviewer #1 for recognizing the importance of our study identifying GATA3 as a novel AR cofactor in ER+ and ER- breast cancer contexts as well as providing insightful suggestions on how to improve the manuscript. We have edited the manuscript to address the points raised, as outlined below.*

Minor comments:

1) Could the authors provide additional experimental and clinical evidence to reinforce the functions of the two key AR-GATA3 regulated genes (i.e., EHF, KDM4B) in breast cancer?

*Response: To provide clinical evidence for the function of EHF and KDM4B in breast cancer, we performed survival analyses using the KM plotter mRNA database to examine whether expression of these genes was associated with better patient outcomes. The analyses assessed all breast cancer cases as well as the ER+ and ER- subgroups of disease. We did not find a significant association between EHF mRNA expression and breast cancer outcomes under any category, but higher levels of KDM4B mRNA were significantly associated with better outcomes when considering all cases and ER+ cases as a subgroup (new Supplemental Figure 8E). Higher levels of KDM4B trended toward better outcomes in the ER- subgroup, but divergence of the curves was not significant, likely due to low numbers of cases available for assessment in this category. This new data regarding KDM4B accords with a study (new reference #75) published after submission of our manuscript that also shows higher levels of KDM4B associated with better breast cancer outcomes. Collectively, this clinical data supports the concept that KDM4B is a good prognostic factor, like both AR and GATA3. Although EHF expression was not associated with patient outcomes in our new survival analyses, two clinical studies show that EHF is only expressed in differentiated luminal epithelial cells of the human mammary gland and that expression is decreased or lost in pre-cancerous breast lesions (new references #77-78). This clinical data supports a role for EHF in promoting differentiation of the luminal lineage of mammary epithelial cells and is consistent with the finding that EHF is a key transcription factor that distinguishes luminal from basal progenitor cells in the human mammary gland (original reference #38).*

*In terms of functional roles for EHF and KDM4B, two recently published papers (new references #79, #81) and one recently posted preprint (17th October 2023) (new reference #80) have provided evidence using knockdown and overexpression approaches that EHF and KDM4B have pro-differentiation, tumor suppressive roles in the context of breast cancer. One study (new reference #79) focused on head and neck cancer but also performed in vitro and in vivo experiments with MDA-MB-231 cells, a model of triple negative breast cancer. That study and the study in a newly posted pre-print (new reference #80) employed overexpression and knockdown experiments with triple negative breast cancer cells to show that EHF had tumor suppressor actions via induction of apoptosis, inhibition of metastasis, and sensitization of cells to chemotherapy. Another study (new reference #75) employed overexpression and knockdown experiments to demonstrate that KDM4B inhibits proliferation and metastasis of ER+ and ER- breast cancer cells.*

*Collectively, our new analyses and recently published data strongly support our conclusion that AR/GATA3 mediated up-regulation of EHF and KDM4B is part of a pro-differentiation, tumor suppressive gene regulatory program. We have made changes to the revised manuscript at the following locations: Lines 300-303; 439-443; 446-447; 450; 454-461; and 463-464 of the revised manuscript.*

2) In line 122, "supplemental Fig 1E" should be revised to "Fig 1F”.

*Response: We thank Reviewer #1 for pointing out this oversight, which has been corrected in the revised manuscript (line 124 of the revised manuscript).*

Reviewer #2:

The androgen receptor (AR) is a tumor suppressor in estrogen receptor (ER) positive breast cancer. The group has previously supported this paradigm in a recent publication (Nature Medicine 2021). To dissect the mechanisms involved in AR-induced tumor suppression, they employ an unbiased chromatin immunoprecipitation-based proteomic technique (RIME) to identify and compare endogenous AR interacting proteins in ER-positive and negative models of breast cancer.

Among the proteins identified, they focused on a protein shared in all four cell lines tested: the DNA-binding factor GATA3. GATA3 was validated as a novel AR interacting protein in breast cancer cells irrespective of ER status. They found that AR activation by the 5α-dihydrotestosterone (DHT) increased nuclear AR-GATA3 interactions, resulting in AR-dependent enrichment of GATA3 chromatin binding at a sub-set of genomic loci. Silencing GATA3 reduced but did not prevent AR DNA binding and transactivation of genes associated with AR/GATA3 co-occupied loci, indicating a co-regulatory role for GATA3 in AR signaling. Silencing of GATA3 prevented the induction of EHF and KDM4B, the two genes validated as important downstream activated targets and responsible for the pro-differentiation effects observed.

Overall, the approaches implemented are accurate, and the manuscript is well-written and clear.

The notion that AR has a tumor suppressor role in breast cancer is broadly accepted. However, it is relevant to understand how the AR selectively executes a tumor suppressive transcriptional program and the co-factors involved. Previous publications have shown the correlation between GATA3 and AR. The novelty here is the physical interaction at the chromatin level and the regulatory consequences. However, there are gaps that should be addressed to provide relevant insights into this newly discovered interaction. Most of the weaknesses are related to the functional relevance of the connection between AR and GATA3 and the contribution of GATA3 to the tumor suppressor consequences of AR activation.

*Response: We thank Reviewer #2 for their positive comments about the importance of our study for the field and for providing constructive advice for improvement of the manuscript. While this study initially focused more on elucidating a conserved role for AR in promotion of a luminal phenotype across all breast cancer subtypes via interaction with GATA3, we have revised the manuscript to provide further evidence and better highlight what consequences this has for AR-mediated tumor suppression. In addressing Reviewer #1’s comment, we have provided stronger evidence from our newly performed survival analyses as well as recently published studies demonstrating that EHF and KDM4B have tumor suppressor roles in breast cancer, which supports our conclusion that AR/GATA3 mediated up-regulation of these transcription factors contributes to a tumor suppressive regulatory program in ER+ and ER- breast cancer cells. In addition, we have performed new experiments and bioinformatic analyses and reworked the manuscript as indicated below to address specific comments concerning the potential contribution of GATA3 to AR tumor suppressive activity, which overall aids in interpretation of the manuscript and strengthens our conclusions.*

Specific comments:

1) The authors show consensus GATA3/AR loci: it would be supportive to show the genes involved at the gained foci in the model tested and point to the convergence and divergence between the different cell models. Moreover, functional annotation of the genes putatively affected (GATA3/AR co-bound loci) should be included. It would be informative to define the genomic areas involved (peaks annotation) in the consensus/co-bound loci. Functional annotation analysis of the genes involved by the induction in the four cell lines upon manipulation should also be included.

*Response: The revised manuscript includes a new Additional File 3 reporting the AR-GATA3 co-occupied binding sites induced by DHT and annotated to the nearest gene. We have also added gene annotations to Additional File 2. The revised manuscript includes a pathway analysis performed on genes associated with DHT-induced AR/GATA3 binding sites in ER+ and ER- contexts, which shows a common enrichment of differentiation and development pathways (Additional File 5, lines 273-275 of the revised manuscript). To address the convergence and divergence of AR/GATA3 binding sites between the four cell line models, we performed additional analyses (Supplemental Figure S5H-I of the revised manuscript) comparing DHT-induced AR/GATA3 DNA binding patterns between cell lines and overlap of the associated annotated genes. These data show that while there are some model-specific binding patterns, the AR/GATA3 binding sites shared among models were the strongest and that most genes associated with these AR/GATA3 regulatory loci were common across all models. Hence, these genomic events are more likely to have biological significance because they are not model specific, a point which we have made in lines 206-211 of the revised manuscript. These additions have increased the informative value of the cistromic data in this manuscript.*

1.1) To understand the functional impact of AR/GATA3 interaction on the tumor suppressor role of AR in breast cancer cells, the authors should perform functional assays on the cell lines and PDX tested following GATA3 manipulation. No data supports the reversion of the malignant phenotype by AR/GATA3 interaction in all the cell lines included. However, to what extent GATA3 interaction with AR contributes to the malignant reversion by AR is not clear.

*Response: A fundamental problem in silencing GATA3 to perform functional assays in the context of ER+ breast cancer is that like ER, GATA3 is an essential gene (see Supplemental Figure S6H of the revised manuscript and response to Comment #2 below). Therefore, to address your comment and get around this problem, we assessed whether a dose of AR agonist that does not inhibit proliferation of ER+ breast cancer cells was able to induce AR/GATA3 interactions. We used a selective AR modulator (SARM) called enobosarm, which we have shown has AR agonist action in ER+ breast cancer cells and inhibits proliferation at a 100 nM but not 10 nM dose (original reference #7). Our new data (Supplemental Figure S6F) shows that only a growth inhibitory dose of enobosarm (100 nM) induced AR/GATA3 interactions in the ER+ T-47D breast cancer cell line. There was no evidence of AR/GATA3 interaction at the lower dose (10 nM) of enobosarm, but the 100 nM dose induced a degree of interaction nearly equivalent to that induced by 10 nM of DHT. These new data support the conclusion that AR/GATA3 interactions are important for AR-mediated tumor suppression in an ER+ breast cancer context.*

*To address this point in the context of ER- breast cancer, we performed new experiments in which we knocked down GATA3 and assessed the proliferative response to DHT in MFM-223 and MDA-MD-453 cell lines. It is important to note that while AR unequivocally acts as a tumor suppressor in ER+ breast cancers, the role and mechanistic pathways associated with AR signaling in ER- disease are more obscure and likely to differ depending on the molecular subtype. This is exemplified by the representative ER- cell line models used in our study, whereby MDA-MB-453 (molecular apocrine/HER2+) cell proliferation is variably influenced by AR activation depending on culture conditions (Hall et al., Eur J Cancer, 1994; Birrell et al., J Steroid Biochem Mol Biol, 1995; Wang et al., Oncogene, 2011), whereas MFM-223 (triple negative breast cancer) cell proliferation is invariably inhibited by AR activation (Hackenberg et al., Cancer Res, 1991; Hickey et al., Mol Endocrinol, 2012). We have added text in the revised manuscript to point out that despite comparable expression of AR and GATA3 in MDA-MB-453 and MFM-223 cells, DHT stimulated a much higher level of AR/GATA3 interaction in the MFM-223 cells, which is consistently and potently growth inhibited by AR signaling. In the new experiments, we found that silencing GATA3 completely abolished proliferation of MDA-MB-453 and MFM223 cells, indicating GATA3 is an essential gene in the context of ER- as well as ER+ breast cancer. We confirmed this by interrogation of the Cancer Dependency Map (DepMap) resource (see response to Comment #2 below). Nonetheless, our new data shows that treatment with DHT rescued proliferative capacity in MDA-MB-453 cells that lacked GATA3 expression, but this did not occur in the MFM223 cells (Supplemental Figure S6G). The new data provides supporting evidence that the AR/GATA3 interaction has anti-proliferative consequences in both models, but this tumor suppressive activity is more robust in the MFM223 model. We speculate that AR has unique pro-tumorigenic potential in MDA-MB-453 cells due to interaction with FOXA1, as reported by us previously (Robinson et al., EMBO J, 2011). Loss of GATA3 in MDA-MB-453 cells likely ‘releases’ AR to more fully interact with FOXA1, as discussed in the revised manuscript (lines 422-428). Formal testing of this mechanism is beyond the scope of the current manuscript but is the subject of ongoing study.*

*Collectively, the new data generated for the revised manuscript in response to Comment 1 supports the conclusion that GATA3 is a critical co-factor for AR tumor suppressive action in ER+ and ER- breast cancer contexts. We have revised the manuscript text accordingly to incorporate these new data and their implications (lines 236-261; 311; 326-327; 370-372; 422-428).*

1.2) To this end, the authors can implement cells and organoids from PDX to evaluate the luminal reversion upon AR and GATA3 manipulation. This is important to establish that GATA3 is relevant for the AR-induced reversion of the malignant phenotype. The induction of luminal differentiation with enhanced EHF and KDM4B expression is of interest but is insufficient to determine the relevance of GATA3.

*Response: The Reviewer’s suggestion to explore reversion of a luminal phenotype in organoids is an interesting idea though one that likely necessitates a single-cell/single-nuclei sequencing approach due to the heterogeneity of cell populations in these models, which could not be performed in a realistic timeframe for the revision of the current manuscript. Moreover, organoids represent highly "selected" models that do not necessarily retain certain key characteristics of the parental tissue (whether primary breast tissue or PDX). Indeed, we are currently characterizing AR signaling in organoids of ER+ and ER- breast cancer developed by Prof. Alana Welm at the Huntsman Cancer Institute, Utah (described in Guillen et al, Nature Cancer 2022). We have found in the ER+ organoid models that expression and activity of ER and AR is markedly diminished compared to the parental tissue of origin and have only just begun investigation of AR expression and signaling the in the ER- organoid models. This work forms part of an independent investigation that will ultimately complement the body of work presented in the current manuscript.*

2) Moreover, to understand the transcriptional impact of the AR/GATA3 interaction, the authors should perform RNA sequencing in cell lines induced by DHT and transfected with and without GATA3 silencing. The integration of the ChIP seq data with RNA seq data performed upon DHT induction (in ER negative cells) (ER+positive cells), is not sufficient to define the transcriptional impact of GATA3.

*Response: We agree with this point and hence performed targeted ChIP-PCR and RT-PCR experiments following AR or GATA3 silencing in both ER+ and ER- cell lines to determine the transcriptional impact of GATA3 on a subset of canonical AR target genes (e.g., ZBTB16, SEC14L2; Figure 5), as well as luminal identity genes (e.g., KDM4B, EHF; Supplemental Figure S7, 8). We did not explore the global transcriptional impact of GATA3 silencing in ER+ or ER- contexts because GATA3 has a role that extends beyond the role it plays in mediating ER or AR signaling in breast cancer cells. In part, this is supported by the fact that the GATA3 cistromes in breast cancer cells do not fully overlap the ER or AR cistromes. In addition, the Cancer Dependency Map indicates that all four cell lines used in this study, irrespective of ER status, are highly dependent on GATA3 (i.e., have a negative dependency score) (Supplemental Figure S6H of the revised manuscript), which is in concordance with previous CRISPR screens in ER+ breast cancer cells (Fei et al., 2019 PNAS; Nagarajan et al., 2020 Nat Genetics) and our new data in ER- breast cancer cells (Supplemental Figure S6G). The targeted ChIP-PCR and RT-PCR experiments support the conclusion that both AR and GATA3 are essential for transcriptional regulation of luminal identity genes.*

3) GATA3 is important for the induction of luminal pro-differentiation genes, but to what extent GATA3 ablation would prevent the tumor suppressor effects of AR in breast cancer is not clear.

*Response: As per our response to points #1 and #2 above, this approach is hampered by the essential nature of GATA3 in breast cancer cells. The best approach would be to specifically prevent interaction between AR and GATA3 without influencing their expression or independent function. We are currently pursuing this via peptidomimetics that may block the interaction and by seeking mutations of AR or GATA3 that preclude their interaction but not their individual function. It is not feasible to complete this work in a timely manner to include in the current manuscript.*

4) In each cell line, many interacting proteins were identified. These data support that the role of AR might be divergent in each cell context based on different co-factors. The authors should comment on this point.

*Response: The Reviewers’ point is well taken, and consequently we have edited the manuscript (lines 93; 358-372; 405-431 of the revised manuscript) to describe in more detail other co-factors identified that could underpin a distinct role for AR in these different breast cancer contexts. In the Discussion (lines 405-431), we highlight the fact that FOXA1 was only detected as a high confidence AR interacting protein in the MDA-MB-453 cell line, despite comparable FOXA1 expression in all models (Supplemental Figure S8F). This raises the possibility that the interaction between AR and FOXA1 could explain why the MDA-MB-453 cell line is the only cell line investigated, irrespective of ER status, in which AR activation can promote rather than inhibit proliferation. We intend to pursue this intriguing observation, but it is peripheral to and outside the scope of the current manuscript.*

5) For this reason, evaluating the functional consequences of AR induction in cell lines might be relevant to establish the protective/tumor suppressor role of GATA3 in each cell context.

*Response: As outlined above, we have addressed this point by performing additional in vitro experiments, which collectively support a role for GATA3 in facilitating the tumor suppressor function of AR in ER+ and ER- breast cancer cells.*

**Second round of review**

**Reviewer 1**

The authors have addressed my concerns. The manuscript is ready for publication.

**Reviewer 2**

The authors have satisfactorily answered my previous comments and provided new data accordingly. The manuscript in this form is acceptable.
